# Seasonality of lake microbial denitrification and its sensitivity to climate warming

**DOI:** 10.1038/s41564-026-02349-9

**Published:** 2026-05-22

**Authors:** Cameron M. Callbeck, Alessandra Mazzoli, Tim J. Paulus, Claudia Frey, Helmut Bürgmann, Carsten J. Schubert, Moritz F. Lehmann

**Affiliations:** 1 Department of Environmental Sciences, University of Basel, Basel, Switzerland; 2 Department of Surface Waters—Research and Management, Eawag, Swiss Federal Institute of Aquatic Science and Technology, Kastanienbaum, Switzerland; 3 Institute of Biogeochemistry and Pollutant Dynamics, ETH, Zurich, Switzerland

**Keywords:** Freshwater ecology, Biogeochemistry, Ecological modelling, Ecophysiology

## Abstract

Lakes are expected to experience longer summer stratification and shorter winter mixing due to climate-induced warming. These changes will impact biogeochemical cycles, but how shifts in mixing might influence lake nitrogen removal via denitrification remains unconstrained. Here we used ^15^N-tracer assays, molecular techniques and flux measurements to establish the seasonal dynamics of denitrification in a eutrophic lake in Switzerland. We find that denitrification was disproportionately active during the winter mixed regime, potentially driven by a previously unrecognized chitinolytic–denitrifying microbial consortium. Moreover, denitrification was strongly governed by the relative availabilities of particulate organic carbon and nitrate. Leveraging these insights enabled accurate simulation of denitrification in a lake model, revealing that a worst-case climate scenario may shorten the mixing period by ~27 days and reduce denitrification by 8–13%, increasing nitrogen export to downstream ecosystems. We conclude that lake microbial denitrification, and its associated denitrifying consortium, will be weakened by climate change.

## Abstract (web summary)

Denitrification is an essential process for the removal of excess nitrogen in aquatic ecosystems, and models informed by lake samples suggest that seasonal stratification changes could weaken the microbial communities responsible for this process.

## Main

In the global nitrogen (N) budget, freshwater ecosystems comprising lakes, rivers, streams and their estuaries contribute to 21% of denitrification^1^. Particularly, the upper few millimetres of lake sediments at the oxic–anoxic transition are where fixed nitrogen cycling and its removal via dinitrogen gas production are most active. Here microbial remineralization of organic nitrogen to ammonium together with nitrification, denitrification and anaerobic ammonium oxidation (anammox) catalyses the removal of fixed nitrogen to dinitrogen gas^2–6^. Alternatively, organic matter remineralization can couple with nitrification and dissimilatory nitrate reduction to ammonium (DNRA), recycling fixed nitrogen back to ammonium. The pathways for recycling and/or removing fixed nitrogen co-occur in sediments^6–9^, with evidence suggesting that DNRA becomes more important when organic carbon is high relative to nitrate^10^, and under more reducing conditions^11^. Despite the importance of these microbial processes in moderating the nitrogen balance in freshwater ecosystems, uncertainties persist regarding their potential responses to projected global temperature increases of 0.1–0.4 °C per decade^12^.

Meta-analyses have shown that elevated temperatures can directly impact denitrification by augmenting enzymatic turnover rates^13^. Another indirect avenue for change may arise from enhanced water column stratification as lakes warm. Indeed, warming surface waters are expected to alter the timing of winter mixing (phenology) in monomictic lakes—lakes that mix once per year—while lengthening the summer stratification period by several days to weeks^14–17^. Given the interconnectivity of nitrogen and carbon cycles and their sensitivity to dissolved oxygen concentrations^18–21^, it is envisaged that shifts in the stratification phenology will inevitably impact the nitrogen cycle via the other linked element cycles. One possibility is that prolonged stratification could expand the niche for phytoplankton, increasing the organic matter flux to the sediment and enhancing anoxia—conditions that would favour DNRA at the detriment of denitrification. Conversely, the seasonal mixed regime reoxygenates the water column and may represent a key window for fixed-N removal via nitrification–denitrification; if this window shrinks with warming, the lake’s overall denitrification capacity could decline. However, how shifts in the phenology of water column stratification and mixing induced by climate change influence nitrogen cycling processes in monomictic lakes remains unconstrained.

Addressing this knowledge gap requires a rudimentary understanding of how nitrogen cycling, both in the water column and within the sediments, operates in mixed versus stratified environments. A collation of aquatic studies finds that processes such as organic matter remineralization, anammox, nitrification and nitrogen fixation show substantial variations between mixed and stratified conditions^22–25^. Furthermore, the nitrate-consuming processes of DNRA and denitrification seemingly show opposing responses to stratification and mixing. Some evidence indicates that DNRA prevails in the stratified regime^26^, while denitrification activity reaches its zenith in the mixed regime^19,27,28^. In line with this, other studies point to enhanced denitrification under artificially mixed conditions^29,30^. In addition, metagenomic-based surveys reveal that the functional dynamics of nitrogen cycling processes (as proxied by the *nirK* to *nirS* ratio) vary between mixing regimes^31^. This amalgamation of aquatic studies broadly affirms that the hydrodynamic regime is key to moderating nitrogen cycling dynamics. However, in the context of monomictic lakes, these dynamics are yet to be comprehensively catalogued.

To address these prevailing knowledge gaps, this study aimed to first elucidate the drivers of denitrification in a representative monomictic lake. Specifically, we used a suite of ^15^N stable-isotope addition experiments to quantify the benthic and water column activities of the various nitrogen transformation processes with a focus on determining how benthic denitrification is influenced by the mixing regimes. Paired with our biogeochemical analysis, we examined the key microbial players involved to gain further insight into what powers denitrification under contrasting hydrodynamic regimes. We then used in situ trends to better simulate denitrification and DNRA in silico, using as our framework a well-established hydrodynamic–biogeochemical coupled lake model. Lastly, we investigated how shifting stratification phenology induced by various anthropogenic greenhouse gas emission scenarios: Representative Concentration Pathway (RCP) 2.6 (low-emission) and 8.5 (high-emission), impacts lake nitrogen removal and recycling rates by differentially affecting denitrification and DNRA.

## Results

### Nitrogen transformation rates in Baldeggersee

**Fig. 1:**
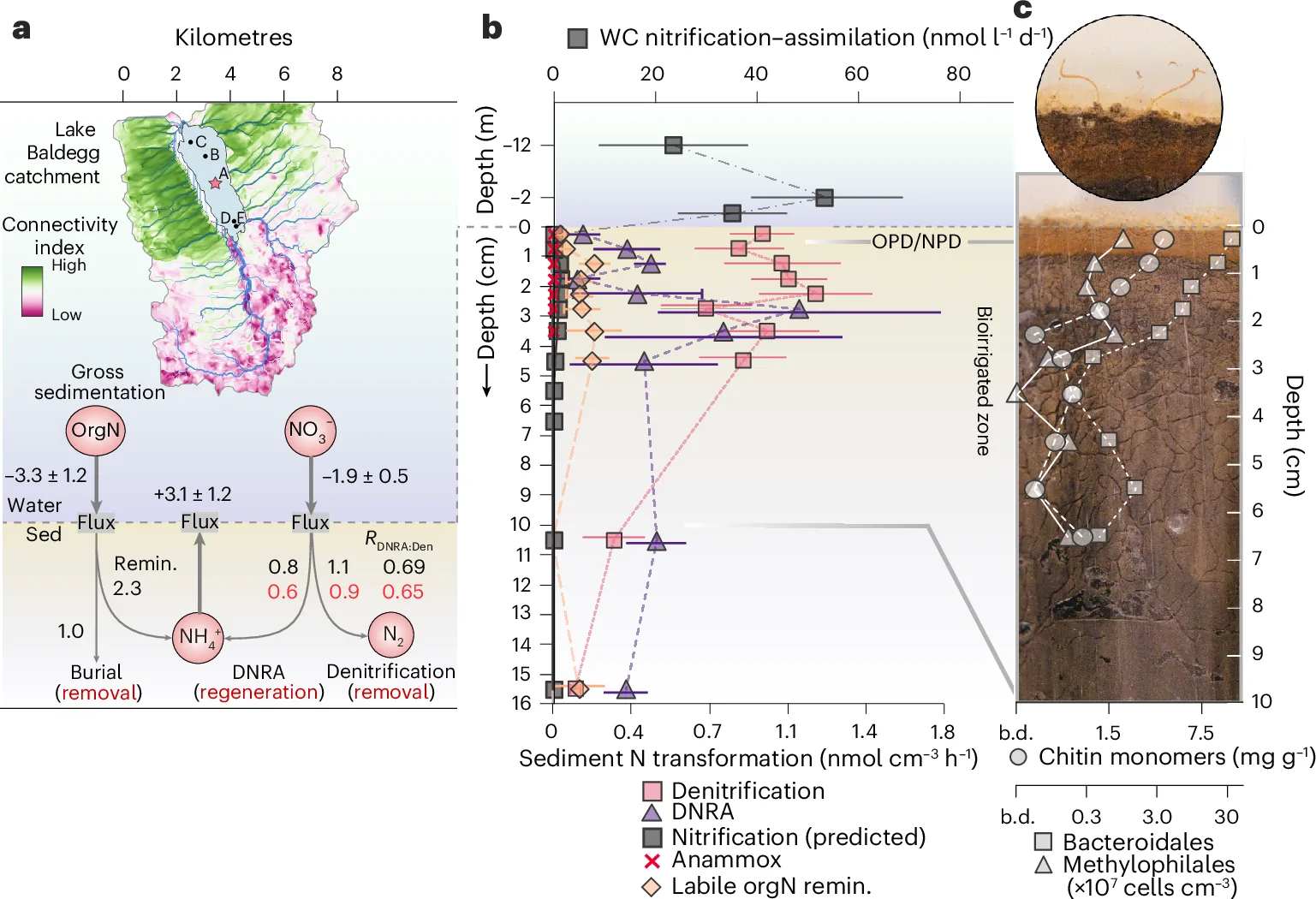
Vertical distribution of potential nitrogen transformation processes in Baldeggersee (Lake Baldegg). **a**, The lake (in blue), including the sampled stations, is shown. The deepest station (A) is indicated by the star, where most analyses were performed (Supplementary Table 1); additional inlet and outlet stations are indicated by circles (B–E). The panel also includes the lake catchment and its topographical surroundings. The river catchment network is overlaid with the connectivity index, indicating the connection of hillslopes to the catchment region. Green represents arable land on hillsides with high connectivity to the catchment, whereas red denotes low-connectivity areas, occupied by cityscapes. Below the map, schematic representations of water and benthic fluxes (data from ref. ^19^) used to estimate areal rates of denitrification and DNRA (mmol m^−2^ d^−1^; shown in black) are presented. The DNRA to denitrification ratio (*R*_DNRA:Den_) is also shown. Potential denitrification and DNRA rates shown in red are derived from ^15^NO_3_^−^ tracer incubations. These values were calculated from mean volumetric seasonal rates, depth-integrated over the upper 0–5 cm. Benthic flux values represent seasonal means, with error bars indicating the standard deviation. **b**, Potential rates of organic nitrogen remineralization (OrgN remin.), anammox, DNRA and denitrification in the sediment (Sed) (nmol cm^−3^ h^−1^) were quantified using ^15^N stable-isotope addition experiments. Data are presented as mean values ± s.e.m. (*n* = 6; seasonal data). Predicted sediment nitrification activity was extrapolated from enumerated Thaumarchaeota (using CARD-FISH), combined with single-cell rates from a previous study^24^. Water column (WC) nitrification–assimilation rates (nmol l^−1^ d^−1^) were determined from ^15^NH_4_^+^ addition experiments, in which consumption of ^15^NH_4_^+^ was monitored over time (Extended Data Fig. 2). Oxygen and nitrate-penetration depths (OPD and NPD) are denoted according to microsensor and microrhizon concentration profiles shown in Extended Data Fig. 1. **c**, Photograph illustrating the extent of bioirrigation in the sediments. Baldeggersee sediments are dominated by *Tubifex* worms, with roughly 8,500 individuals per square metre that cause sediment mixing and water and nutrient exchange within the bioirrigation zone^45^. Overlaid on the image are the vertical abundances of Bacteroidales and Methylophilales (enumerated by CARD-FISH), as well as concentration profiles of chitin-related monomers (glucosamine and *N*-acetylglucosamine, *m*/*z* = 178 and 220, respectively). b.d., below detection. Source data

Baldeggersee, an extensively monitored lake in central Switzerland, is located within an agriculture-dominated catchment region (Fig. 1a). Over the preceding decades, the lake has undergone remediation, including phosphorus removal from incoming wastewater, as well as the deployment of oxygen and air diffusers to improve lake aeration^32^. While remediation efforts have lowered its trophic status from hyper-eutrophic to eutrophic, the lake presently still has high ambient water column nitrate concentrations (124 ± 26 μM). Eutrophic lakes such as Baldeggersee are becoming increasingly prevalent as nutrient enrichment from agriculture and urbanization intensifies. This background nitrate level sustains nitrate fluxes (1.9 ± 0.5 mmol m^−2^ d^−1^) threefold higher than that in oligotrophic Swiss lakes^19,33^, while nutrient-rich conditions drive summer phytoplankton blooms that generate particulate organic matter sedimentation rates twice those observed in oligotrophic lakes^19^. The combined organic and inorganic fluxes of nitrogen maintain a highly active nitrogen cycle in the upper sediments of Baldeggersee^31,33^. In the sediments, roughly half of the fixed nitrogen is removed through the microbial benthic nitrogen cycle, with the other half eliminated via organic nitrogen burial^19^ (Fig. 1a).

**Extended Data Fig. 1:**
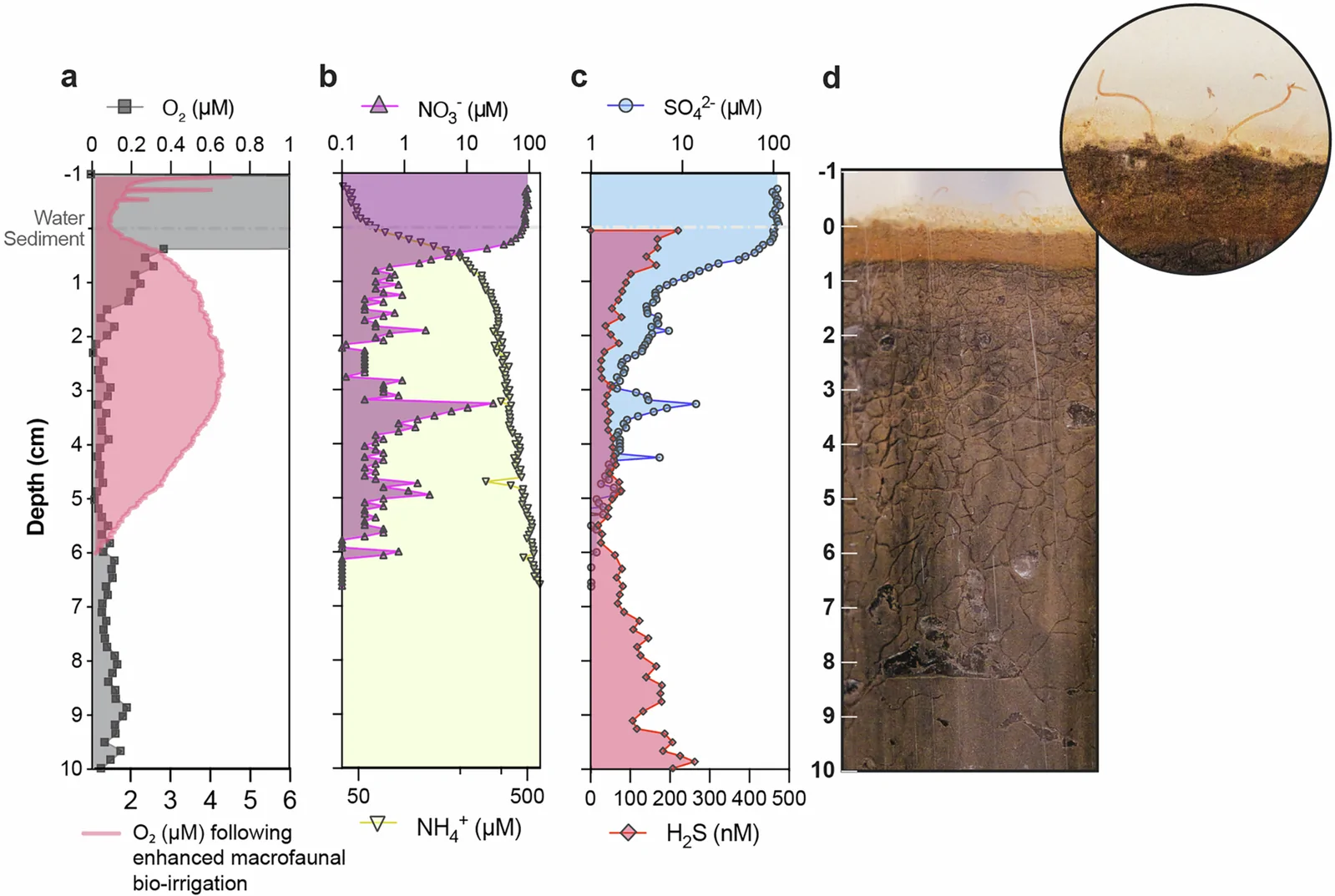
Chemical depth profiles and sediment-dwelling macrofauna in Baldeggersee. (**a,b,c)** Example plots of oxygen, nitrate/ammonium, and sulfate/sulfide concentration profiles are shown. (**a**) In situ oxygen profile from a recovered sediment core is shown alongside a profile from a core subsequently spiked with additional macrofauna, demonstrating their potential to transport oxygen to deeper layers of the sediment. (**d**) Tubifex worms (top right panel) create dense burrow networks spanning from the superficial sediment layer down to ∼10 cm. In Baldeggersee, Tubifex worms occur at a faunal density of 8,500 individuals per m^2^. The nitrate anomalies (in panel **b**) and sulfide concentrations near the detection limit (in panel **c**) from 2–6 cm indicate active bio-irrigation. Bio-irrigation is likely also responsible for the elevated sulfide concentrations in the top centimeters of the sediment core, where reduced materials are excreted by Tubifex worms. Source data

**Extended Data Fig. 2:**
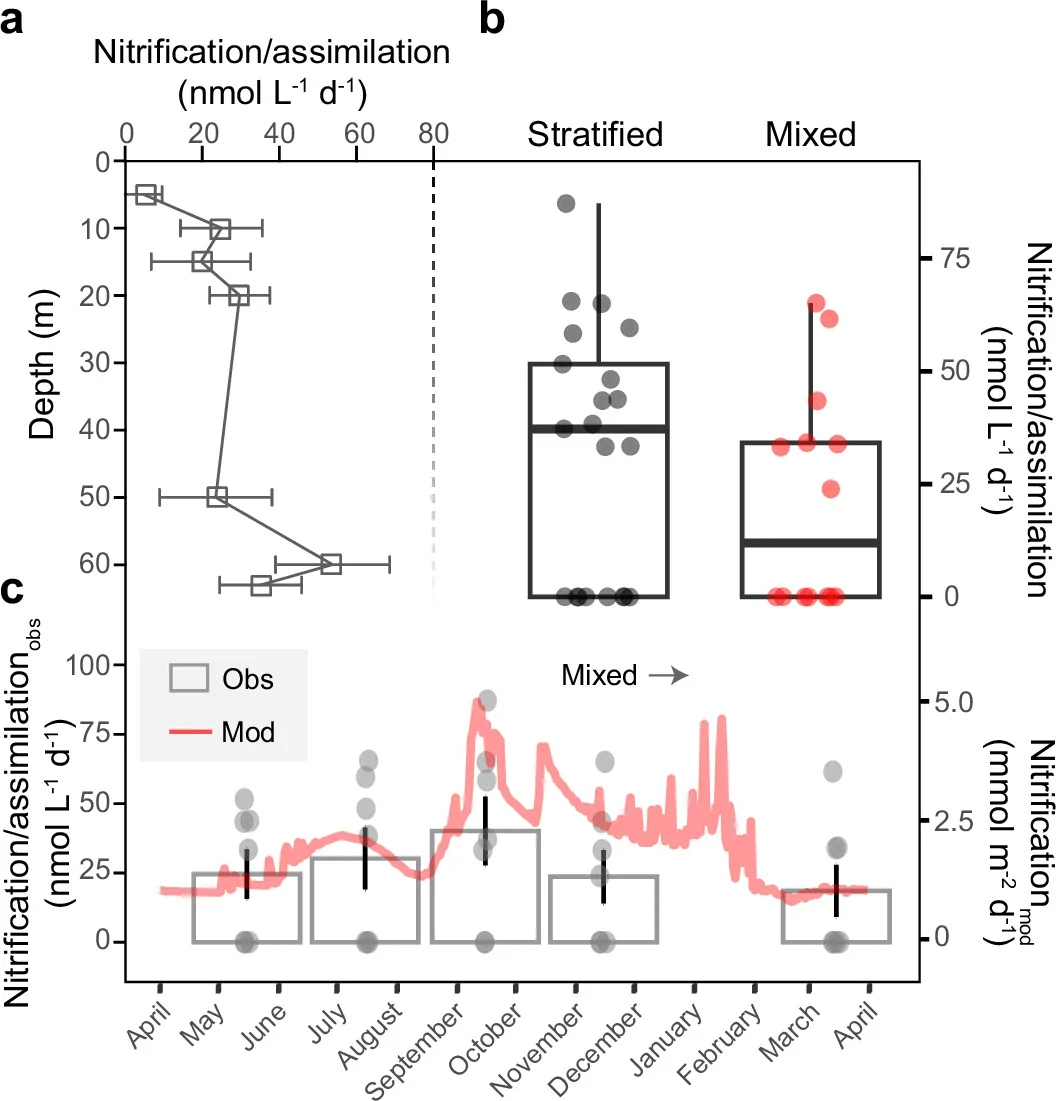
Water column rates of ammonium consumption associated with nitrification/assimilation processes in Baldeggersee. The consumption of ^15^NH_4_^+^ (in ^15^NH_4_^+^ additions) over time was used to determine nitrification/assimilation rates. **(a)** Vertical distribution of nitrification/assimilation rates. Data are presented as mean values +/− standard deviation (n = 5; representing seasonal monthly data). (**b**) Rates of nitrification/assimilation under contrasting hydrodynamic regimes. The mixed regime is defined by a ≤ 1 °C difference between the surface and bottom waters (see Methods). Values are grouped by pooling all depth measurements within each mixing regime. The lower and upper hinges of the boxplots represent the 25th and 75th percentiles, and the solid lines indicate medians. Whiskers extend to the most extreme values within 1.5 × the interquartile range, and points beyond the whiskers represent outliers. (**c**) Seasonal trends for nitrification/assimilation activity based on observations (bars; mean ± standard error, n = 7; replicates represent multiple depths). Modeled nitrification data (red line) is also shown. See Supplementary Table 1 for details on stations and sample collection dates. Source data

Our incubations with organic and inorganic ^15^N tracers find that potential nitrogen transformation activity is vertically distributed across a relatively large section of the upper sediments (Fig. 1b). Potential nitrogen turnover rates were not only elevated at the nitracline (∼0.25 cm), but activity extended much deeper down to ∼10 cm depth before declining. This pattern coincided with the vertical extent of the bioirrigated zone, as it has been shown that the presence of sediment fauna can facilitate the transport of nitrate below the chemoclines, sustaining denitrification, and can lead to the injection of biodeposits (faeces), fuelling heterotrophic bacteria in subsurface sediments^28,34–36^. High-resolution porewater profiles further show episodic nitrate anomalies at depth and an absence of free sulfide within the *Tubifex*-burrowed layer, consistent with oxidant transport by macrofauna spanning oxic–anoxic zones (Extended Data Fig. 1). Such fauna-driven nitrate supply helps explain the presence of denitrifiers such as *Methylotenera* (within the order Methylophilales^37,38^, discussed later) below the nitracline (Fig. 1c); their abundances correlate with depth-resolved potential denitrification rates (Pearson correlation, one-tailed *P* = 0.033; d.f. = 8; *α* = 0.05). In addition to nitrate, oxygen may also be delivered by fauna into ammonium-rich sediments, which could support the presence of some nitrifiers below the oxycline (Fig. 1b). However, the vast majority of nitrification activity occurs in the overlying water column (Extended Data Fig. 2)^39^, suggesting that water column nitrification and exogenous nitrate sources (for example, riverine input) are the primary contributors of nitrate fuelling sedimentary denitrification and DNRA.

**Extended Data Fig. 3:**
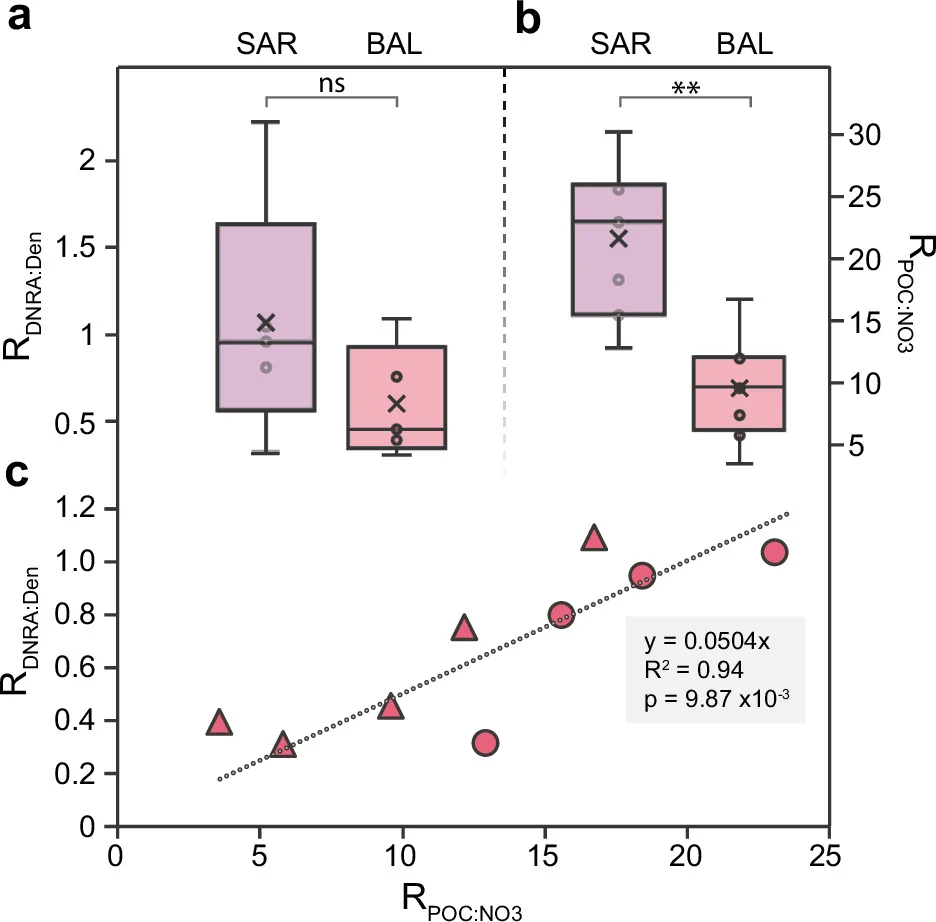
Relationship between R_DNRA:Den_ and R_POC:NO3_ in Lakes Baldeggersee (eutrophic) and Sarnersee (oligotrophic). (**a, b**) Whisker plots comparing R_DNRA:Den_ and R_POC:NO3_ between Baldeggersee and Sarnersee. For R_DNRA:Den_, the sample size for both lakes is n = 5 (representing seasonal monthly data). For R_POC:NO3_, the sample size is n = 8 for Baldeggersee and n = 7 for Sarnersee (representing seasonal monthly data). A Mann-Whitney test (one-tailed) was applied to evaluate the degree of variance between Lakes Baldeggersee and Sarnersee, with the asterisk(s) denoting the significance: *, p < 0.05; **, p < 0.01; ***, p « 0.01. For R_DNRA:Den_, the comparison between Baldeggersee and Sarnersee yielded p = 0.16, whereas for R_POC:NO3,_ the comparison yielded p = 0.01. The lower and upper hinges of the boxplots represent the 25th and 75th percentiles, crossed symbols denote means, and solid lines indicate medians. Whiskers extend from the median to 1.5 × the interquartile range, and data points beyond the whiskers represent outliers. **(c)** Correlation plot showing the observed relationship between R_DNRA:Den_ and R_POC:NO3_ for Baldeggersee (triangles) and Sarnersee (circles), based on monthly seasonal datasets from both lakes. Here, total POC flux is used (refocused plus euphotic-zone-derived components; Extended Data Fig. 4). A linear regression and t-test (two-tailed) were performed to assess the statistical significance (df = 7; p = 9.87 × 10^−3^, p < 0.01). Abbreviations: DNRA, dissimilatory nitrate reduction to ammonium; POC, particulate organic matter. Source data

Of the potential nitrogen transformation processes measured in the bioirrigated zone, denitrification was the most prevalent based on mean volumetric rates averaged over a seasonal cycle (Fig. 1b). Potential denitrification activity (0.77 nmol cm^−3^ h^−1^; Fig. 1b) far exceeded potential anammox rates by an order of magnitude (0.05 nmol cm^−3^ h^−1^). Denitrification also surpassed potential DNRA activity (0.50 nmol cm^−3^ h^−1^) by 1.6-fold, indicating that denitrifying bacteria outcompete both DNRA and anammox bacteria for available inorganic NO_*x*_. A whole-lake budget analysis likewise places denitrification above DNRA by 1.4-fold (Fig. 1a). Altogether, this aligns with established trends, which have shown that nitrate-rich (NO^3^^−^) conditions relative to organic carbon ($R_{\mathrm{OC}:\mathrm{NO}_3^-} \le 10:1$), such as those encountered in Baldeggersee, where the ratio of particulate organic carbon (POC) to nitrate was 10 ($R_{\mathrm{POC}:\mathrm{NO}_3^-}=10$), upregulate denitrification^10,33^. Conversely, a high $R_{\mathrm{POC}:\mathrm{NO}_3^-}$ (>10:1) would favour DNRA over denitrification, as observed in a neighbouring oligotrophic lake ($R_{\mathrm{POC}:\mathrm{NO}_3^-}=22$; Extended Data Fig. 3a,b).

### Influence of the mixed regime on nitrogen removal

**Fig. 2:**
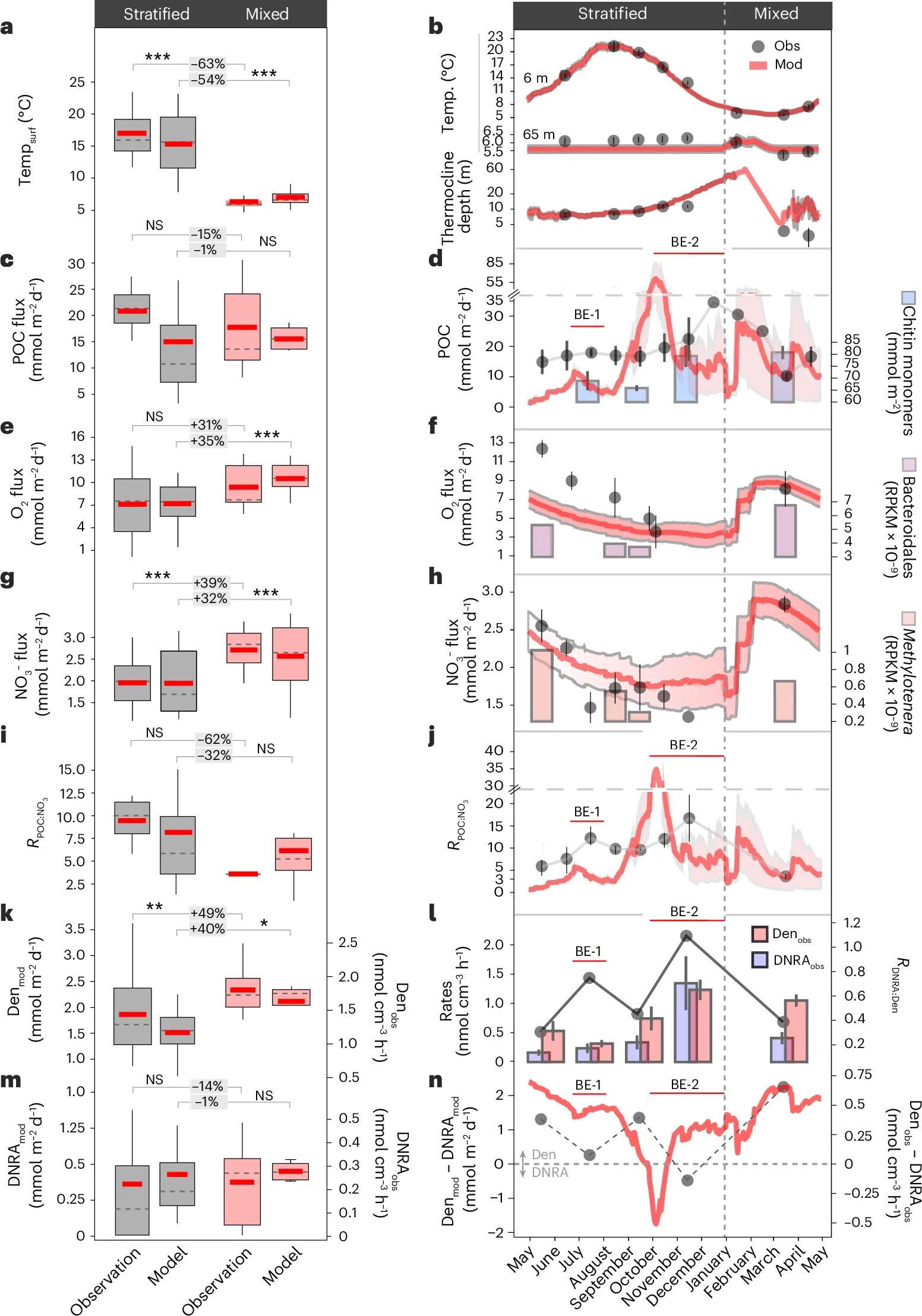
Seasonal dynamics of Baldeggersee physics, chemistry and nitrogen removal, along with associated key microbial players. **a**,**c**,**e**,**g**,**i**,**k**,**m**, Effects of the stratified and mixed regimes on observational and modelled lake parameters. We define the mixed regime as a 1 °C separation between top and bottom waters. In the box plot, the lower and upper hinges represent the 25th and 75th percentiles, solid red lines denote means and dotted lines indicate medians. Whiskers extend to the most extreme values within 1.5× the interquartile range. For model outputs, daily seasonal values (from panels on the right) were aggregated to monthly means and then grouped according to the mixing regime. Likewise, seasonal observational data (shown on panels on the right) were grouped by regime. The Mann–Whitney test (two tailed) was applied to evaluate differences between regimes with the asterisks denoting significance: *P < 0.05; **P < 0.01; ***P << 0.01, and non-significant differences denoted by ‘NS’ (observations, model, **a**: *P* = 5.56 × 10^−8^, 1.38 × 10^−15^; model, **e**: *P* = 3.47 × 10^−6^; observations, model, **g**: *P* = 4.11 × 10^−5^, 1.33 × 10^−3^; observations, model, **k**: *P* = 0.013, 0.030). Percentage differences between regimes (relative to the mixed regime) are shown above the panels. Sample sizes, *n* (model, observation) for mixed and stratified regimes were as follows: **a**, mixed (36, 17) and stratified (72, 25); **c**, mixed (24, 4) and stratified (48, 8); **e**, mixed (24, 5) and stratified (48, 25); **g**, mixed (24, 18) and stratified (48, 36); **i**, mixed (24, 1) and stratified (48, 6); **k**, mixed (24, 18) and stratified (48, 40); and **m**, mixed (24, 10) and stratified (48, 40). In **k** and **m**, potential denitrification and DNRA rates (observed data) were quantified from ^15^N stable-isotope addition experiments. Potential denitrification and DNRA rates are expressed as volumetric rates and are compared with the modelled aerial rates. **b**,**d**,**f**,**h**,**j**,**l**,**n**, Seasonal trends of observational and modelled data over the annual cycle. Modelled data are shown as means with upper and lower variability ranges. Observational data (including bars or points) are presented as mean values ± s.e.m. (with the means calculated spanning various seasons and stations; Supplementary Table 1). Bars in **d** are presented as depth integrated (0–2.5 cm) ± propagated uncertainty calculated from five samples. Bars in **l** show the mean ± s.e.m. calculated across ten depth intervals (Fig. 1b). The chitin inventory (based on concentrations of glucosamine and *N*-acetylglucosamine, *m*/*z* = 178 and 220, respectively) in the upper sediments is shown alongside the abundance of the chitinolytic–denitrifying consortium. Oxygen and nitrate fluxes (into the sediment) were derived from microrhizon porewater measurements. The observed sediment POC flux is based on the lower sediment trap and represents total POC (refocused plus euphotic-zone-derived fluxes; Extended Data Fig. 4); the modelled POC flux shown likewise represents total POC. Two bloom events (BE-1 and BE-2) associated with high POC sedimentation are indicated. Observational data related to oxygen, nitrate and POC were reported (data from ref. ^19^). Source data

When potential denitrification was assessed over a seasonal cycle in Baldeggersee, we found that mean volumetric activity begins at moderate rates (∼0.5 nmol cm^−3^ h^−1^; Fig. 2l) in spring, declines to a low point in mid-summer (∼0.3 nmol cm^−3^ h^−1^), then increases to 0.7–1.2 nmol cm^−3^ h^−1^ in late summer. The high rates measured in late summer persisted during the mixed regime (∼1.0 nmol cm^−3^ h^−1^). When grouped according to the mixing regime, potential denitrification was highest in the mixed period, exceeding activity in the stratified period by 49% (Fig. 2k). The enhanced denitrification during the mixed regime further corresponded with the greatest nitrate flux into the sediment, reaching up to 2.8 mmol m^−2^ d^−1^, which was 39% greater than that in the stratified regime (Fig. 2g,h).

While benthic denitrification activity was enhanced in the mixed period, the other competing process for nitrate, DNRA, showed no statistically significant difference between the mixing regimes in Baldeggersee (Fig. 2m). However, over a seasonal cycle, the ratio between the two nitrate-reducing processes (*R*_DNRA:Den_) varied systematically. Namely, we observed a greater prevalence of DNRA relative to denitrification (*R*_DNRA:Den_ > 0.8; Fig. 2l) during mid-summer and late-summer bloom events (BE-1 and BE-2) when POC fluxes to the sediment increased (Fig. 2d). This also coincided with when $R_{\mathrm{POC}:\mathrm{NO}_3^-}$ values—an indicator of the balance between DNRA and denitrification^10^—attained their highest values of 12 and 15 for BE-1 and BE-2, respectively (Fig. 2j). By contrast, denitrification prevailed over DNRA (*R*_DNRA:Den_ < 0.5; Fig. 2l) during the inter-bloom periods and in the mixed regime when the $R_{\mathrm{POC}:\mathrm{NO}_3^-}$ ratio fell below 10. A collation of data from Baldeggersee and an oligotrophic lake from the same region reveals a robust correlative seasonal trend (*ω*^obs^; equation (13)), between the ratios of $R_{\mathrm{POC}:\mathrm{NO}_3^-}$ and *R*_DNRA:Den_ (Extended Data Fig. 3c; *t*-test, two-tailed *P* = 0.01; d.f. = 7; *α* = 0.05). This reinforces the role that organic matter and nitrate availability have in determining whether fixed nitrogen is denitrified to dinitrogen gas or regenerated back to ammonium (via DNRA), in line with previous work^10^.

**Extended Data Fig. 4:**
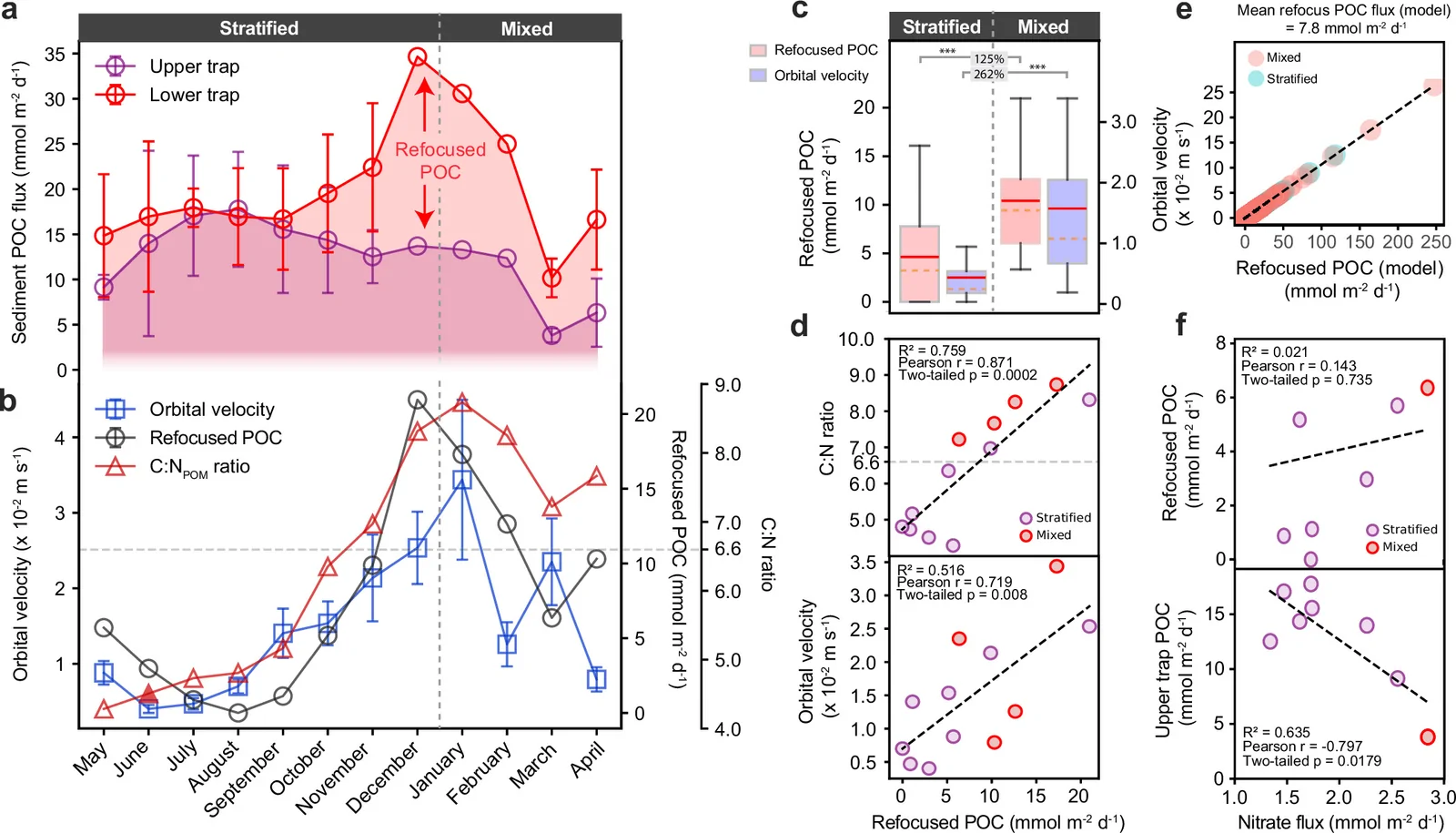
Observations of sediment refocusing and its coupling to wind-driven meteorological data in Baldeggersee. Panels (**a, b**) show seasonal variability in water-column POC flux derived from upper and lower sediment traps previously reported by Müller et al.^19^. (**a**) The upper trap was positioned 15 m below the surface and reflects euphotic-zone-derived POC export, whereas the lower trap, located 3 m above the sediment, represents total POC flux (refocused/lateral plus vertical export). Data represent mean values of aggregated bi-weekly sediment trap measurements collected between 2017 and 2018 (mean ± SD; Mar–Sep n = 4, Oct n = 3, Nov n = 2). (**b**) Refocused POC flux was calculated as the difference between lower and upper trap fluxes. Wind-driven near-bed orbital velocities derived from meteorological forcing, which are the same data used to force the GLM-AED model, are also shown (mean ± SD; n = 30, representing daily values aggregated into monthly means for 2017–2022). Seasonal changes in particulate organic matter C:N ratios are additionally shown, with the Redfield ratio (C:N = 6.6) indicated for reference. (**c**) Boxplot comparing refocused POC fluxes between stratified and mixed regimes. The mixed regime is defined as a temperature difference of ≤ 1 °C between surface and bottom waters (see Methods). Weekly measurements were aggregated by mixing regime (stratified or mixed). Sample sizes were: orbital velocity, stratified/mixed n = 34/18; refocused POC, stratified/mixed n = 29/9. The lower and upper hinges represent the 25th and 75th percentiles, solid lines denote means, and dotted lines indicate medians. Whiskers extend to the most extreme values within 1.5 × the interquartile range. Statistical differences between regimes were assessed using a two-tailed Mann–Whitney test. Significance levels are indicated as *p < 0.05, **p < 0.01, and ***p < 0.001 (refocused POC: p = 0.0097; orbital velocity: p = 1.9 × 10^−5^). (**d, f**) Correlation analyses between observed biogeochemical and physical parameters (monthly data shown); Pearson correlation coefficients (two-tailed) and p-values are reported (C:N ratio vs refocused POC: df = 10, p = 0.0002; orbital velocity vs refocused POC: df = 10, p = 0.008; refocused POC vs nitrate flux: df = 5, p = 0.735; upper-trap POC vs nitrate flux: df = 6, p = 0.0179). (**e**) Empirical calibration curve used in the model to relate refocused POC flux to wind-driven orbital velocities (based on meteorological data). Source data

**Extended Data Fig. 5:**
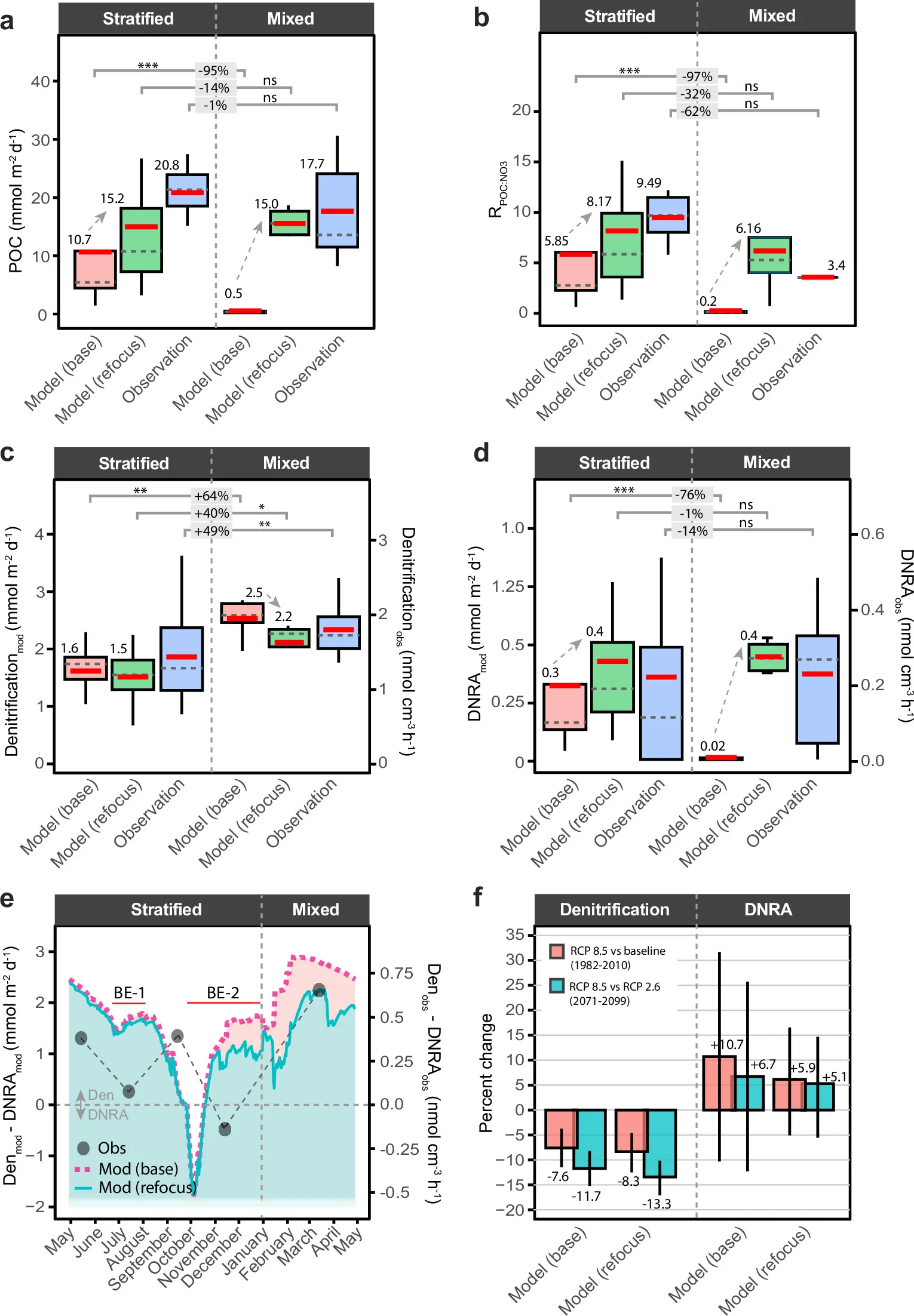
Improvement in model–observation agreement following the transition from the base to the refocused model for Baldeggersee. (**a-d**) Boxplots comparing the base model versus the refocused POC model and the observations for the respective stratified and mixed regimes. The mixed regime is defined by a temperature difference of ≤ 1 °C between surface and bottom waters (see Methods). Observational data, collected on a bi-monthly or bi-weekly basis during seasonal sampling campaigns, were aggregated according to mixing regime (stratified or mixed). Model outputs, originally produced at daily resolution, were first aggregated to monthly means and subsequently grouped by mixing regime. The lower and upper hinges of the boxplots represent the 25th and 75th percentiles, solid red lines denote means, and dotted lines indicate medians. Whiskers extend to the most extreme values within 1.5 × the interquartile range. Statistical differences between regimes were evaluated using a two-tailed Mann–Whitney test. Significance levels are indicated as *p < 0.05, **p < 0.01, and ***p < 0.001; non-significant differences are denoted as ‘ns’ (model base POC, panel **a**: p = 0.0043; model base R_POC:NO3_, panel **b**: p = 3.11 × 10^−3^; model base, model refocused, and observed denitrification, panel **c**: p = 3.11 × 10^−3^, 0.030, 0.013; model base DNRA, panel **d**: p = 1.56 × 10^−3^). Mixed-regime observations with limited sample size (n ≤ 10) include POC (n = 7), and R_POC:NO3_ (n = 1); all other parameters have n > 10. (**e**) Seasonal variability of sediment denitrification and DNRA activity. (**f**) Comparison of the base and refocused POC model configurations in projections of Baldeggersee’s future capacity to remove fixed nitrogen. Data are presented as mean values +/− SEM (n = 29; replicates represent aggregated annual simulations). Source data

The relationship between $R_{\mathrm{POC}:\mathrm{NO}_3^-}$ and *R*_DNRA:Den_ from observations was then further leveraged to predict the seasonal dynamics of nitrate-reducing processes using a biogeochemical model simulating Baldeggersee. First, the model was tuned to mimic the lake’s thermal state during the two mixing regimes (Fig. 2a,b). Next, we simulated the nitrate flux and the particulate organic matter (POC) flux to the sediment (Fig. 2c,d,g,h). The model captured the pronounced seasonality in nitrate supply, while the POC flux showed a weaker seasonal signal, reflecting the combined influence of vertical export from surface waters and lateral POC transport (Extended Data Figs. 4 and 5). These fluxes, in turn, define the seasonal dynamics of $R_{\mathrm{POC}:\mathrm{NO}_3^-}$ in the model (Fig. 2i,j). Comparison with observations showed that the model reproduced both the high $R_{\mathrm{POC}:\mathrm{NO}_3^-}$ values during stratification, such as those associated with the mid-summer (BE-1) and late-autumn (BE-2) bloom periods, and the lower values characteristic of the mixed regime.

On the basis of the modelled $R_{\mathrm{POC}:\mathrm{NO}_3^-}$ values, we were able to quantify the seasonal activities of DNRA and denitrification using an approach that estimates the relative partitioning of nitrate reduction based on the relationship between *R*_DNRA:Den_ and $R_{\mathrm{POC}:\mathrm{NO}_3^-}$ and the derived proportionality coefficient (*ω*^obs^ = 0.0504; Extended Data Fig. 3c; equations (13)–(15)). The resulting modelled denitrification activity exceeded DNRA throughout most of the year, consistent with observations (Fig. 2n). In the stratified period, modelled denitrification experienced episodic lows in summer (BE-1) and late autumn (BE-2) reciprocated by periodic highs in DNRA. These modelled patterns align with observations, although with a slight temporal offset. As the system transitioned from stratified to mixed conditions, modelled denitrification activity peaked, comparing well with the observations. Overall, both modelling and observations assert that the mixed regime accounts for between 40% and 49% more fixed nitrogen removal per day than the stratified regime (Fig. 2k), underscoring its role in the lake nitrogen budget.

### Powering nitrogen removal rates in the mixed regime

**Extended Data Fig. 6:**
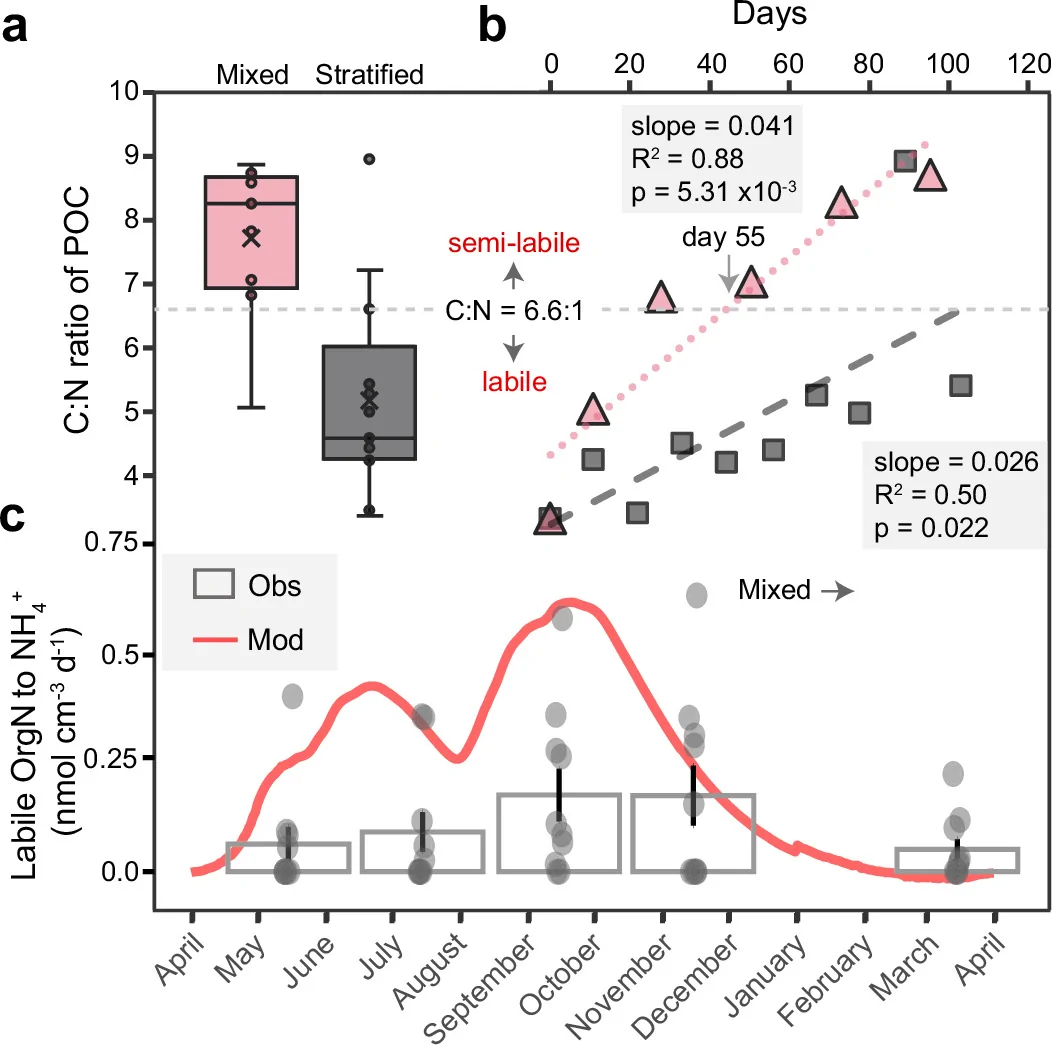
Organic matter composition and its turnover in Baldeggersee. **(a, b)** Elemental C:N ratios of the settled particulate organic matter (POC). (**a**) The sample size for the mixed and stratified periods is n = 9 and n = 13 (representing seasonal monthly data), respectively. The lower and upper hinges of the boxplots represent the 25th and 75th percentiles, crossed symbols denote means, and solid lines indicate medians. Whiskers extend to the most extreme values within 1.5 × the interquartile range, and points beyond the whiskers represent outliers. (**b**) Shows the change in the C:N ratios of collected sediment trap material during the mixed (triangle) and stratified (square) periods, respectively. The water column sediment trap was positioned at the deepest point in the lake (red star in Fig. 1a) at 3 meters above the sediment. Note that in the mixed regime, the material collected in the trap is likely derived from sediment resuspension, providing useful insight into the changing composition of surface sediments deposited over this period. The Redfield C:N stoichiometry of 6.6 to 1 (grey dotted line) is used as a reference to indicate the reactivity of organic matter in Baldeggersee. During the winter mixed regime, which lasts 121 days in total, the C:N ratio surpasses the Redfield ratio at day 55. A linear regression is shown, and a two-tailed *t-test* was applied to determine statistical significance (mixed regime: df = 4, p = 5.31 × 10^−3^, p < 0.01; stratified regime: df = 8, p = 0.022, p < 0.05). (**c**) Seasonal rates of labile organic nitrogen remineralization to NH_4_^+^ (nmol cm^−3^ h^−1^) are shown for observations (bars; mean ± SEM, n = 10, aggregated sediment depth intervals), with modeled output overlaid (red line) to enable seasonal comparisons. We determined labile organic N remineralization rates by measuring the turnover of fresh ^15^N-labelled algal biomass (*Spirulina platensi)* in slurry addition experiments, specifically monitoring the production of ^15^NH_4_^+^. See Supplementary Table 1 for details on stations and sample collection dates. Source data

As previously highlighted^13,19^, enhanced N removal in the mixed regime of Baldeggersee was correlated with an increased oxygen flux into the sediment (Fig. 2f). Enhanced oxygen availability through water column mixing was hypothesized to accelerate sedimentary organic N mineralization, liberating ammonium for nitrification and subsequent denitrification at the water–sediment interface (Fig. 1b,d). This interaction can be exacerbated as mixing resuspends settled organic matter detritus, re-exposing organic nitrogen to water column oxygen^19^. Large summer deposits of labile and semi-labile organic nitrogen, which can constitute 21–29% and 10–20% of sediment material, respectively^40,41^, are particularly affected by this increased oxygen exposure. In Baldeggersee, the re-ventilation during the mixed regime accelerated organic nitrogen remineralization by as much as 58% relative to the stratified period (Extended Data Fig. 6b). Within the mixed regime, labile organic nitrogen (characterized by a C:N ratio of less than 6.6:1) was turned over in the first third of the period, leaving behind semi-labile material. Semi-labile compounds such as chitin—an abundant aminopolysaccharide in sediments—are thought to play a key role in microbial remineralization, serving as important substrates for ammonium release that can fuel nitrification and subsequent denitrification at the water–sediment interface^39,40,42^. In our study, we observed that chitin (probably deposited during the summer phytoplankton bloom) was indeed prevalent during the mixed regime in Baldeggersee (Fig. 2d), a period when the oxygen flux into the sediment was also at its peak. Oxygen availability is critical for microbial chitin degradation, as the compound tends to be preserved under anoxic conditions^40,43,44^. Notably, the aerobic breakdown of chitin monomers (*N*-acetylglucosamine; equation (1)) requires 120% more molecular oxygen than the respiration of labile amino acids (equation (2)), yet it generates 207% more energy for growth, as reflected by the more negative standard Gibbs free energy change (Δ*G°*) values.

1 $${{\rm{C}}}_{8}{{\rm{H}}}_{15}{\mathrm{NO}}_{6}+8.25{{\rm{O}}}_{2}\to 8{\mathrm{CO}}_{2}+6.5{{\rm{H}}}_{2}{\rm{O}}+{\mathrm{NH}}_{3}\,\Delta G^\circ =-3,639\,\mathrm{kJ}\,{\mathrm{mol}}^{-1}$$

2 $${{\rm{C}}}_{3}{{\rm{H}}}_{7}{\mathrm{NO}}_{2}+3.75{{\rm{O}}}_{2}\to 3{\mathrm{CO}}_{2}+2{{\rm{H}}}_{2}{\rm{O}}+{\mathrm{NH}}_{3}\,\Delta G^\circ =-1,187\,\mathrm{kJ}\,{\mathrm{mol}}^{-1}$$

**Fig. 3:**
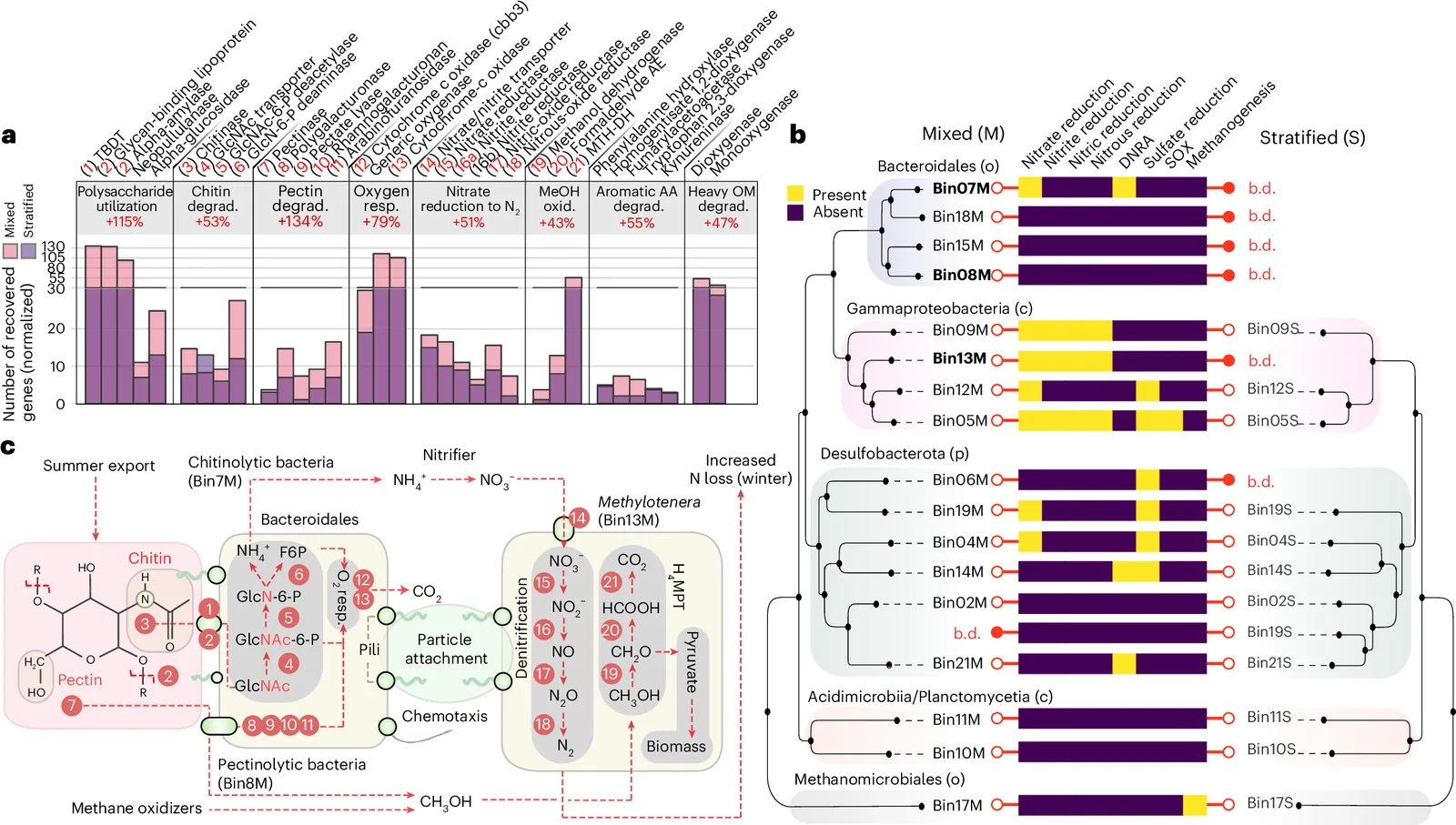
Putative microbial consortium driving denitrification in the mixed regime. **a**, Metagenomic analysis of Baldeggersee sediments focusing on key genes involved in chitin and pectin degradation, including the downstream pathways of oxygen respiration, denitrification and methanol oxidation. Gene abundances are expressed as relative abundances, normalized to total raw reads per sample. Values in red indicate the percentage increase in the respective gene cluster from stratified to mixed conditions based on total gene counts. **b**, Phylogenetic analysis and functional potential of MAGs recovered under mixed and stratified conditions in Baldeggersee sediments. Rows are ordered by a maximum likelihood tree built from 49 universal single-copy genes. For each regime, an open red circle indicates a MAG detected and passing quality controls; ‘b.d.’ (red) is indicated if a MAG failed at least one of the following quality control criteria (completion below 50% and/or contamination above 5). When mixed and stratified bins represent the same species (average nucleotide identity (ANI) ≥ 95%), they are paired on the same row. Bars show presence (yellow) and absence (purple) of key pathways (nitrate reduction, nitrite reduction, nitric oxide reduction, DNRA, sulfate reduction, sulfide oxidation (SOX), methanogenesis) for the representative MAG. We recovered 17 MAGs in mixed and 12 under stratified conditions; 11 species were shared across both regimes, while 6 MAGs were unique to the mixed regime, and 1 was unique to the stratified regime. See also Supplementary Table 2 for information concerning the relative abundances of key MAGs. Bins indicated in bold are discussed in the text. **c**, Illustration of genes and pathways related to the chitinolytic or pectinolytic–denitrifying consortium. Numbers 1 to 21 correspond to the genes shown in the metagenome analysis in **a**. Attachment to the particle involves encoded type IV pili in both *Methylotenera* and Bacteroidales. Bacteroidales MAGs also encoded flagella, suggesting that they could alternate between a free-living and particle-attached lifestyle. In *Methylotenera*, formaldehyde assimilation into biomass occurs via the ribulose monophosphate pathway (RuMP cycle), whereas formaldehyde oxidation is mediated by the tetrahydromethanopterin pathway (H_4_MPT). GlcNAc, *N*-acetyl-D-glucosamine; GlcNAc-6-P, *N*-acetylglucosamine-6-phosphate; GlcN-6-P, glucosamine-6-phosphate; MTH-DH, methylenetetrahydrofolate dehydrogenase; F6P, fructose-6-phosphate. Source data

Our functional gene analysis of sediment metagenomes collected from Baldeggersee revealed that microbial pathways for chitin degradation and oxygen respiration were markedly enriched in the mixed regime (>50%) compared with the stratified regime (Fig. 3a). Specifically, this included both the enzymes responsible for liberating ammonium from chitin (glucosamine-6-phosphate deaminase), as well as the high-affinity cytochrome c oxidase (cbb3-type), which enables respiration under microaerobic conditions. Concurrently, we recovered several polysaccharide-degrading oxygen-respiring bacterial metagenomes within the order of Bacteroidales (Fig. 3b and Supplementary Figs. 1 and 2), which were 49–131% more abundant in the mixed regime than in the stratified regime (Supplementary Table 2). Assessing the vertical distribution of Bacteroidales indicates that they are most abundant in the top 2 cm of the sediment, where chitin was also prevalent (Fig. 1c). The requisite oxygen needed to support chitin degradation by Bacteroidales is probably sustained via passive oxygen diffusion combined with bioirrigation. Along these lines, Bacteroidales sequences have been found in close association with macrofauna burrows and their larvae^45^.

**Extended Data Fig. 7:**
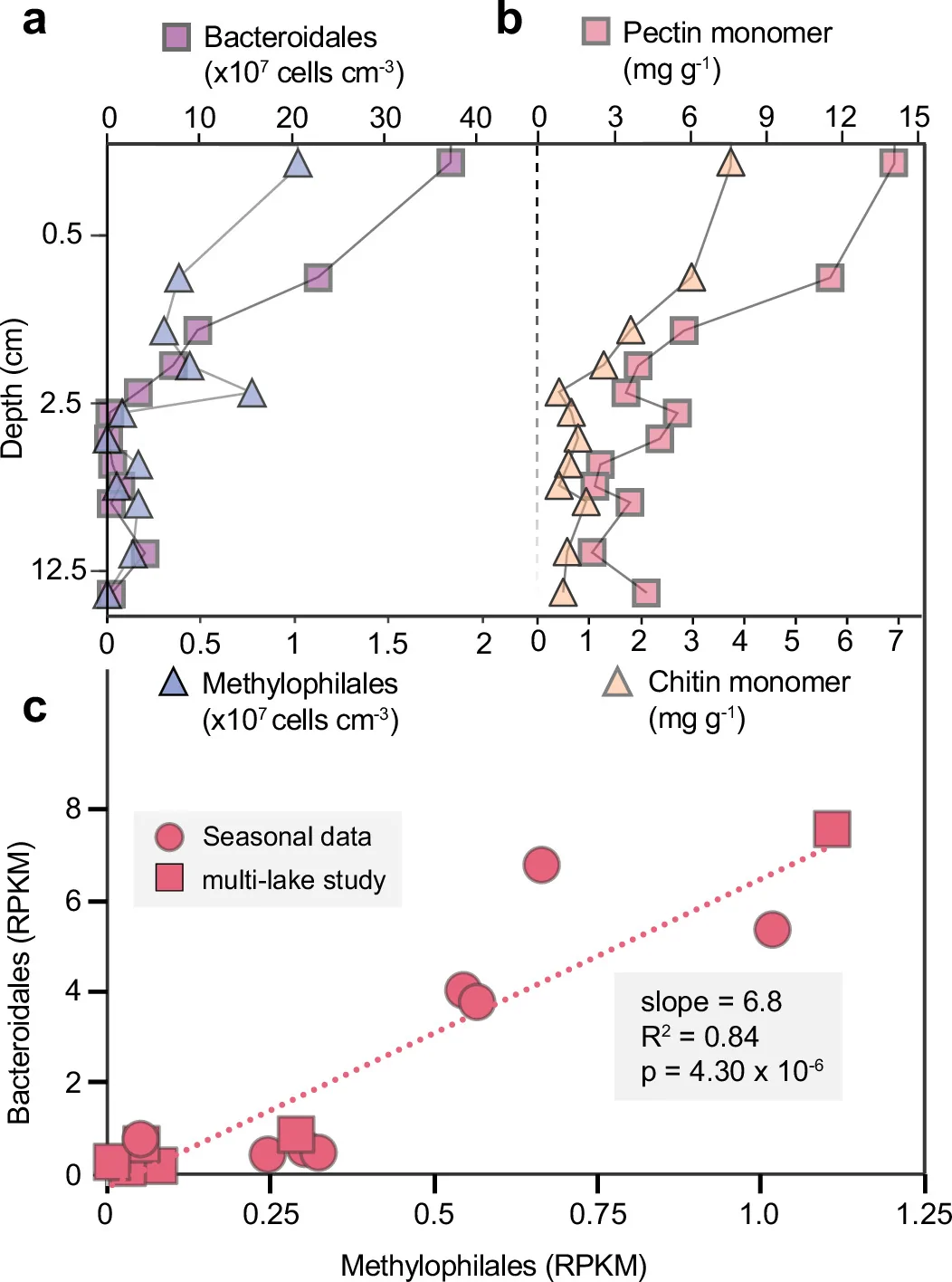
Vertical and temporal relationship between Bacteroidales (Bins 7, 8, 15, 18 M) and Methylophilales (Bin13M). (**a**) Vertical abundances of Bacteroidales and Methylophilales were enumerated using CARD-FISH (see Methods). (**b**) Concentration profiles of monomers related to pectin (galacturonic acid m/z = 193) and chitin (glucosamine and N-acetyl-glucosamine m/z = 178 and 220, respectively). (**c**) Correlation between Bacteroidales (Bins 7, 8, 15, 18 M) and Methylophilales (Bin13M; Fig. 3b). Seasonal data refer to samples collected from 2017 to 2018 in Baldeggersee (Supplementary Table 1). Additionally, data from other lakes covering different trophic states are included in the correlation analysis, referred to as the ‘multi-lake study’. A linear regression is shown, and a two-tailed *t-test* was applied to assess statistical significance (df = 12; p = 4.30 × 10^−6^, p < 0.01). Source data

Emerging alongside polysaccharide-degrading bacteria in the mixed regime was the denitrifying genus *Methylotenera* (Fig. 3b: Bin13M). The recovered metagenome of *Methylotenera* (Bin13M) carried the capacity for nitrate reduction to dinitrogen gas (using the *nar*, *nir*, *nor* and *nos* genes), along with an ability to oxidize and assimilate methanol for energy and growth (Fig. 3, Supplementary Fig. 3 and Supplementary Table 2), akin to other studied members^46^. Methanol can be generated as a by-product of methane oxidation^47^ and/or during the degradation of polysaccharides such as pectin^48^. Indeed, some of the recovered metagenome-assembled genomes (MAGs) of Bacteroidales harboured an ability to generate methanol from pectin (for example, Bin08M; Supplementary Fig. 2). The predicted genomes thus indicate a potential synergistic relationship of *Methylotenera* and some Bacteroidales. In agreement with this hypothesis, cell enumeration of Bacteroidales and Methylophilales (an order that comprises *Methylotenera*; Supplementary Table 4) finds that they co-occur in the chitin- and pectin-rich upper sediments (Fig. 1c and Extended Data Fig. 7a,b). Although such co-occurrence could reflect habitat filtering at oxic–anoxic interfaces (that is, parallel responses to shared drivers rather than obligate coupling), the two groups also showed a temporal coupling: over a seasonal cycle, *Methylotenera* is positively correlated with pectinolytic–chitinolytic Bacteroidales (Extended Data Fig. 7c; Pearson correlation, two-tailed *P* < 0.01; d.f. = 12; *α* = 0.05), consistent with a close metabolic partnership. The proposed Bacteroidales–*Methylotenera* consortium reached its highest abundance in the late-mixed–early spring period, coinciding with the peak in chitin inventories and the fluxes of oxygen and nitrate into the sediments (Fig. 2d,f,h). The alignment of these variables suggests that the Bacteroidales–*Methylotenera* (or chitinolytic–denitrifying) consortium, indirectly abetted by oxygen, is probably a key contributor of nitrogen removal in the mixed regime (Fig. 3c). Moreover, the enrichment of Bacteroidales–*Methylotenera* during this period implicates polysaccharides and aminopolysaccharides as substrates powering denitrification.

### Sensitivity of nitrogen removal to increasing water column stratification

**Fig. 4:**
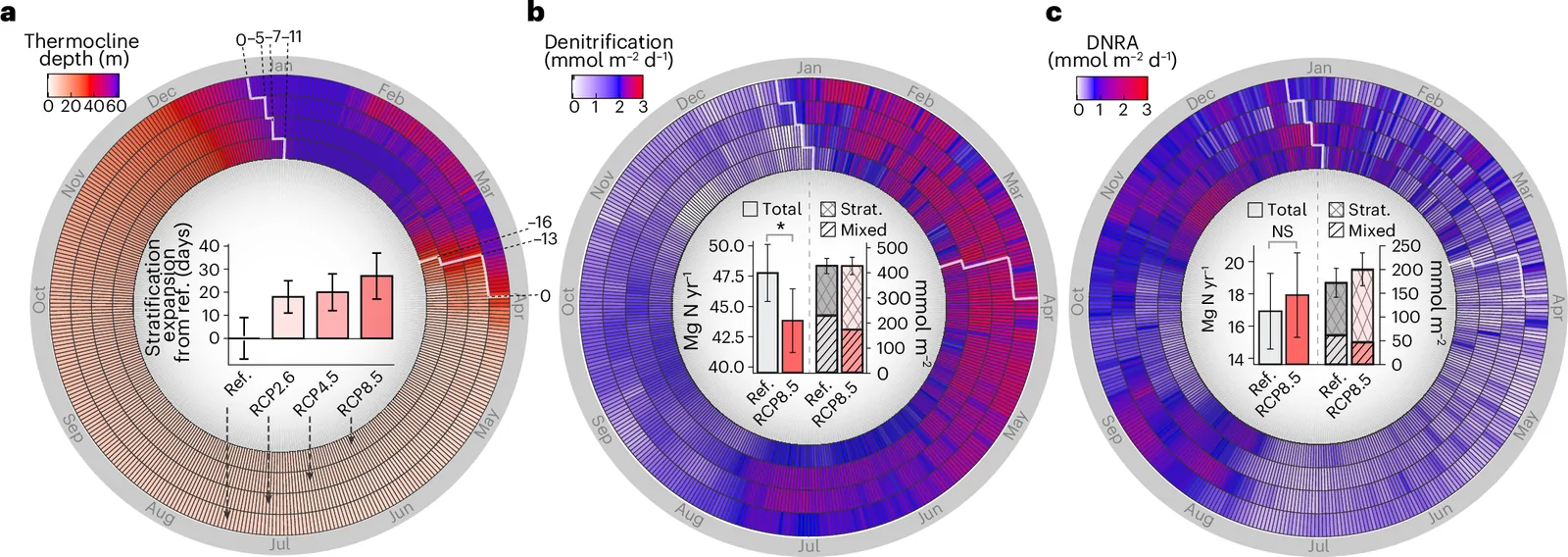
Future RCP scenario modelling of Baldeggersee’s physics and nitrogen removal. In all panels, the calculated time interval for the reference (Ref.) period (outermost ring) is from 1982 to 2010. Future projections for RCP 2.6 (low emission), RCP 4.5 (medium emission) and RCP 8.5 (high emission) warming scenarios are based on time intervals from 2071 to 2099, according to the Swiss Climate Change Scenarios CH2018 conventions. **a**, Projected phenology of the thermocline depth under various climate scenarios. For context, winter mixing induces a deepening of the thermocline, which normally stabilizes at 7 m depth in summer, indicating stratification. White lines (in all panels) denote the beginning and end of the mixed regime, as defined by a 1 °C separation between the top and bottom waters. Changes in the onset and termination of mixing, relative to the reference period, are indicated. The inner plot indicates the total duration of the stratified regime for the different climate scenarios (bars; mean ± s.e.m., *n* = 29; replicates represent aggregated annual simulations). **b**,**c**, Projected phenology of denitrification (**b**) and DNRA (**c**) under various climate scenarios. Rates were determined using modelled $R_{\mathrm{POC}:\mathrm{NO}_3^-}$ values and the established in situ correlation between *R*_DNRA:Den_ and $R_{\mathrm{POC}:\mathrm{NO}_3^-}$ (Extended Data Fig. 3c). Inner bar plots indicate the amount of N removed (via denitrification) or regenerated (via DNRA). Data are presented as mean values ± s.e.m. (*n* = 29; replicates represent aggregated annual simulations). The primary axis shows the total amount of nitrogen transformed by denitrification and DNRA, scaled to Baldeggersee’s surface area (5.22 × 10^6^ m^2^). A Welch *t*-test (two tailed) was applied to evaluate the degree of variance between RCP 8.5 and the reference period, with the asterisk(s) denoting significance: *P < 0.05; **P < 0.01; ***P ˂˂ 0.01 (denitrification: d.f. = 55.7, *P* = 0.032, *P* < 0.05; DNRA: d.f. = 55.7, *P* = 0.579, *P* > 0.05). The secondary axis of the inner plots shows the amount of nitrogen removed (via denitrification) or regenerated (via DNRA) under mixed and stratified (Strat.) conditions (bars; mean ± SEM, *n* = 29; replicates represent aggregated annual simulations). Source data

With average air temperatures expected to rise globally by 0.4 °C per decade under the worst-case climate scenario (RCP 8.5), warming is predicted to impact lake hydrodynamics, with feedback effects on the various biogeochemical cycles and associated microorganisms. Under a worst-case scenario, we find that heating Baldeggersee’s surface waters induces earlier thermocline stabilization (that is, stratification) in spring and a more subdued transition to the mixed regime in late autumn–early winter (Fig. 4a). Overall, this manifested in a 27-day reduction in the length of the mixed regime relative to the baseline, consistent with RCP 8.5 scenarios modelled for other lakes^14–17,49^.

**Extended Data Fig. 8:**
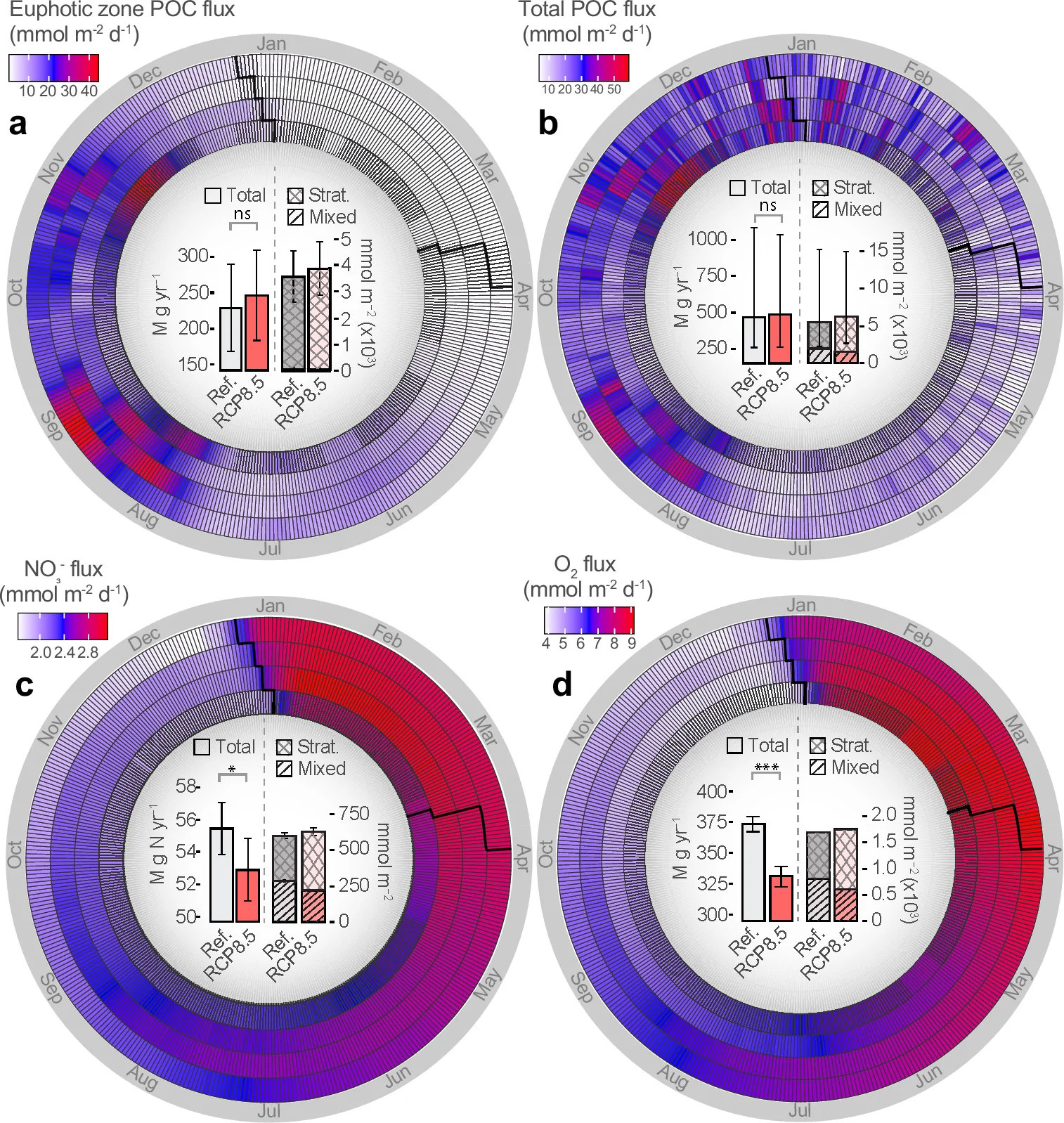
Future RCP scenario modeling of Baldeggersee biogeochemical fluxes. **(a, b, c, d)** The baseline (outermost ring), RCP 2.6, RCP 4.5, and RCP 8.5 (innermost ring) scenarios are indicated. The black lines (across all panels) denote the beginning and end of the mixed regime (constrained by a temperature difference ≤1 °C between surface and bottom waters; see Methods). Regarding the inner bar plots, data are presented as mean values +/− SEM (n = 29; replicates represent aggregated annual simulations). The primary axis shows the total amount of the flux, scaled to Baldeggersee’s surface area (5.22 × 10^6^ m^2^). A Welch test (one-tailed) was applied to evaluate the degree of variance between RCP 8.5 and the reference period, with the asterisk(s) denoting the significance: *, p < 0.05; **, p < 0.01; ***, p « 0.01, while non-significant differences are denoted ‘ns’ (NO_3_^−^ flux: df = 55.1, p = 0.026, p < 0.05; O_2_ flux: df = 53.2, p = 5.54 × 10^−11^, p < 0.01). The secondary axis in the inner plots shows total fluxes partitioned between mixed and stratified conditions. Panel **a** presents the euphotic-zone-derived POC flux, whereas panel **b** shows total POC flux (refocused plus euphotic-zone-derived fluxes; see also Extended Data Fig. 5). Source data

The contraction of the mixed regime alongside an expanding stratified period enables strong thermal density gradients to persist for longer in summer, creating a temporally extended niche for phytoplankton growth^50^. Under the worst-case scenario, this leads to a 7.7% increase in the euphotic-zone-exported POC flux to the sediment during the stratified period (Extended Data Fig. 8a), increasing the benthic oxygen demand and ultimately exacerbating sediment anoxia under a worst-case scenario. At the same time, we observed oxygen and nitrate fluxes into the sediments decline by 4.6–5.8% (Extended Data Fig. 8c,d), which has been previously predicted by biogeochemical models^51–53^.

We further find that under a worst-case scenario, benthic denitrification activity in Baldeggersee is projected to decrease by 61–103 mmol m^−2^ yr^−1^, corresponding to an 8.3–13.3% reduction relative to the baseline and the low-emission RCP 2.6 scenario (Fig. 4b; Mann–Whitney, *P* < 0.05; d.f. = 29; *α* = 0.05). In parallel, the attenuation of denitrification is partly substituted by an increase in DNRA by 8–9 mmol m^−2^ yr^−1^ (or by +5.1% to 5.9%) under a worst-case scenario (Fig. 4c).

## Discussion

Here we integrated ^15^N process rate measurements, flux estimates and molecular data to develop a comprehensive seasonal perspective on microbial denitrification in Baldeggersee. Our results show that the mixed period has a disproportionate role in removing fixed nitrogen, with denitrification largely governed by the balance between organic carbon and nitrate availability. Moreover, we identify a possible new microbial consortium, involving a chitinolytic–denitrifying coupling between Bacteriodales and *Methylotenera*, that points to polysaccharide degradation, particularly of chitin, as a potential metabolic underpinning of enhanced denitrification during the mixed regime. Chitin, derived mostly from microalgae and zooplankton, accounts for 7% of organic carbon in anoxically preserved sediments^40,42^. Given the average summer export rates from the euphotic zone of Baldeggersee of 18.3 mmol POC m^−2^ d^−1^ (ref. ^19^), the estimated chitin flux to the sediments is ∼1.1 mmol m^−2^ d^−1^. If preserved under summer anoxia, this could accumulate to 207 mmol m^−2^ by the onset of winter mixing—sufficient to sustain denitrification for ∼74 days (based on the measured nitrate flux), or ∼61% of the winter mixed period.

Leveraging these insights enabled a more accurate representation of the seasonal drivers of microbial denitrification within a modelling framework. Extending the calibrated model of Baldeggersee under future climate projections (across low- and high-emission RCP scenarios) indicates that microbial denitrification will decline substantially under a worst-case climate scenario relative to the baseline period (by 8.3–13.3%). This reduction arises from a shortening of the winter mixed regime, which disproportionately supports denitrification. At the same time, the expansion of the stratified period enlarges the phytoplankton niche, enhancing dissolved inorganic nitrogen assimilation in the water column and increasing organic matter burial (discussed below). These processes diminish nitrate resupply to the sediments at the onset of mixing, further constraining sedimentary denitrification. Lower nitrate availability also shifts the $R_{\mathrm{POC}:\mathrm{NO}_3^-}$ ratio slightly more in favour of DNRA, promoting fixed nitrogen recycling rather than removal. Ultimately, an increase in DNRA, together with a significant decrease in denitrification, manifests in more fixed nitrogen being retained and thus exported via the river outflow to downstream waterscapes under a worst-case climate scenario.

The reduction in denitrification projected under the worst-case scenario may be partly offset by increased burial of organic nitrogen associated with expanding anoxia. Macromolecules such as chitin, which are resistant to degradation in the absence of oxygen, can serve as long-term nitrogen sinks on geological timescales^40,43^. With the projected increase in the euphotic-zone-exported POC flux under a worst-case scenario (Extended Data Fig. 6a), we estimate higher export rates of chitin to the sediments, amounting to 20 mmol m^−2^ yr^−1^. Furthermore, the narrowing of the mixed regime reduces sediment oxygen fluxes, lowering the quantity of oxygen available for chitin respiration by 144 mmol m^−2^ yr^−1^ (Extended Data Fig. 8c). In turn, the lower oxygen availability translates to the preservation of 17 mmol m^−2^ yr^−1^ of N in the form of chitin, according to the stoichiometry in equation (1). Combined, the increased preservation of chitin (due to reduced oxygen availability), coupled with higher chitin export rates, amounts to a total chitin burial of 37 mmol N m^−2^ yr^−1^. The net effect of a worst-case scenario is that while denitrification activity is lowered by 61–103 mmol m^−2^ yr^−1^, increased N burial partially enhances N removal, resulting in a smaller net reduction of denitrification activity by 5.2–5.8% from the baseline. Additional factors, such as temperature-driven increases in enzymatic denitrification rates^13^, may further offset the loss of denitrification activity under a worst-case scenario.

Hence, multiple known (and possibly unknown) feedback effects could counteract the loss of Baldeggersee microbial denitrification. As a point of optimism, the microbial nitrogen cycle may show broad resilience to climate-induced alterations in the seasonality of stratification. Nevertheless, subtle shifts in stratification timing are still predicted to negatively impact nitrogen removal by denitrification while positively affecting nitrogen regeneration by DNRA in Baldeggersee, resulting in the release of 1–5 Mg N yr^−1^ to downstream ecosystems under a worst-case climate scenario. Other seasonally mixed lakes, which are analogously vulnerable to increasing stratification in the future^14^, may also undergo similar changes to their nitrogen removal efficacy. The resulting increase in fixed nitrogen export from lakes to downstream marine ecosystems could promote larger coastal phytoplankton blooms, potentially intensifying coastal eutrophication and contributing to the expansion of low-oxygen ‘dead zones’^54^.

## Methods

### Sampling overview

Five stations were sampled in Baldeggersee, ranging in depth from the deepest point to the shallowest point of the lake, as described in Supplementary Table 1. Various samples were collected and analysed from 2017 to 2018, including nutrients (nitrate and ammonium), oxygen and POC (collected in sediment traps). These samples were used to determine the fluxes of nitrate, ammonium and POC^19^. The regional Swiss Canton provided temperature profiles from 2017 to 2022 to help constrain the mixing regimes for model validation. In addition, samples for metagenomic analysis were collected and sequenced in a previous study^31^. In this study, we also report nitrogen transformation rates from ^15^N-stable isotope addition experiments, along with MAGs, cell enumerations (catalysed reporter deposition fluorescence in situ hybridization (CARD-FISH)) and sediment aminosugar concentrations. Water temperatures, nutrient chemistry, oxygen, POC fluxes and rate measurements were used to validate a biogeochemical model.

### Quantifying sediment nitrate-reducing processes

To provide a seasonal snapshot of activity, we quantified potential, or the upper bounds of microbial activity, using short-term ^15^N slurry incubations initiated immediately after sediment collection to minimize bottle effects and prevent shifts in community composition or enzyme expression. Briefly, a gravity core was used to collect sediment at the deepest point of Baldeggersee (Supplementary Table 1). Cores were transported to the laboratory and stored at 7 °C until further post-processing, which occurred roughly 3 h after collection. Sediment sections were extracted at 0–15 cm depth, using a water-driven piston extractor. Eight discrete depths were collected in the upper sediment at 0–8 cm (at 1-cm intervals), along with two deeper sediment intervals taken at 9–10 cm and 14–15 cm. After homogenization of the collected sediment sections, 1 g of sediment was transferred into 12-ml exetainers (Labco). The exetainers were then filled with filter-sterilized bottom waters (in a 1-l Duran bottle), which included ambient bottom water NO_3_^−^, as well as added O_2_ (50 µM, simulating bioirrigated sediments; Supplementary Discussion and Extended Data Fig. 1) and ^15^N-labelled tracers as follows: experiment 1 (Exp1), ^15^NO_3_^−^, and experiment 2 (Exp2), ^15^NH_4_^+^ + ^14^NO_2_^−^. Exp1 was used for the determination of DNRA and denitrification activity, whereas Exp2 was used for the quantification of anammox. In the experiments, the ^15^N labelling percentage for Baldeggersee ranged between 19% and 24% for ^15^NO_3_^−^ and 23% and 50% for ^15^NH_4_^+^. After the exetainers were capped, they were incubated in the dark and at in situ temperatures (7 °C), on a horizontal shaker (120 rpm). Although end-over-end rotation is the traditional slurry approach, we used gentle horizontal shaking to maintain solute exchange (O_2_/NO_3_^−^) while allowing particles to settle, thereby avoiding continuous high-shear resuspension that can disrupt microorganisms and their aggregates^55^. Experiments were terminated at designated time points (0 h, 6 h, 12 h, 24 h and 36 h) by first adding a 2-ml helium headspace, followed by the addition of 100 µl of ZnCl (50% w/v). ZnCl-terminated samples were stored in the dark and at room temperature.

From the gas headspace of the exetainers, the production of ^14^N^15^N and ^15^N^15^N was determined by gas chromatography isotope ratio mass spectrometry (GC–IRMS; Isoprime equipped with an autosampler) to determine rates of denitrification. Whereas, to determine potential ^15^NH_4_^+^ production related to DNRA, we converted the dissolved NH_4_^+^ fraction in the incubations to N_2_ gas using the alkaline-hypobromide method^56^. After the chemical conversion, we measured the ammonium-derived ^14^N^15^N using GC–IRMS analysis. The rates of dinitrogen (N_2_) gas and NH_4_^+^ production as a function of time were calculated using the linear regression slope, with the standard error derived from the deviation in the slope across the time points. We highlight that the time courses used for rate calculations were strictly linear (Supplementary Fig. 4). On the basis of the measured rates of denitrification and DNRA activity, the limit of detection was 0.001 nmol cm^−3^ h^−1^ and 0.002 nmol cm^−3^ h^−1^, respectively.

Due to the low anammox activity in Baldeggersee (based on Exp2; Fig. 1b), we consider that essentially all ^29^N_2_ production from ^15^NO_3_^−^ experiments (Exp1) was derived from denitrification. Under these circumstances, we can calculate the potential rates of total denitrification (*D*) according to a previous study^57^, using the following equations:

3 $${D}_{15}={P}_{29}+2\times {P}_{30}$$

4 $${D}_{14}=\frac{{P}_{29}}{2\times {P}_{30}}\times \left({P}_{29}+2\times {P}_{30}\right)$$

5 $$D={D}_{15}+{D}_{14}$$

where D^15^ and D^14^ represent denitrification rates derived from labelled (^15^N) and unlabelled (^14^N) nitrate, respectively. In the above, *P*_29_ and *P*_30_ refer to the production rates of ^29^N_2_ and ^30^N_2_ gas, respectively. Following the quantification of denitrification, we then determined potential DNRA activity in ^15^NO_3_^−^ experiments (Exp1) according to the following equations:

6 $$\mathrm{DNRA}={P}_{15}/{F}_{{\rm{N}}}$$

7 $${F}_{N}={15N}_{{\mathrm{substrate}}}/{15N}_{{\mathrm{substrate}}}+\,{14N}_{{\mathrm{substrate}}}$$

In equation (6), *P*_15_ refers to the production of ^15^NH_4_^+^. In equation (7), the ^15^N_substrate_ and ^14^N_substrate_ refer to the concentration of the labelled ^15^NO_3_^−^ and the ambient nitrate concentration in the incubation experiment, respectively. Although the ^15^NO_3_^−^ slurry incubations contained some oxygen—added intentionally to mimic bioirrigated sediment conditions (see above)—this could, in theory, stimulate nitrification and introduce unlabelled ^14^NO_3_^−^, potentially diluting the ^15^NO_3_^−^ tracer and complicating rate calculations. However, we consider this effect to be negligible. On the basis of our enumerated Thaumarchaeota cell abundances and known single-cell activity rates for nitrifiers^24^, we estimate nitrification rates in Baldeggersee to be extremely low, at 0–0.004 nmol cm^−3^ h^−1^ (Fig. 1b), two orders of magnitude lower than our measured denitrification rates. Moreover, nitrifiers show slow doubling times (>24 h per division^58^), making substantial biomass growth unlikely during the ~36-h incubation period, even with oxygen present.

In parallel with the ^15^N-dissolved inorganic nitrogen (DIN) experiments (Exps. 1 and 2), we applied a protocol previously used for water column incubations^22^, to determine potential rates of labile organic matter remineralization to ammonium at different sediment depths. The same protocol was applied as in the ^15^N-DIN incubations, except that these experiments were amended with ^15^N-lyopholized algal biomass (98% ^13^C- and ^15^N-labelled *Spirulina platensis*, Sigma-Aldrich). To determine ^15^NH_4_^+^ production (from ^15^NO_3_^−^ and ^15^N-algal additions), ammonium was converted to N_2_ gas using the alkaline-hypobromide method. Following its chemical conversion, ^14^N^15^N was quantified by GC–IRMS analysis. The linear regression slope of ^29^N_2_ (or ^15^NH_4_^+^) production over time was used to calculate potential remineralization rates. Unlike in equation (6), background labelling was not corrected because *F*_n_ ≈ 1 due to near-complete isotopic labelling. These experiments examined potential labile organic matter turnover to ammonium between the mixed and stratified regimes.

### Quantifying water column ammonium turnover by nitrification–assimilation

Nitrification rates were determined using ^15^NH_4_^+^ tracer addition experiments following established protocols^59^, previously applied in Baldeggersee^39^. In brief, water samples were incubated in 250-ml serum bottles amended with ^15^NH_4_^+^ to a final concentration of 5 µM using a 200-mM tracer stock solution. Unlike the original protocol, incubations were not helium-purged to maintain oxic conditions consistent with in situ conditions. After tracer addition, samples were mixed and aliquoted into 12-ml exetainers (Labco). Incubations were terminated at 0 h, 6 h, 12 h, 24 h and 36 h by creating a 2-ml helium headspace followed by the addition of 100 µl ZnCl_2_ (50% w/v). Samples were stored in the dark at room temperature. Ammonium was converted to N_2_ using the alkaline-hypobromite method, and the resulting ^14^N^15^N was measured by GC–IRMS (Isoprime). Uptake rates were calculated from the slope of the linear regression of ^15^NH_4_^+^ consumption over time. Background water column NH_4_^+^ concentrations were low (~1 µM), minimizing isotope dilution effects.

### Microbial metagenome genome assembly

We recovered Baldeggersee MAGs from raw reads generated previously^31^ (Supplementary Tables 1 and 3). We focused our analysis on March and September 2018, representing the end of the mixed and stratified periods, respectively (Fig. 3b). Raw sequence reads were uploaded to the metagenomic analysis platform KBase^60^. Raw Illumina HiSeq 2500 forward and reverse reads (2 × 150 bp) were merged into paired-end libraries, which were then filtered to remove sequence adaptors and low-quality reads using Trimmomatic (v0.36)^61^. A total of 39,869,043 and 35,494,554 reads were generated for the mixed and stratified samples, respectively (Supplementary Table 3). De novo assembly of the quality-controlled paired-end reads was performed using metaSPAdes (v3.15.3)^62^. Assembled contig libraries were then subjected to metagenome binning to produce MAGs using MetaBAT2 (v1.7)^63^, MaxBin2 (v2.2.4)^64^ and CONCOCT (v1.1)^65^. MAG completeness was verified by checkM^66^, and the recovered MAGs were annotated using RAST (v1.073)^67^. Phylogenetic trees were constructed with the maximum-likelihood method using SpeciesTreeBuilder (v0.1.4), which analyses 49 universal genes defined by clusters of orthologous groups^68^. We used the nitrogen cycling database of FAMA (v1.1) to search for key genes involved in denitrification, DNRA and nitrification^69^. In addition, we paired this analysis with DRAM (v0.1.2) to provide a larger overview of MAG carbon metabolisms and CAZyme-related genes^70^. To determine the relative abundance of a MAG within the microbial community, it was mapped to the sequenced reads of a queried sample using Bowtie2 (v2.3.2)^71^. The number of mapped reads was normalized to the total reads to obtain reads per kilobase per million mapped reads (RPKM).

### Cell enumeration

We used CARD-FISH to determine the cell abundances of Bacteroidales and Methylophilales (covering the genus *Methylotenera*; Supplementary Table 4), following protocols established elsewhere^72,73^. First, a sediment core was collected and sectioned according to the depths outlined in Fig. 1b,c. Collected sediment was then fixed with formaldehyde (final concentration, 1%) for 1 h at room temperature. The sediment was then centrifuged (16,000 × *g*) for 5 min and resuspended in 1× PBS buffer (pH 7.6). This step was repeated two more times. The sediment was then sonicated (7 × 30 s) to detach microbial aggregates. After the vortexing and centrifuging (16,000 × *g*; 5 min) of the samples, the supernatant was filtered onto polycarbonate filters (200 mbar vacuum pressure). The samples were washed twice with sterile Milli-Q water and air-dried.

The CARD-FISH procedure was conducted on samples collected on the polycarbonate filters. Briefly, filter fragments were treated with lysozyme (10 g l^−1^) for 45 min at 37 °C to permeabilize the cells for hybridization. Subsequently, the filters were washed in PBS and then in Milli-Q water before inactivating endogenous peroxidases using a methanol–hydrogen peroxide (0.15%) treatment for 10 min at room temperature. Filters were washed with Milli-Q water before hybridization. Filter fragments were incubated for 3 h at 46 °C in the hybridization buffer containing the target probe and its corresponding formamide concentration (Supplementary Table 4). Filters were washed in pre-warmed washing buffer containing 0.1 M NaCl, 5 mM EDTA (pH 8.0), 20 mM Tris–HCl (pH 7.5) and 0.01% SDS for 15 min at 48 °C. Filters were subsequently washed for 10 min in 1× PBS at room temperature. Finally, filter fragments were incubated for 45 min at 46 °C in an amplification buffer containing 0.15% H_2_O_2_ and 20 µg Oregon Green-labelled Tyramide. After washing in 1× PBS and Milli-Q, the filters were dried before staining with 4′,6-diamidino-2-phenylindole (DAPI; 1 µg ml^−1^) for 10 min at room temperature. The filter fragments were then embedded in a mixture of Citifluor/Vectorshield and DAPI, and the probe-hybridized signals were counted using an epifluorescence microscope (Zeiss AxioPlan). Up to 1,000 DAPI-stained cells from 10 different fields of view were counted. CARD-FISH probe EUB338 served as a positive control, while NON338 served as a negative control.

### Chitin and pectin quantification

To determine the concentrations of chitin monomers (glucosamine and *N*-acetyl-glucosamine) and pectin monomers (galacturonic acid) in sediment, we followed the hydrolysis–purification protocol of ref. ^74^. Sediment samples were heated with 6 M HCl (10 ml) in a closed hydrolysis centrifugation flask (filled with dinitrogen gas) for 12 h at 105 °C. After cooling, the slurry was centrifuged (2,000 × *g*; 5 min) and the supernatant was decanted and completely dried using a rotary evaporator at 40 °C under vacuum. Subsequently, the residue was dissolved in 20 ml of water, and the pH was adjusted to 6.6–6.8 using a 0.4-M KOH solution. The precipitate was removed by centrifugation (2,000 × *g*; 10 min), and the clear supernatant was freeze-dried or evaporated (40 °C). Finally, the residue was dissolved in 3 ml of dry methanol and centrifuged (2,000 × *g*; 10 min) to eliminate any remaining salts. The amino sugar fraction was diluted in Milli-Q water and then analysed by liquid chromatography–mass spectrometry (ThermoScientific). The instrument was operated at a flow rate of 1 ml min^−1^ with acetic acid eluent (0.77 g l^−1^, 0.1% acetic acid) in atmospheric pressure chemical ionization (APCI) mode (vaporizer temperature, 550 °C; capillary temperature, 250 °C). We quantified the masses pertaining to galacturonic acid (*m*/*z* = 193) and glucosamine–*N*-acetyl-glucosamine (*m*/*z* = 178/220), which are major constituents of pectin and chitin, respectively. Identified peaks were quantified by normalization to their respective standards (Sigma-Aldrich).

### Model description and set-up

To mimic hydrodynamic and biogeochemical conditions observed in situ, we used the one-dimensional General Lake Model (GLM; v3.1) coupled with the Aquatic EcoDynamics (AED; version 2) module. The open-source model has gained recognition for its ability to simulate hydrodynamics, phytoplankton dynamics and biogeochemical conditions in pristine and engineered lakes^52,53,75,76^.

The meteorological forcing data required to run the GLM include shortwave radiation, longwave radiation, cloud cover, air temperature, relative humidity, rainfall and wind speed. Raw forcing data were obtained for Baldeggersee from MeteoSwiss (Swiss Federal Office of Meteorology and Climatology) and were processed to calculate wind speed, relative humidity and longwave radiation. Longwave radiation was estimated by the GLM model using the cloud cover fraction data. Wind speed was calculated using:

8 $$\mathrm{ws}=\sqrt{{u}^{2}-{v}^{2}}$$

where *u* is the zonal wind (eastward wind) and *v* is the meridional wind (northward wind). Relative humidity was derived using equation (9), with the saturation vapour pressure calculated from the August–Roche–Magnus approximation (equation (10)).

9 $$\mathrm{Relative}\,\mathrm{humidity}=\frac{\mathrm{actual}\,\mathrm{vapour}\,\mathrm{pressure}}{\mathrm{saturation}\,\mathrm{vapour}\,\mathrm{pressure}}\times 100 \%$$

10 $$\mathrm{Saturation}\,\mathrm{vapour}\,\mathrm{pressure}\,=\,6.11\times {10}^{\left(\displaystyle\frac{7.5\times T}{T+237.3}\right)}$$

The calibration of the model was achieved through a two-step process. Firstly, the GLM component was calibrated to reproduce water column temperature data by adjusting the physical parameters detailed in Supplementary Table 5. The model was validated with Baldeggersee observational data collected from 2017 to 2022, with water temperature validation yielding a root mean square error (RMSE) of 1.7 °C (Supplementary Figs. 5 and 6a,b). From this, we could define the stratification period, which was delineated when a ≤1 °C difference developed between top and bottom waters^77^. In the model, the onset of stratification began on 29 April and terminated on 29 December, with the mixed regime lasting 121 calendar days. On the basis of our observations, changes to the thermal structure of the lake began in mid-April, with a 1.6 °C difference reported between top and bottom waters on 12 April. Moreover, the onset of the deterioration of the stratified regime began in mid-November, as the temperature difference between top and bottom waters subsided to 1.5 °C, coinciding with a deepening of the thermocline from 25 m to 40 m. Thus, based on the observation data, complete mixing will probably proceed in late November to early December. For comparison with the model, we also used 29 April to 29 December to delineate the stratified regime for observational data.

### Modelling of POC

We implemented phytoplankton groups in AED to represent blooms of cyanobacteria and diatoms (Supplementary Table 7). The flux of POC to the sediment was assessed in the model and compared with sediment trap observations taken from 2017 to 2018^19^ (Fig. 2d and Extended Data Fig. 4a,b). The model was able to simulate the vertical POC flux associated with primary and secondary phytoplankton blooms, as well as the overall length of the summer bloom period.

Although vertical export of euphotic-zone POC is well represented in the 1D model, it does not capture all components of the total POC flux. In particular, lateral sediment POC refocusing—in which particulate material from the lake margins is transported towards the deepest basin, a common feature of lakes with sloping bathymetry^78^—is not parameterized in the base model. Observations from sediment traps in Baldeggersee provide evidence for this process: traps positioned near the lake bottom (3 m above the sediment at the deepest point) consistently recorded higher POC fluxes than traps located in the upper water column (15 m depth; Extended Data Fig. 4a). This contrast is pronounced in late summer and continues into the mixed period, when bottom-trap fluxes exceed those measured in the upper trap over the same interval. Material collected during the mixed period was also strongly depleted in nitrogen relative to carbon, indicating advanced degradation and supporting a lateral origin from terrestrial and/or reworked aquatic organic matter (Extended Data Fig. 4b). To quantify the refocused POC flux, we calculated the difference between POC fluxes measured in the lower and upper traps (Extended Data Fig. 4a,b). Seasonally, POC refocusing is minimal in spring, increases through late summer and autumn, and remains elevated throughout the winter mixed period (Extended Data Fig. 4b,c). Furthermore, refocused POC fluxes were strongly correlated with wind-driven orbital velocities, a proxy for wave-induced near-bed shear stress, which is derived from the meteorological lake data (Extended Data Fig. 4b,d; Pearson two-tailed *P* < 0.01, d.f. = 12). This relationship indicates that POC refocusing is strongly coupled to wind forcing that, in turn, governs surface wave activity and thereby the near-bed orbital velocities that promote sediment resuspension and lateral transport.

To represent this process in the model, we linked the refocused POC flux to the modelled orbital velocities, which are already resolved in GLM. A calibration curve was applied that defined a linear relationship between orbital velocity and the refocused POC flux, such that flux delivery scaled proportionally with modelled orbital intensity (Extended Data Fig. 4e). As an outcome, the mean refocused POC flux associated with the model was 7.8 mmol m^−2^ d^−1^, matching the average value inferred from observations. Incorporating this mechanism led to a substantially greater delivery of total POC (refocused plus euphotic-zone-derived fluxes) to the sediments than in the original base model. During the mixed regime, the modelled POC flux increased from 0.5 mmol m^−2^ d^−1^ to 12.9 mmol m^−2^ d^−1^, bringing it much closer to the observed value of 17.7 mmol m^−2^ d^−1^ (Extended Data Fig. 5a). Although the effect is strongest in the mixed period, owing to higher orbital velocities, improvements are also evident during stratification, in which modelled fluxes increased from 10.7 mmol m^−2^ d^−1^ to 15.0 mmol m^−2^ d^−1^, approaching the observed summer flux of 20.8 mmol m^−2^ d^−1^. The refocused model further produced a more realistic contrast between mixed and stratified conditions and improved simulation of downstream processes, including $R_{\mathrm{POC}:\mathrm{NO}_3^-}$ (Extended Data Fig. 5b), denitrification (Extended Data Fig. 5c) and especially DNRA activity (Extended Data Fig. 5d).

### Modelling of oxygen and nitrate

Organic matter mineralization drives seasonal oxygen and nitrate dynamics. Sediment-trap observations from Baldeggersee indicate that euphotic-zone-derived POC flux, the more reactive fraction, was significantly correlated with the nitrate flux (Extended Data Fig. 4f; Pearson two-tailed *P* < 0.05; d.f. = 8). By contrast, refocused POC, representing more degraded material, showed no significant relationship with dissolved nitrate or oxygen fluxes (*P* > 0.05). Accordingly, in the model, vertical euphotic-zone POC export was treated as the primary driver of seasonal nitrate and oxygen turnover. Next, oxygen and nitrate dynamics were further calibrated by adjusting standard parameters in the water quality AED package as outlined in Supplementary Table 6. In addition, we made a modification to nitrification, which led to more accurate simulations of dissolved oxygen and nitrate concentrations in the waters overlying the sediment. Nitrification was calculated using the following equation:

11 $${A}_{\mathrm{nitrif}}^{\mathrm{mod}}={R}_{\mathrm{nitrif}}{\theta }_{\mathrm{nitrif}}^{T-20}{\varPhi }_{\mathrm{DON}}^{\mathrm{nitrif}}\left[\mathrm{DON}\right]{\mathrm{NH}}_{4}$$

where *θ*_nitrif_ is an Arrhenius temperature parameter specific to nitrification, *T* is the temperature generated by the model and DON represents dissolved organic nitrogen. In the default model setting, oxygen was coupled to nitrification activity. However, we found that this resulted in unrealistic background concentrations of oxygen, nitrate and ammonium that deviated from in situ observations. Alternatively, coupling nitrification to DON yielded a better match to observed nitrification rates and water column nutrient concentrations. Nitrifiers have been shown to depend or rely on dissolved inorganic N pools, such as urea and cyanate^79^.

To quantify the fluxes of oxygen and nitrate (in mmol m^−2^ d^−1^) from the water column into the sediment, we used Fick’s first law:

12 $$J=-D\frac{{dC}}{{dz}}$$

Here *D* represents the diffusion coefficient of the nutrient in water or sediment (m^2^ d^−1^), while *dC*/*dz* denotes the concentration gradient (change in concentration per unit depth). The initial concentration in the overlying water is a key factor in determining the flux, as the diffusion coefficient and penetration depth show only minor seasonal variations. To ensure model accuracy, we first validated its ability to simulate oxygen and nitrate concentrations in the overlying waters at 65 m depth. The model was calibrated using data from 2017 to 2022, with validation against observed oxygen (RMSE = 87 µmol l^−1^; Supplementary Fig. 6c) and nitrate (RMSE = 29 µmol l^−1^; Supplementary Fig. 6d) concentrations in the lake bottom waters. The validated oxygen and nitrate concentrations were then used as inputs to calculate fluxes (equation (12)), along with the previously measured diffusion and penetration depth values^19^. Finally, the modelled fluxes were compared with seasonal field observations from multiple sampling stations in Baldeggersee, generating a robust dataset (Fig. 2f,h).

### Modelled rates of denitrification and DNRA

While the GLM–AED model is effective at simulating lake physics and the nitrate flux from the water column into the sediment, it is not yet capable of determining the fate of the nitrate flux and whether it is reduced to dinitrogen gas (via denitrification) or to ammonium (via DNRA). However, given the close correlation of *R*_DNRA:Den_ to the relative availabilities of organic carbon and nitrate ($R_{\mathrm{POC}:\mathrm{NO}_3^-}$), we leveraged this relationship to accurately simulate denitrification and DNRA in the model. On the basis of the positive linear relationship between *R*_DNRA:Den_ to $R_{\mathrm{POC}:\mathrm{NO}_3^-}$ (*t*-test, *R*^2^ = 0.94, *P* < 0.01; Extended Data Fig. 3c), we derived a proportionality coefficient of 0.0504 (*ω*^obs^). We then applied this coefficient alongside the model’s $R_{\mathrm{POC}:\mathrm{NO}_3^-}$ output values to determine the ratio between DNRA and denitrification:

13 $${R}_{\mathrm{DNRA}:\mathrm{Den}}^{\mathrm{mod}}={\omega }^{\mathrm{obs}}\times {R}_{\mathrm{POC}:\mathrm{NO}_3^-}^{\mathrm{mod}}$$

Next, we converted the *R*_DNRA:Den_ ratio into a percentage for DNRA (versus total nitrate reduction; %_DNRA_×) and multiplied it by the nitrate flux value (${F}_{\mathrm{NO}_3^-}$) to estimate the DNRA rate. Finally, we subtracted the DNRA rate from the nitrate flux to calculate the actual denitrification rate:

14 $$A_{\mathrm{DNRA}}^{\mathrm{mod}} = \%_{\mathrm{DNRA}} \times F_{\mathrm{NO}_3^-}$$

15 $${A}_{\mathrm{denit}}^{\mathrm{mod}}={F}_{\mathrm{NO}_3^-}-{A}_{\mathrm{DNRA}}^{\mathrm{mod}}$$

### Future scenario modelling

After simulating denitrification and DNRA in Baldeggersee, we then examined how future climate change projections influence these nitrogen transformation processes. To force the GLM–AED model, we used future climate change projections generated from the Norwegian Earth System Model (NORESM), which is one of several global climate models^12^. NORESM was selected because it includes modifications tailored to Nordic climates and shows a more conservative response to greenhouse gas forcings compared with other models in the EURO-CORDEX regional climate model ensemble^77^. The NORESM data used in this study, provided previously^14^, underwent global climate model downscaling to a European domain resolution of 44 km and were subsequently calibrated to local Swiss weather stations (MeteoSwiss) for improved regional accuracy. As downscaled meteorological forcing data were not specifically available for Baldeggersee, the GLM–AED model was instead forced with data from nearby Lake Zurich. Lake Zurich is situated 556 m above sea level, an altitude comparable to that of Baldeggersee, which is 463 m above sea level. Moreover, Lake Zurich has a similar topographical setting to Baldeggersee, as both are surrounded by moderately sloping hillsides of the Pre-Alps.

The GLM–AED model was run from 1980 to 2099 using NORESM future projection forcing data under RCP 2.6 and RCP 8.5, representing low and high greenhouse gas emission scenarios, respectively. During the future projection period, water column temperature, POC and oxygen values remained stable and deviated little from the baseline. However, water column nitrate concentrations showed a ‘runaway’ deviation from the baseline but could be corrected using a polynomial function. After corrections were applied, we converted the oxygen and nitrate bottom water concentration values into fluxes according to equation (12).

Calculating the relative impact of the RCP scenarios on the lake physics and biogeochemistry was done according to the conventions of the Swiss Climate Change Scenarios CH2018 climate analyses. Namely, a 30-year baseline period from 1982 to 2010 was used, with the first year of the simulation discarded to exclude possible spin-up artifacts. For the future scenario calculations, a similar 30-year time interval was taken between 2071 and 2099^12^.

### Reporting summary

Further information on research design is available in the Nature Portfolio Reporting Summary linked to this article.

## Supplementary information

Supplementary Information Supplementary Figs. 1–6, Tables 1–7, Discussion and References.

Reporting Summary

Peer Review file

## Source data

Source Data Fig. 1 Plotting data and statistical source data.

Source Data Fig. 2 Plotting data and statistical source data.

Source Data Fig. 3 Plotting data and statistical source data.

Source Data Fig. 4 Plotting data and statistical source data.

Source Data Extended Data Fig. 1 Plotting data and statistical source data.

Source Data Extended Data Fig. 2 Plotting data and statistical source data.

Source Data Extended Data Fig. 3 Plotting data and statistical source data.

Source Data Extended Data Fig. 4 Plotting data and statistical source data.

Source Data Extended Data Fig. 5 Plotting data and statistical source data.

Source Data Extended Data Fig. 6 Plotting data and statistical source data.

Source Data Extended Data Fig. 7 Plotting data and statistical source data.

Source Data Extended Data Fig. 8 Plotting data and statistical source data.

## Data Availability

Rate measurement data are available via Zenodo at https://doi.org/10.5281/zenodo.15351261 (ref. ^80^). The recovered MAGs discussed in this study have been deposited in the NCBI database under accession code PRJNA1259374, while the original metagenomic data are available under PRJNA726540 (ref. ^31^). Parameters used to calibrate the GLM used in this study are provided in Supplementary Tables 5–7. Source data are provided with this paper.
