# Supplementary material for: Seasonality of lake microbial denitrification and its sensitivity to climate warming
Source: Nat Microbiol. 2026 May 22;11(7):1907–19. doi: 10.1038/s41564-026-02349-9 (PMC13661275; doi:10.1038/s41564-026-02349-9)
Supplement: Supplementary file 1 [file 41564_2026_2349_MOESM1_ESM.pdf]

nature microbiology

Supplementary information

https://doi.org/10.1038/s41564-026-02349-9

Seasonality of lake microbial denitrification and its sensitivity to climate warming

In the format provided by the authors and unedited

SUPPLEMENTARY DISCUSSION

Sediment oxygen dynamics and bio-irrigation

Due to the organic-rich nature of the sediments, oxygen penetration in Lake Baldegg is relatively shallow, reaching only a few millimeters in depth (e.g., Extended Data Fig. 1a), which is typical for lakes in Switzerland[1]. In addition, the sediments are heavily influenced by bio-irrigation due to the presence of macrofauna[2,3]. In Lake Baldegg, the sediments are dominated by Tubifex worms, with an estimated faunal density of approximately 8,500 individuals per m^2. These worms form dense burrow networks extending from 0 to 10 cm depth (Extended Data Fig. 1d). Tubifex worms, roughly 4-5 cm long, exhibit a head-down deposit feeding behavior. Thereby, their tails remain located a few millimeters in the oxic water column, while their heads are anchored 3-5 cm deep in the anoxic sediments. Through rhythmic tail undulations, the worms can take up and actively transport oxygen (via hemoglobin) from the bottom waters into the anoxic sediment layers. This oxygen transport is further supported by our sediment amendment experiments, in which the addition of excess Tubifex resulted in measurable oxygen accumulation between 1-6 cm depth (Extended Data Fig. 1a). Within this same zone (2-5 cm), we also observed nitrate and sulfate anomalies (Extended Data Fig. 1b, c), suggesting active solute exchange. Additionally, sulfide concentrations exhibited a minimum in the 1-6 cm depth range (Extended Data Fig. 1c), consistent with oxidative removal via oxygen or nitrate and further supporting the presence of a bioirrigated zone. These depth profiles collectively suggest that the otherwise anoxic sediments are intermittently exposed to oxygen and nitrate, creating conditions that could support both aerobic and anaerobic nitrogen transformation processes, which have also been observed in other sediment systems[4,5].

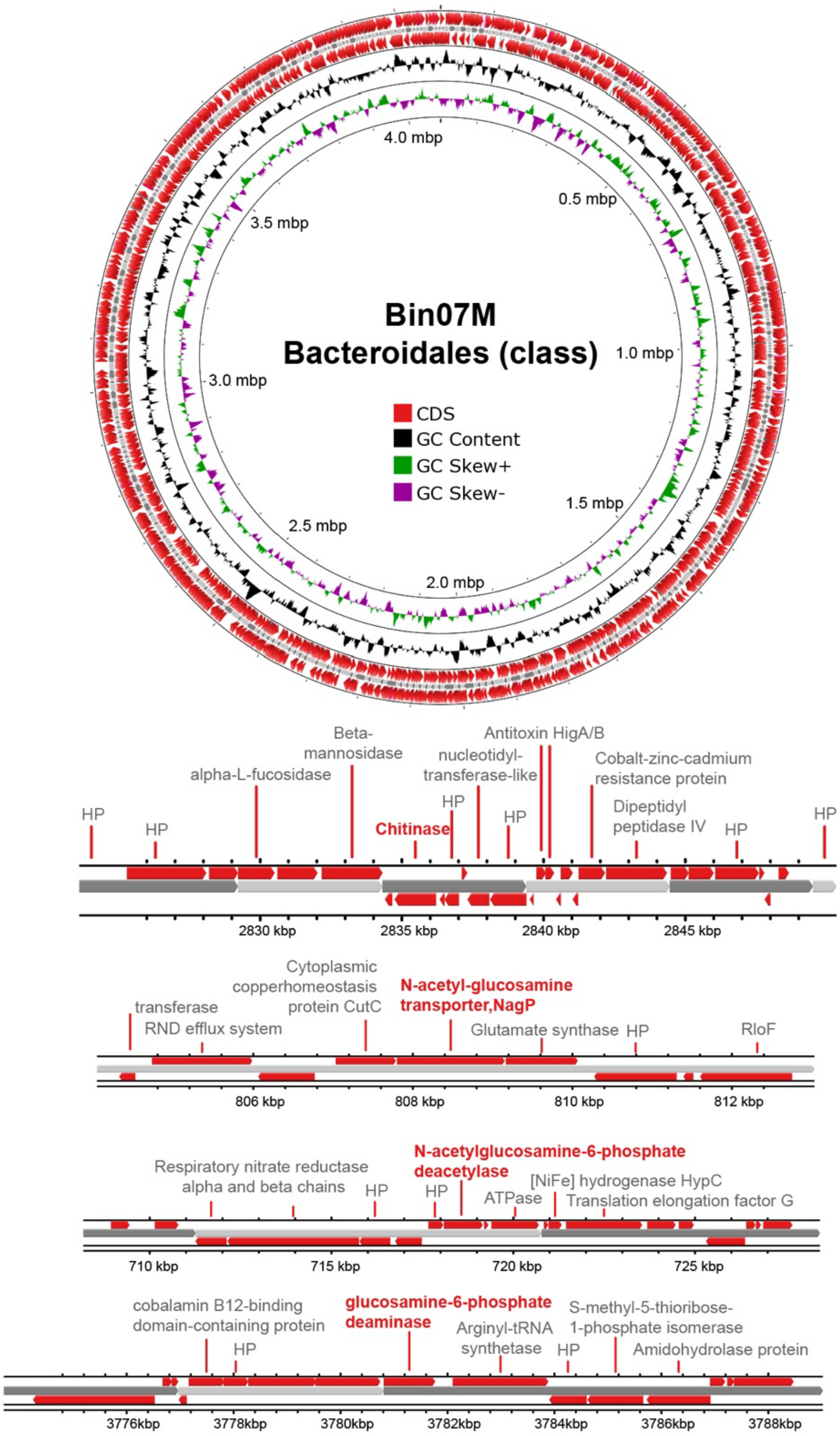

Supplementary Figure 1: Draft genome of the chitinolytic bacteria, Bin07M. (**left**) The whole draft genome of Bin07M was generated using Proksee[6]. Additional genome information can be found in Supplementary Table 3. **(right)** Key genes (annotated with RAST), including gene synteny, are shown. Abbreviations: HP, Hypothetical protein.

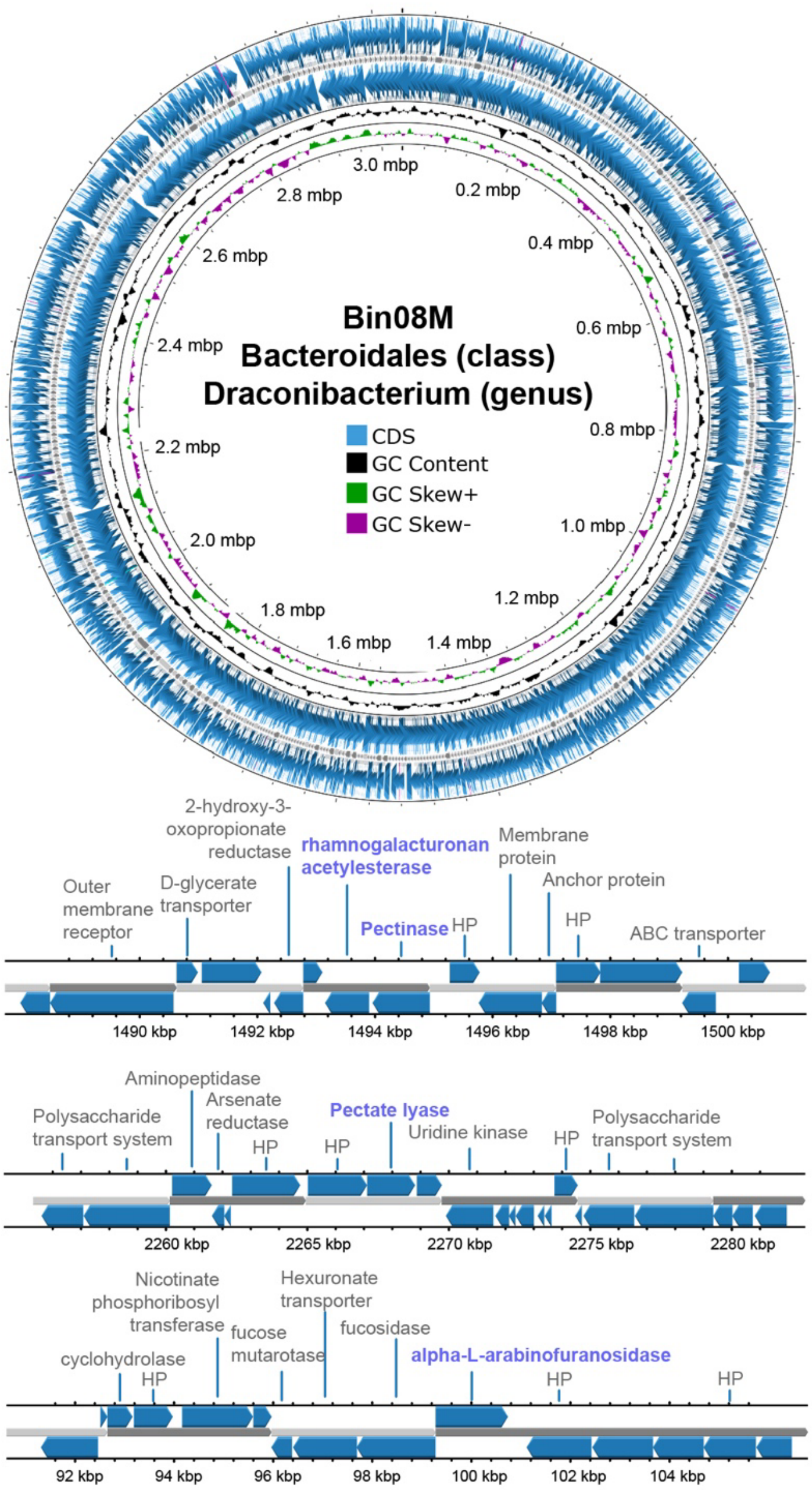

Supplementary Figure 2: Draft genome of the pectinolytic bacteria, Bin08M. (**left**) The whole draft genome of Bin08M was generated using Proksee[6]. Additional genome information can be found in Supplementary Table 3. **(right)** Key genes (annotated with RAST), including gene synteny, are shown. Abbreviations are as follows: HP, Hypothetical protein.

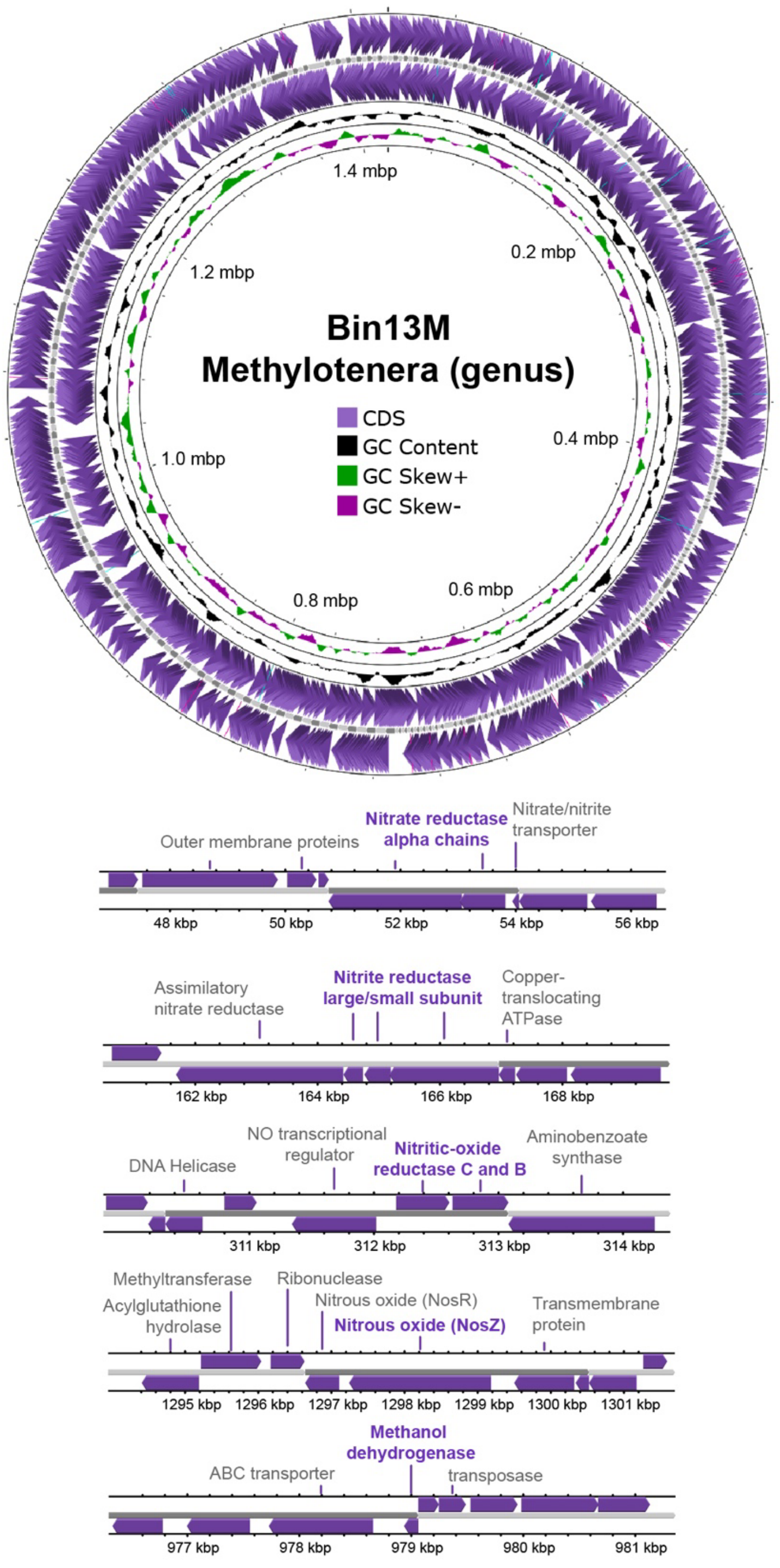

Supplementary Figure 3: Draft genome of the methanol-oxidizing nitrate-reducing bacteria, Bin13M. (**left**) The whole draft genome of Bin13M was generated using Proksee[6]. Additional genome information can be found in Supplementary Table 3. **(right)** Key genes (annotated with RAST), including gene synteny, are shown. Abbreviations are as follows: HP, Hypothetical protein.

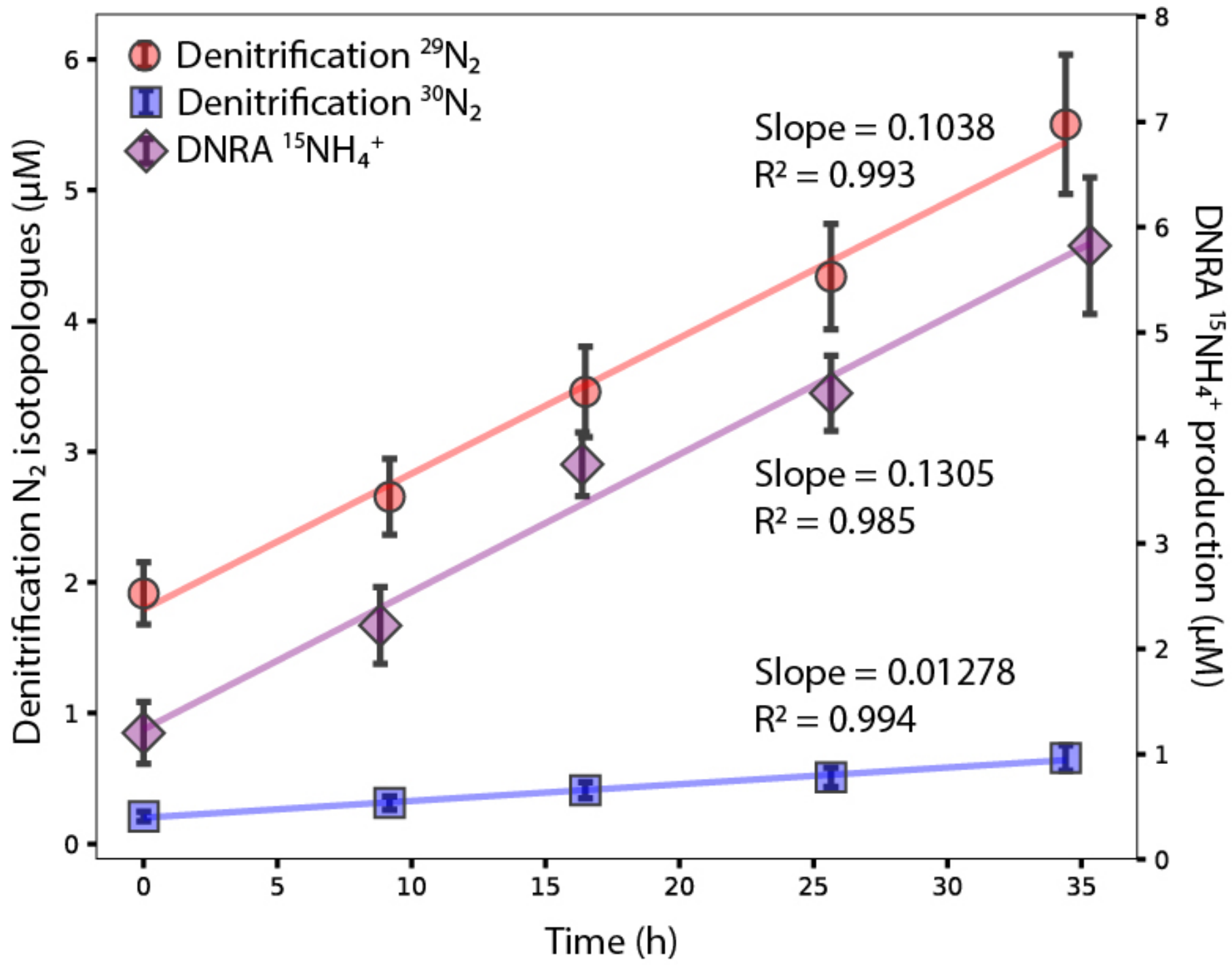

Supplementary Figure 4: Linearity of potential denitrification and DNRA rates over time. Seasonally aggregated samples are shown for denitrification (n = 58) and DNRA (n = 32). Linear regression lines are plotted for each respective isotopologue, with corresponding slope and R^2 values indicated.

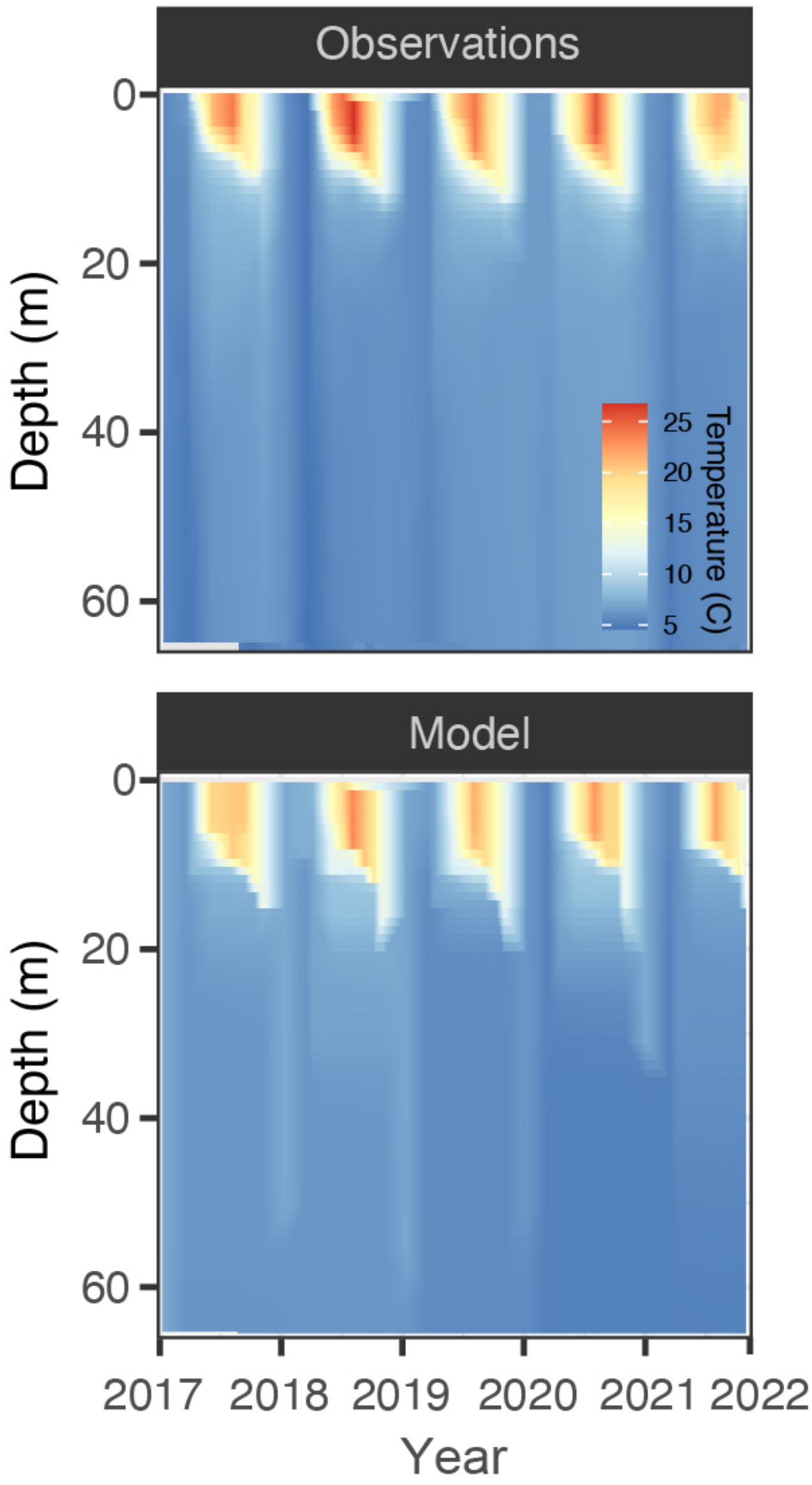

Supplementary Figure 5: Simulation of water column temperature in Baldeggersee using the GLM model. A side-by-side comparison of observational data and modeled temperatures is shown. The regional Canton of Luzern provided water column temperature profiles from 2017 to 2022, which were used to tune the GLM model. More details regarding model validation at discrete depths are shown in Supplementary Fig. 6.

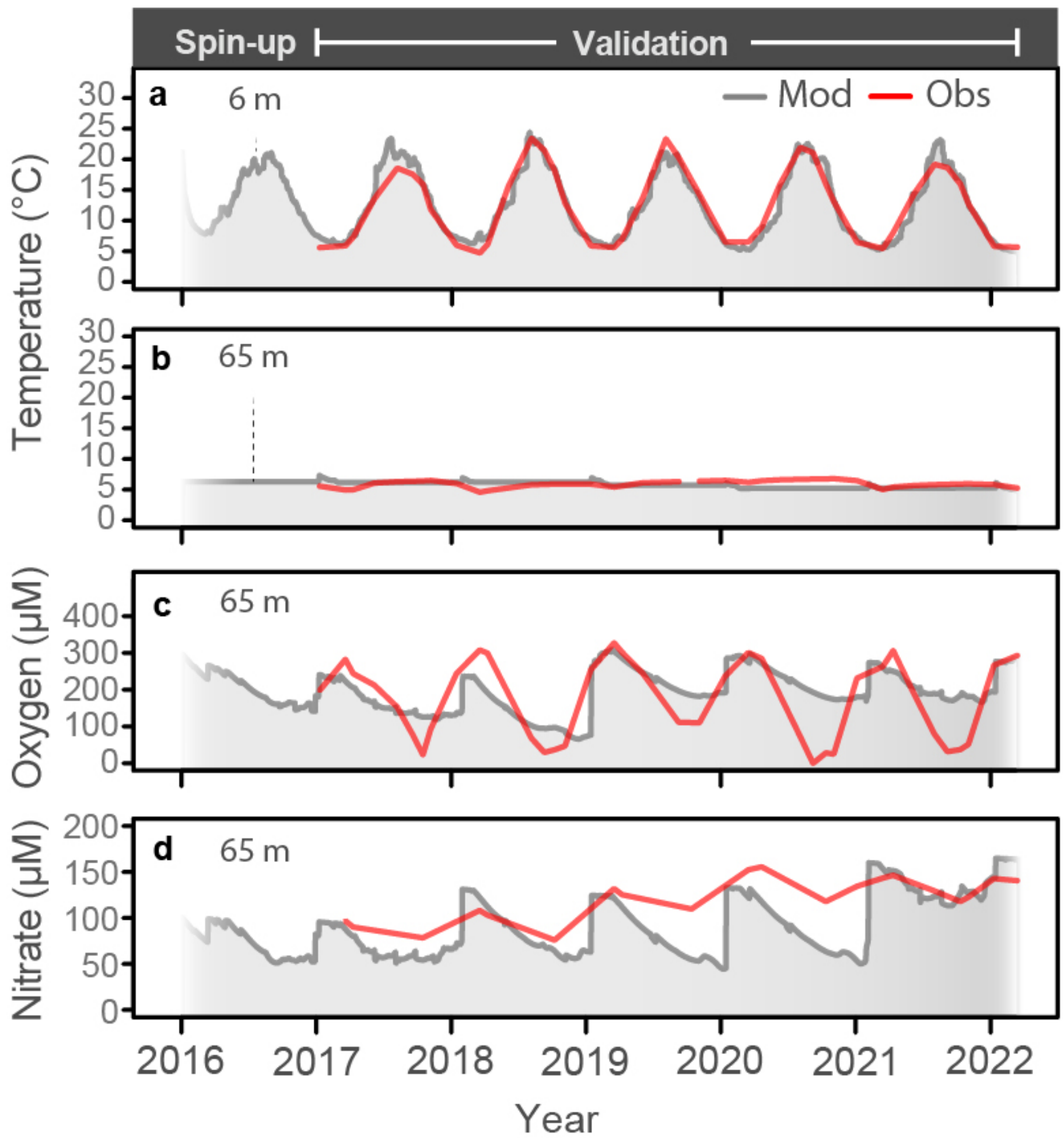

Supplementary Figure 6: Simulation of physical and biogeochemical parameters in the waters overlying Baldeggersee sediment. (**a, b**) Water temperatures at 6 and 65 meters depth, which capture the upper thermocline and the bottom waters of the lake, respectively. Water temperature at 6 m depth highlights the fluctuating dynamics associated with the mixing regimes, including the summer stratified and winter mixed periods. (**c**) Dissolved oxygen concentrations at 65 meters depth. (**d**) Water column nitrate concentrations at 65 meters depth. The regional Canton of Luzern provided water column temperature, oxygen, and nitrate profiles from 2017 to 2022. The first year of the model spin-up is excluded from the model validation.

Supplementary Table 1. Overview of sampling stations and collection dates for sediment and water column samples in Baldeggersee.

	[a] O_2 flux (bi-monthly)	[a] NO_3^- flux (bi-monthly)	[b] POC flux (bi-weekly)	[c] Chitin (bi-monthly)	[d] Metagenome (bi-monthly)	[c] ^{15}N-exps (bi-monthly)	[c,e] Water column exps. (bi-monthly)
Station A: 66 m (deepest) - 47° 11.929 (°N); 8° 15.613 (°E)							
2017-2018	X	X	X		X		
2021-2022				X		X	X
Station B: 36 m (mid)							
2017-2018	X	X	-	-		-	-
Station C: 15 m (outlet)							
2017-2018	X	X	-	-	X	-	-
Station D: 25 m (inlet)							
2017-2018	X	X	-	-		-	-
Station E: 15 m (inlet)							
2017-2018	X	X	-	-	X	-	-

[a] Oxygen and nitrate fluxes (directed from the water into the sediment) were collected by ref[7]

[b] The POC flux was determined from a sediment trap deployed at 15 m depth by ref[7]

[c] Were collected and analyzed in this study

[d] DNA samples were collected and sequenced by ref [8], whereas additional MAG analysis was performed in this study (Supplementary Tables 2; 3).

[e] Experiments performed in the water column include $^{15}NH_4^+$ additions, which were used to quantify rates of nitrification/assimilation (Fig. 1b, Extended Data Fig. 2).

Supplementary Table 2. Phylogenetic and quality characteristics of metagenome-assembled genomes (MAGs). Listed are MAGs recovered from the mixed (M) and stratified (S) regimes, with draft genomes exceeding 50% completion, and contamination scores < 5. Among these MAGs, 11 were detected in both mixed and stratified regimes, whereas 6 were exclusive to one of the two mixing regimes. If a MAG did not meet the quality threshold, it is marked as not applicable (NA). To determine the relative abundance of a MAG within the microbial community, metagenomic reads were mapped to each MAG of interest using Bowtie2 (v2.3.2; ref[9]), normalized to the total reads, and subsequently compared across regimes. MAG enrichments are presented as a percent increase relative to the opposing regime. MAGs in bold denote key marker species discussed in the main text and in Figure 3.

MAG Taxonomy	MAG_ID	Completeness (%)	Contamination	GC (%)	Genome size (bp)	N50	Relative increase
**Desulfobacterota, Syntrophia, Syntrophales, UBA4778, UBA2192**	Bin02M	71.25	3.06	60.70	1768559	4056	
	Bin02S	59.38	0.22	61.10	1236725	4338	
**Desulfobacterota, Desulfobacteria, Desulfobacterales, UBA2174_A, SpSt-501**	Bin04M	74.52	0.65	62.50	3069444	4472	
	Bin04S	69.44	0.65	62.70	2678878	5321	
**Pseudomonadota, Gammaproteobacteria, Burkholderiales, Rhodocyclaceae, UBA2250**	Bin05M	90.01	4.92	60.20	3395704	23310	
	Bin05S	61.80	4.18	60.10	2830973	4421	
**Desulfobacterota, Syntrophorhabdia, Syntrophorhabdales, Syntrophorhabdaceae, Syntrophorhabdus**	Bin06M	67.53	0.32	46.50	1675594	4199	+4%
	NA	<50	>5				
**Bacteroidota, Bacteroidales, Bacteroidales 4484-276, CAJATL01**	Bin07M	74.66	1.88	41.70	4086574	9103	+79%
	NA	<50	>5				
**Bacteroidota, Bacteroidia, Bacteroidales, Prolixibacteraceae, Draconibacterium**	Bin08M	58.57	3.72	40.20	3037799	3672	+74%
	NA	<50	>5				
**Pseudomonadota, Gammaproteobacteria, Xanthomonadales, Xanthomonadaceae, Arenimonas**	Bin09M	59.26	0.86	60.50	1294128	3498	
	Bin09S	58.75	1.53	60.60	1269242	4445	
**Actinomycetota, Acidimicrobiia, IMCC26256, PALSA-555, SHUY01**	Bin10M	73.65	1.28	53.30	1418950	6365	
	Bin10S	57.80	1.89	53.30	1372330	4187	
**Planctomycetota, Planctomycetia, Pirellulales, UBA1268, QWPN01**	Bin11M	93.01	1.15	70.30	3743955	49733	
	Bin11S	69.49	2.4	70.10	2855133	5429	
**Pseudomonadota, Gammaproteobacteria, Burkholderiales, Rhodocyclaceae, Azonexus**	Bin12M	59.29	0.34	66.50	1337608	6341	
	Bin12S	50.24	0.03	66.30	1062609	6664	
**Pseudomonadota, Gammaproteobacteria, Burkholderiales, Methylophilaceae, Methylotenera**	Bin13M	66.55	1.75	43.00	1421961	4260	+18%
	NA	<50	>5				
**Desulfobacterota, DSM-4660, Desulfatiglandales, HGW-15, JAFGDM01**	Bin14M	73.71	1.96	53.90	3442825	4169	
	Bin14S	74.49	0.77	54.40	2543058	3991	
**Bacteroidota, Bacteroidia, Bacteroidales, VadinHA17, SR-FBR-E99**	Bin15M	67.62	2.33	49.40	2005624	7530	+49%
	NA	<50	>5				
**Halobacteriota, Methanomicrobia, Methanomicrobiales, Methanoregulaceae, Methanoregula**	aBin17M	84.55	1.31	48.50	1222210	8021	
	Bin17S	77.68	4.4	48.60	1229648	6988	
**Bacteroidota, Bacteroidia, Bacteroidales, UBA4417, UBA4417**	Bin18M	73.19	1.68	41.00	3494429	9848	+131%
	NA	<50	>5				
**Desulfobacterota, Desulfomonilia, Desulfomonilales, Desulfomonilaceae, CAJBEZ01**	Bin19M	89.03	0	46.50	3386916	17907	
	Bin19S	91.94	0	46.50	3413851	14616	
**Desulfobacterota, Syntrophia, Syntrophales, Smithellaceae, UBA8904**	Bin21M	73.87	2.6	48.40	1742540	5707	
	Bin21S	79.49	3.25	48.50	1748355	6439	
	NA	<50	>5				
**Desulfobacterota, Syntrophia, Syntrophales, UBA2185, UBA2185**	Bin19S	87.53	4.95	48.00	2106114	7834	+19%

Supplementary Table 3. Metagenomic sequencing information from samples processed by ref [8]. In addition, this table provides further information regarding the metagenome-assembled genome (MAG) assembly process performed in this study.

**Metagenomic analysis performed by Baumann et al., 2022**								**MAG assembly (this study)**	
Sample name	Month	[1]Station	Sediment depth	Sequcences No	Filtered sequences No	No. Nitrogen genes	Sum-normalized N genes (gpm)	Contig No.	N50
SRR14488676	March	A	0-3 cm	39,869,043	39,143,311	3912	5658	29,184	4062
SRR14488670	September	A	0-3 cm	35,494,554	34,796,013	3321	5418	20,807	3672

[1] The deepest station (A) at Baldeggersee has a maximum water depth of 63 m and is situated at 47.19714795 °N; 8.26166454 °E

Supplementary Table 4: Probe sequences used for CARD-FISH, with formamide concentrations chosen according to the cited references.

Type	Sequence 5' - 3'	Formamide %	Specificity	[a] Coverage %	Reference
BAC303	CCAATGTGGGGGACCTT	0	Bacteroidales	65	[10]
MET1216	TTACGTGTGAAGCCCTGGC	35	Methylophilales (covering the genus *Methylotenera*)	87	[11]

[a] Coverage of the target clade is indicated, which is based on the Silva database (SILVA taxonomy release 138.2) using TestProbe (v3.0).

Supplementary Table 5: Calibrated parameters used in the General Lake Model (GLM). The default values are according to the GLM manual.

Parameter	Description	Default	Calibration value	Unit
**Water column**				
Max layers			500	
Coef_mix_conv	Mixing efficiency - convective overturn	0.2	1.4	-
Coef_wind_stir	Mixing efficiency - wind stirring	0.23	1.3	-
Coef_mix_hyp	Mixing efficiency of hypolimnetic turbulence	0.5	0.6	-
Sw_factor	Scaling factor that is used to multiply the shortwave data that is read in.	1	0.84	-
Lw_factor	Scaling factor that is used to multiply the longwave data that is read in.	1	0.84	-
Kw	Extinction coefficient for PAR radiation	0.2	0.15	m^{-1}
**Sediment**				
N_zones	Number of sediment zones	-	2	-
Sed_temp_mean	Sediment temperature	-	7, 7	°C
Sed_temp_amplitude	Change in sediment temperature over a year	-	0.5, 0.5	°C
zone_heights	Size of the sediment zones	-	0.9, 2	cm
**Stream inflows**				
Num_inflows	Number of streams to be simulated.	-	3	-
Names_of_strms	Names of the inflowing streams/rivers, separated by commas.	-	West, East, South	-
Flow speed	Daily flow rate	-	0.037[a]	$m^3 s^{-1}$
Oxygen	Daily average concentration	-	315[a]	mmol m^{-3}
Nitrate	Daily average concentration	-	220[b]	mmol m^{-3}
Ammonium	Daily average concentration	-	0.47[a]	mmol m^{-3}
DIC	Daily average concentration	-	68.7[a]	mmol m^{-3}

[a] Model values here were based on other model values set elsewhere.
[b] Stream inflow nitrate concentrations are based on measured values.

Supplementary Table 6: Calibrated nitrogen transformation parameters used in the Aquatic EcoDynamics (AED) model. The Default values are according to the AED manual.

	Description	Default	Calibration value	Unit
**Nitrification**				
Rnitrif	Kinetic rate constant	0.01	0.01	day^{-1}
Knitrif	Half saturation constant	62.0	62.0	$mmol\ m^{-3}$
Theta_nitrif	Temperature dependence	1.08	1.08	-
Nitrif_reactant_variable	Optional limitations	Oxygen	Dissolved inorganic nitrogen	-
**Denitrification**				
Rdenit	Kinetic rate constant	10.0	10.0	day^{-1}
Kdenit	O_2 inhibition	29.9	29.9	$mmol\ m^{-3}$
Theta_denit	Temperature dependence	1.1	1.1	-
Rdnra	Kinetic rate constant	0.01	0.01	day^{-1}
Kdnra_oxy	O_2 inhibition	0.4	0.4	$mmol\ m^{-3}$
**Sediment fluxes**				
Ksed_amm	Kinetic rate constant	41.3	41.3	$mmol\ m^{-3}$
Ksed_nit	Kinetic rate constant	73.3	73.3	$mmol\ m^{-3}$
Theta_sed_amm	Temperature dependence	1.1	1.1	-
Theta_sed_nit	Temperature dependence	1.1	1.1	-

Supplementary Table 7: Calibrated phytoplankton parameters used in the Aquatic EcoDynamics (AED) model. The default parameters are according to the AED manual.

'p_name'	cyano' (default)	'cyano' (calibrated)	'green' (default)	'green' (calibrated)	'diatom' (default)	'diatom' (calibrated)
'p_initial'	10	1	0.04	1	10	1
'p0'	0.03	0.03	0.03	0.03	0.2	0.03
'Xcc'	50	160	40	60	50	60
'R_growth'	1.1	0.8	0.7	1.7	3.1	2.7
'fT_Method'	1	1	0	1	1	1
'theta_growth'	1.06	1.08	1.08	1.08	1.08	1.08
'T_std'	20	20	20	18	18	12
'T_opt'	28	28	27	25	28	22
'T_max'	35	35	33	30	39	25
'lightModel'	0	1	0	1	0	1
'I_K'	25	10	25	10	100	5
'I_S'	100	250	100	250	40	250
'KePHY'	0.005	0.005	0.005	0.005	0.00408	0.001
'f_pr'	0.005	0.005	0.005	0.005	0.025	0.002
'R_resp'	0.08	0.08	0.012	0.012	0.02	0.12
'theta_resp'	1.05	1.05	1.06	1.06	1.08	1.07
'k_fres'	0.6	0.6	0.6	0.6	0.1	0.6
'k_fdom'	0.05	0.05	0.05	0.05	0.3	0.05
'salTol'	0	0	0	0	-2	0
'S_bep'	2	2	2	2	0.1	2
'S_maxsp'	35	35	35	35	200	35
'S_opt'	1	1	1	1	60	1
'simDINUptake'	1	1	1	1	1	1
'simDONUptake'	0	0	0	0	0	0
'simNFixation'	0	1	0	1	0	1
'simINDynamics'	2	2	2	2	0	2
'N_o'	0.25	0.25	0.25	0.25	0.02	0.25
'K_N'	4	4	3.21	3.21	1.786	3.57
'X_ncon'	0.035	0.015	0.035	0.015	0.1	0.015
'X_nmin'	0.02	0.02	0.02	0.02	0.069	0.02
'X_nmax'	0.07	0.07	0.07	0.07	0.18	0.07
'R_nuptake'	0.02	0.01	0.02	0.01	0.069	0.01
'k_nfix'	0.7	0.7	0.7	0.7	0.7	0.7
'R_nfix'	0.035	0.2	0.035	0.2	0.0006	0.2
'simDIPUptake'	1	1	1	1	1	1
'simIPDynamics'	2	1	2	1	0	1
'P_0'	0.03	0	0.03	0	0.0064	0
'K_P'	0.15	0.05	0.16	0.05	0.2426	0.05
'X_pcon'	0.002	0.0005	0.002	0.0005	0.00186	0.0005
'X_pmin'	5E-04	0.0005	5E-04	0.0005	0.0015	0.0005
'X_pmax'	0.005	0.005	0.005	0.005	0.0096	0.005
'R_puptake'	0.001	0.001	0.001	0.001	0.0031	0.001
'simSiUptake'	0	0	0	0	0	1
'Si_0'	0.3	0.3	0.3	0.3	0	0.3
'K_Si'	2.5	2.5	2.5	2.5	8	2.5
'X_sicon'	0.4	0.4	0.4	0.4	0.0171	0.4
'w_p'	0	0	-0.01	-0.01	-0.1	-0.104
'c1'	0.002	0.00207	0.002	0.002067	0	0.002067
'c3'	4E-04	0.00038	4E-04	0.000383	0	0.000383
'f1'	0.675	0.675	0.675	0.675	0	0.675
'f2'	0.75	0.75	0.75	0.75	0	0.75
'd_phy'	1.0E-5	1.0E-5	1.0E-5	1.0E-5	1.0E-5	1.0E-5

Supplementary References

1. Steinsberger, T., Schwefel, R., Wüest, A. & Müller, B. Hypolimnetic oxygen depletion rates in deep lakes: Effects of trophic state and organic matter accumulation. *Limnol. Oceanogr.* **65**, 3128–3138 (2020).
2. Fiskal, A. *et al.* Effects of Macrofaunal Recolonization on Biogeochemical Processes and Microbiota—A Mesocosm Study. *Water* vol. 13 Preprint at https://doi.org/10.3390/w13111599 (2021).
3. Fiskal, A. *et al.* Carbon sources of benthic fauna in temperate lakes across multiple trophic states. *Biogeosciences* **18**, 4369–4388 (2021).
4. Robertson, E. K. *et al.* Application of the isotope pairing technique in sediments: Use, challenges, and new directions. *Limnol. Oceanogr. Methods* **17**, 112–136 (2019).
5. Stief, P. Stimulation of microbial nitrogen cycling in aquatic ecosystems by benthic macrofauna: mechanisms and environmental implications. *Biogeosciences* **10**, 7829–7846 (2013).
6. Grant, J. R. *et al.* Proksee: in-depth characterization and visualization of bacterial genomes. *Nucleic Acids Res.* **51**, W484–W492 (2023).
7. Müller, B., Thoma, R., Baumann, K. B. L., Callbeck, C. M. & Schubert, C. J. Nitrogen removal processes in lakes of different trophic states from on-site measurements and historic data. *Aquat. Sci.* **83**, (2021).
8. Baumann, K. B. L. *et al.* Microbial Nitrogen Transformation Potential in Sediments of Two Contrasting Lakes Is Spatially Structured but Seasonally Stable. *mSphere* **0**, e01013-21 (2022).
9. Langmead, B. & Salzberg, S. L. Fast gapped-read alignment with Bowtie 2. *Nat. Methods* **9**, 357–359 (2012).
10. Manz, W., Amann, R., Ludwig, W., Vancanneyt, M. & Schleifer, K.-H. Application of a suite of 16S rRNA-specific oligonucleotide probes designed to investigate bacteria of the phylum cytophaga-flavobacter-bacteroides in the natural environment. *Microbiology (Reading).* **142 (Pt 5**, 1097–1106 (1996).
11. Ginige, M. P. *et al.* Use of stable-isotope probing, full-cycle rRNA analysis, and fluorescence in situ hybridization-microautoradiography to study a methanol-fed denitrifying microbial community. *Appl. Environ. Microbiol.* **70**, 588–596 (2004).
