# Supplementary material for: Seasonality of lake microbial denitrification and its sensitivity to climate warming
Source: Nat Microbiol. 2026 May 22;11(7):1907–19. doi: 10.1038/s41564-026-02349-9 (PMC13661275; doi:10.1038/s41564-026-02349-9)
Supplement: Supplementary file 3 [file 41564_2026_2349_MOESM3_ESM.pdf]

nature portfolio

# Peer Review File

# Seasonality of lake microbial denitrification and its sensitivity to climate warming

Corresponding Author: Dr Cameron Callbeck

Version 0:

Reviewer comments:

Reviewer #1

(Remarks to the Author)
The article is well written, concise, and easy to read. The combination of methods (isotopes to measure process rates, molecular analysis, and modeling) is rare and powerful. In fact, the clear story and straightforward writing almost masks the interdisciplinary hard work that clearly went in to the study. However, while reading the paper I kept thinking that readers of Limnology and Oceanography would appreciate it much more than readers of Nature Microbiology. I believe it is much better suited for L&O.

The hypothesized relationship between Bacteroidales members and Methylotenera is interesting and supported by data that are difficult to obtain (metagenomics in sediment and CARD-FISH are both very technologically challenging). But to really confirm this interaction, a 13C- labeled substrate (presumably chitin) experiment would be needed. This kind of confirmation (along with the data presented here) would be appropriate for Nature Micro.

Also note that much of the actual "microbiology" was previously published in Baumann et al 2022

Reviewer #2

(Remarks to the Author)
NMICROBIOL-25051631
Phenology of lake denitrification and its sensitivity to warming

Summary/Key Results: This study builds on existing research centered around the Baldeggersee lake in central Switzerland. Motivated by the prediction that climate change-induced warming will extend summer stratification and shorten the winter months during which mixing occurs, the authors wanted to understand impacts to nitrogen cycling (mostly denitrification/dissimilatory nitrate reduction to ammonium). Using a trans-disciplinary approach, the authors combined 15N-tracer assays with metagenome reconstruction and modeling to characterize seasonal dynamics and ratios between denitrification/DNRA and compared observed to modeled rates. They also centered much of this work around chitin as a potential carbon source. In general, denitrification was dominant during the mixed regime and tied strongly to POC and nitrate availability; conversely, DNRA increased in summer and late fall when denitrification decreased, likely driven by high carbon availability. Some of this denitrification, the authors speculated, could be the result of interactions between Bacteroidales and Methylonetera, who togther, can degrade chitin under oxygenated, nitrate-rich conditions. Overall, the authors stress that denitrification could decrease by 8-12% under future climate scenarios (RCP 2.6, 4.5, and 8.5) as a result of shorter mixing periods.

Overall Comments: This manuscript is well-written and combines a suite of techniques that makes for a compelling and innovative story. This narrative adds on a considerable amount of work to existing data, so please be sure to clarify what has been previously published versus what is newly presented in this manuscript (this is done well in some places already). A large portion of the story is also centered around chitin and I think the story, if space permits, would benefit from a bit more justification behind targeting that carbon source. There are some places in the methodology that could use more detail/justification – for instance, presenting nitrification potential in Fig. 1b as single cell rates/abundance versus actual measured nitrification (though perhaps I misinterpreted this) and being clear that all measurements are potential rates. Lastly, I am left wanting a bit more discussion about potential implications to water quality (e.g. if DNRA becomes more dominant over time). Overall, however, I really enjoyed this manuscript.

Specific Comments:
L61: Purely semantics, but consider finding a synonym to "furthermore."

L153: Can you further discuss the underestimation of POC flux to the sediments? (e.g. Fig. 2d)

L180: Consider providing a brief background or justification for why chitin was of interest (Text from L223 may benefit from being moved upwards in the narrative).

L201: This is certainly compelling evidence of a potential syntrophic interaction between bacteroidales and Methylotenera, but I'd also be interested in seeing a discussion, if even brief, of some other reasons these microbial groups may co-occur. For instance, they both occur at oxic-anoxic interfaces?

L209: Spelling of Methylotenera

L226: Where does this estimate come from?

L315: I'd be curious if any of these measurements were performed in triplicate? I appreciate that all data are visualized in the figures, but an indication of n/df will be helpful for context.

L519: All deposited data, intermediate files, and MAGs were located online as described in the manuscript.

Fig. 1: Is there a reason the measured potential nitrification rates from 15NH4+ were not presented here? The figure itself states "Nitrification (Predicted)," but please also make this correction in the figure caption – "Predicted sediment nitrification activity" (L787). If these are the data presented here, can this also be described in the supplemental methods? Apologies if missed.

Fig. 3b: Could benefit from slightly more detailed description about how the MAGs presented in Fig. 3b are visualized between the mixed and stratified. I think between the different conditions, presence/absence, I am losing the point of the figure just a bit.

Reviewer #3

(Remarks to the Author)
Dear Authors,

I read your article with great interest. I believe the topic is important given the current trend of many lakes toward increasingly prolonged stratification periods, which lead to the separation of surface and deep water masses. In this context, it is crucial to ask what the biogeochemical implications of such physical processes might be. In this article, the combination of experimental and modeling studies indicates that the lake in question will lose denitrification capacity in favor of increased ammonium nitrogen recycling. This is due to several factors, the most important of which appears to be the seasonal denitrification rate, which peaks during periods of full mixing—periods that are expected to become shorter. This result seems realistic and likely.

In general, the article is modern and integrates strictly experimental aspects—based both on stable isotope and molecular analyses—with a well-established model that represents physical, chemical, and biological processes in the system. The results and discussion section is well-written and generally supported by data.

The less convincing parts, in my opinion, that require further effort from the authors, concern the introduction and the materials and methods sections. While the introduction is clear and well-written, it lacks a "technical" section that describes the working hypotheses based on a solid understanding of the nitrogen biogeochemical cycle. I do not see an initial reasoning that links an expected increase in stratification period (or a reduction in full mixing duration) with the coupled dynamics of oxygen, organic matter, and dissimilatory nitrate reductions. I believe the authors are capable of including such a section, which would help formulate working hypotheses that are currently missing.

The introduction is solid, and the citations are appropriate, but both for a reader unfamiliar with the nitrogen cycle and for an expert, there is a lack of minimal guidance on the biogeochemical drivers of denitrification and DNRA, and on the relative importance of allochthonous nitrate and nitrate produced within the lake (through nitrification in water and sediments) in regulating these two processes. These aspects are well known and would allow for hypothesis generation. Without them, it appears the authors simply performed measurements and then interpreted the results afterward.

In the materials and methods section, and wherever else in the article it is relevant, it seems important to stress that the authors measured potential rather than actual activities, using a method that is not entirely clear to me. For a study of this significance, I am surprised the authors did not incubate intact sediment cores using the isotope pairing technique (IPT). It appears they added labeled nitrogen in exetainers, for a series of potential rate measurements at different sediment horizons. Moreover, the exetainers were incubated in a horizontal shaker, whereas the standard approach is to rotate them to ensure continuous sediment resuspension. It is important to understand why intact core incubations were not carried out, especially considering the study's objectives, for the reasons summarized below, which I would like the authors to address:

Slurries allow for the measurement of potential activities, which often differ significantly from actual rates. Microbial communities are placed in optimal conditions to carry out various processes. In the specific case of denitrification, many sediment bacteria have multiple possible metabolic pathways, not all of which are expressed depending on the availability of an electron acceptor such as nitrate. I believe the potential denitrification rates reported by the authors might be considerably overestimated. The same applies to potential DNRA rates. Why weren't nitrification rates measured in the same way, to obtain a complete and comparable set of bacteria-mediated processes? If these measurements are potential, what is the rationale for using them in the authors' calculations? The nitrogen cycle cannot be built on volumetric potential rate measurements. Denitrification and DNRA should be limited by the nitrate penetration into sediments, especially if—as suggested by the figure—nitrification is negligible. If the main source of nitrate for both processes is the water column, then the Den:DNRA potential ratio in the nitrification zone is

10:1.

Incubating intact cores using IPT would have allowed the authors to analyze the benthic nitrogen cycle much more accurately and with less speculation. Bioturbation processes have been extensively studied and sometimes reveal surprisingly different effects on nitrogen dynamics. For example, in Baltic Sea sediments, Marenzelleria tends to stimulate DNRA more than denitrification. With the data currently available, it is not easy to assess the role of bioturbation in the studied sediments in relation to nitrification, denitrification, and DNRA rates. With the isotope techniques used by the authors to measure potential rates, this aspect could have been easily revealed. Even more importantly, the relative contributions of nitrate reduction processes supported by nitrate from the water column versus that from nitrification in the water and sediments are unclear. With IPT, at least in sediments, processes such as Dn and Dw, rather than DNRAn and DNRAW, can be readily measured.

Regarding the above considerations, I believe it is not marginal for the authors to address the well-known effects of anoxia or hypoxia on the nitrogen cycle. In the specific case of this lake, if denitrification is mainly supported by high nitrate concentrations in the water column (as it seems), a progressive increase in stratification could lead to a decline in bioturbator biomass and reduced oxygen penetration into sediments. In this case, nitrate diffusion fluxes into the denitrification zone could become much faster, potentially causing denitrification rates to increase significantly. If, on the other hand, sediment nitrification—perhaps facilitated by bioturbators—mainly supports denitrification (though this is not evident or clear from the text), increased hypoxia or anoxia could completely inhibit this process and favor ammonium recycling, regardless of the Den:DNRA ratio. These aspects, in my opinion, are important, as they could lead to very different conclusions.

From how the methods section is written, I gather that if potential rates were measured by adding oxygen to the exetainers, the authors likely measured a mix of nitrification (which produces only $14NO3^-$), denitrification, and DNRA—thus altering the $14/15NO3^-$ ratios during incubation. This complicates calculations and the interpretation of the results. Typically, potential rates are calculated with pre-incubations long enough to deplete oxygen and $14NO3^-$, followed by the addition of $15NO3^-$. I ask the authors for clarification on this point.

I would suggest clearly stating how microbial nitrogen transformation rates (including the seasonal ones mentioned) were measured and used, and to discuss any limitations of these measurements. I also ask the authors to clarify what primarily supports denitrification and DNRA (nitrate from the water column or that produced via nitrification?).

Decision Letter:

13th August 2025

*Please ensure you delete the link to your author homepage in this e-mail if you wish to forward it to your co-authors.

Dear Cameron,

Once again, thank you for your patience while your manuscript "Phenology of lake denitrification and its sensitivity to warming" was under peer-review at Nature Microbiology. I apologize that this took quite so long--as I said, because of the time of year, it was very hard to secure referees. Nevertheless, your work has now been seen by 3 referees, whose expertise and comments you will find at the end of this email. Given the delay, I'm pleased to get back to you with some encouraging news: they find your work of considerable potential interest...though they have also raised a number of concerns that will need to be addressed before we can consider publication of the work in Nature Microbiology.

As you will see, most of the concerns are editorial and should be straightforward to address. (As an aside, don't worry about what Reviewer #1 says about L&O...we disagree, and think the work would make a good fit at Nature Micro, or else we would not have sent it out to review!) The biggest technical concern comes from Reviewer #3, regarding the incubation experiments--their concerns will either need to be addressed with additional experiments and/or analyses, or much stronger justification for your experimental choices.

Should further experimental data allow you to address these criticisms, we would be happy to look at a revised manuscript. Happy to discuss a revision plan if you'd like and you think it would be helpful!

We are committed to providing a fair and constructive peer-review process. Please do not hesitate to contact us if there are specific requests from the reviewers that you believe are technically impossible or unlikely to yield a meaningful outcome.

We strongly support public availability of data. Please place the data used in your paper into a public data repository, if one exists, or alternatively, present the data as Source Data or Supplementary Information. If data can only be shared on request, please explain why in your Data Availability Statement, and also in the correspondence with your editor. For some data types, deposition in a public repository is mandatory - more information on our data deposition policies and available repositories can be found at https://www.nature.com/nature-research/editorial-policies/reporting-standards#availability-of-data.

Please include a data availability statement as a separate section after Methods but before references, under the heading "Data Availability". This section should inform readers about the availability of the data used to support the conclusions of your study. This information includes accession codes to public repositories (data banks for protein, DNA or RNA sequences, microarray, proteomics data etc…), references to source data published alongside the paper, unique identifiers such as URLs to data repository entries, or data set DOIs, and any other statement about data availability. At a minimum, you should include the following statement: “The data that support the findings of this study are available from the corresponding author upon request”,

mentioning any restrictions on availability. If DOIs are provided, we also strongly encourage including these in the Reference list (authors, title, publisher (repository name), identifier, year). For more guidance on how to write this section please see: http://www.nature.com/authors/policies/data/data-availability-statements-data-citations.pdf

If revising your manuscript:

* Include a "Response to referees" document detailing, point-by-point, how you addressed each referee comment. If no action was taken to address a point, you must provide a compelling argument. This response will be sent back to the referees along with the revised manuscript.

* If you have not done so already we suggest that you begin to revise your manuscript so that it conforms to our Article format instructions at http://www.nature.com/nmicrobiol/info/final-submission. Refer also to any guidelines provided in this letter.

* Include a revised version of any required reporting checklist. It will be available to referees (and, potentially, statisticians) to aid in their evaluation if the manuscript goes back for peer review. A revised checklist is essential for re-review of the paper.

When submitting the revised version of your manuscript, please pay close attention to our href="https://www.nature.com/nature-portfolio/editorial-policies/image-integrity">Digital Image Integrity Guidelines.</a> and to the following points below:

-- that unprocessed scans are clearly labelled and match the gels and western blots presented in figures.
-- that control panels for gels and western blots are appropriately described as loading on sample processing controls
-- all images in the paper are checked for duplication of panels and for splicing of gel lanes.

EXTENDED DATA FIGURES

When re-submitting your manuscript, please ensure that any supplementary figures and tables that are crucial to the manuscript's conclusions are converted into Extended Data figures and tables to increase visibility of these data. Extended Data figures and tables are online-only (present in the online PDF and full-text HTML versions of the paper), peer-reviewed display items that provide essential background to the article but are not included in the main article due to space constraints. A maximum of ten Extended Data display items (figures and tables) is permitted.

Finally, please ensure that you retain unprocessed data and metadata files after publication, ideally archiving data in perpetuity, as these may be requested during the peer review and production process or after publication if any issues arise.

Please use the link below to submit a revised paper:

Link Redacted

**Note:** This url links to your confidential homepage and associated information about manuscripts you may have submitted or be reviewing for us. If you wish to forward this e-mail to co-authors, please delete this link to your homepage first.

Nature Microbiology is committed to improving transparency in authorship. As part of our efforts in this direction, we are now requesting that all authors identified as 'corresponding author' on published papers create and link their Open Researcher and Contributor Identifier (ORCID) with their account on the Manuscript Tracking System (MTS), prior to acceptance. This applies to primary research papers only. ORCID helps the scientific community achieve unambiguous attribution of all scholarly contributions. You can create and link your ORCID from the home page of the MTS by clicking on 'Modify my Springer Nature account'. For more information please visit please visit <a href="http://www.springernature.com/orcid">www.springernature.com/orcid</a>.

If you wish to submit a suitably revised manuscript we would hope to receive it within 6 months. If you cannot send it within this time, please let us know. We will be happy to consider your revision, even if a similar study has been accepted for publication at Nature Microbiology or published elsewhere (up to a maximum of 6 months).

In the meantime we hope that you find our referees' comments helpful.

Yours sincerely,

****************************************************

Reviewer Expertise:

Referee #1: freshwater microbiology and biogeochem, genomics
Referee #2: freshwater biogeochem, microbial ecology
Referee #3: freshwater biogeochem, isotopic experiments

Reviewer Comments:

Reviewer #1 (Remarks to the Author):

The article is well written, concise, and easy to read. The combination of methods (isotopes to measure process rates, molecular analysis, and modeling) is rare and powerful. In fact, the clear story and straightforward writing almost masks the interdisciplinary hard work that clearly went in to the study. However, while reading the paper I kept thinking that readers of Limnology and Oceanography would appreciate it much more than readers of Nature Microbiology. I believe it is much better suited for L&O.

The hypothesized relationship between Bacteroidales members and Methylotenera is interesting and supported by data that are difficult to obtain (metagenomics in sediment and CARD-FISH are both very technologically challenging). But to really confirm this interaction, a 13C- labeled substrate (presumably chitin) experiment would be needed. This kind of confirmation (along with the data presented here) would be appropriate for Nature Micro.

Also note that much of the actual "microbiology" was previously published in Baumann et al 2022

Reviewer #2 (Remarks to the Author):

NMICROBIOL-25051631
Phenology of lake denitrification and its sensitivity to warming

Summary/Key Results: This study builds on existing research centered around the Baldeggersee lake in central Switzerland. Motivated by the prediction that climate change-induced warming will extend summer stratification and shorten the winter months during which mixing occurs, the authors wanted to understand impacts to nitrogen cycling (mostly denitrification/dissimilatory nitrate reduction to ammonium). Using a trans-disciplinary approach, the authors combined 15N-tracer assays with metagenome reconstruction and modeling to characterize seasonal dynamics and ratios between denitrification/DNRA and compared observed to modeled rates. They also centered much of this work around chitin as a potential carbon source. In general, denitrification was dominant during the mixed regime and tied strongly to POC and nitrate availability; conversely, DNRA increased in summer and late fall when denitrification decreased, likely driven by high carbon availability. Some of this denitrification, the authors speculated, could be the result of interactions between Bacteroidales and Methylonetera, who togther, can degrade chitin under oxygenated, nitrate-rich conditions. Overall, the authors stress that denitrification could decrease by 8-12% under future climate scenarios (RCP 2.6, 4.5, and 8.5) as a result of shorter mixing periods.

Overall Comments: This manuscript is well-written and combines a suite of techniques that makes for a compelling and innovative story. This narrative adds on a considerable amount of work to existing data, so please be sure to clarify what has been previously published versus what is newly presented in this manuscript (this is done well in some places already). A large portion of the story is also centered around chitin and I think the story, if space permits, would benefit from a bit more justification behind targeting that carbon source. There are some places in the methodology that could use more detail/justification – for instance, presenting nitrification potential in Fig. 1b as single cell rates/abundance versus actual measured nitrification (though perhaps I misinterpreted this) and being clear that all measurements are potential rates. Lastly, I am left wanting a bit more discussion about potential implications to water quality (e.g. if DNRA becomes more dominant over time). Overall, however, I really enjoyed this manuscript.

Specific Comments:
L61: Purely semantics, but consider finding a synonym to "furthermore."

L153: Can you further discuss the underestimation of POC flux to the sediments? (e.g. Fig. 2d)

L180: Consider providing a brief background or justification for why chitin was of interest (Text from L223 may benefit from being moved upwards in the narrative).

L201: This is certainly compelling evidence of a potential syntrophic interaction between bacteroidales and Methylotenera, but I'd also be interested in seeing a discussion, if even brief, of some other reasons these microbial groups may co-occur. For instance, they both occur at oxic-anoxic interfaces?

L209: Spelling of Methylotenera

L226: Where does this estimate come from?

L315: I'd be curious if any of these measurements were performed in triplicate? I appreciate that all data are visualized in the figures, but an indication of n/df will be helpful for context.

L519: All deposited data, intermediate files, and MAGs were located online as described in the manuscript.

Fig. 1: Is there a reason the measured potential nitrification rates from 15NH4+ were not presented here? The figure itself states "Nitrification (Predicted)," but please also make this correction in the figure caption – "Predicted sediment nitrification activity" (L787). If these are the data presented here, can this also be described in the supplemental methods? Apologies if missed.

Fig. 3b: Could benefit from slightly more detailed description about how the MAGs presented in Fig. 3b are visualized between the mixed and stratified. I think between the different conditions, presence/absence, I am losing the point of the figure just a bit.

Reviewer #3 (Remarks to the Author):

Dear Authors,

I read your article with great interest. I believe the topic is important given the current trend of many lakes toward increasingly prolonged stratification periods, which lead to the separation of surface and deep water masses. In this context, it is crucial to ask what the biogeochemical implications of such physical processes might be. In this article, the combination of experimental and modeling studies indicates that the lake in question will lose denitrification capacity in favor of increased ammonium nitrogen recycling. This is due to several factors, the most important of which appears to be the seasonal denitrification rate, which peaks during periods of full mixing—periods that are expected to become shorter. This result seems realistic and likely.

In general, the article is modern and integrates strictly experimental aspects—based both on stable isotope and molecular analyses—with a well-established model that represents physical, chemical, and biological processes in the system. The results and discussion section is well-written and generally supported by data.

The less convincing parts, in my opinion, that require further effort from the authors, concern the introduction and the materials and methods sections. While the introduction is clear and well-written, it lacks a "technical" section that describes the working hypotheses based on a solid understanding of the nitrogen biogeochemical cycle. I do not see an initial reasoning that links an expected increase in stratification period (or a reduction in full mixing duration) with the coupled dynamics of oxygen, organic matter, and dissimilatory nitrate reductions. I believe the authors are capable of including such a section, which would help formulate working hypotheses that are currently missing.

The introduction is solid, and the citations are appropriate, but both for a reader unfamiliar with the nitrogen cycle and for an expert, there is a lack of minimal guidance on the biogeochemical drivers of denitrification and DNRA, and on the relative importance of allochthonous nitrate and nitrate produced within the lake (through nitrification in water and sediments) in regulating these two processes. These aspects are well known and would allow for hypothesis generation. Without them, it appears the authors simply performed measurements and then interpreted the results afterward.

In the materials and methods section, and wherever else in the article it is relevant, it seems important to stress that the authors measured potential rather than actual activities, using a method that is not entirely clear to me. For a study of this significance, I am surprised the authors did not incubate intact sediment cores using the isotope pairing technique (IPT). It appears they added labeled nitrogen in exetainers, for a series of potential rate measurements at different sediment horizons. Moreover, the exetainers were incubated in a horizontal shaker, whereas the standard approach is to rotate them to ensure continuous sediment resuspension. It is important to understand why intact core incubations were not carried out, especially considering the study's objectives, for the reasons summarized below, which I would like the authors to address:

Slurries allow for the measurement of potential activities, which often differ significantly from actual rates. Microbial communities are placed in optimal conditions to carry out various processes. In the specific case of denitrification, many sediment bacteria have multiple possible metabolic pathways, not all of which are expressed depending on the availability of an electron acceptor such as nitrate. I believe the potential denitrification rates reported by the authors might be considerably overestimated. The same applies to potential DNRA rates. Why weren't nitrification rates measured in the same way, to obtain a complete and comparable set of bacteria-mediated processes? If these measurements are potential, what is the rationale for using them in the authors' calculations? The nitrogen cycle cannot be built on volumetric potential rate measurements. Denitrification and DNRA should be limited by the nitrate penetration into sediments, especially if—as suggested by the figure—nitrification is negligible. If the main source of nitrate for both processes is the water column, then the Den:DNRA potential ratio in the nitrification zone is 10:1.

Incubating intact cores using IPT would have allowed the authors to analyze the benthic nitrogen cycle much more accurately and with less speculation. Bioturbation processes have been extensively studied and sometimes reveal surprisingly different effects on nitrogen dynamics. For example, in Baltic Sea sediments, Marenzelleria tends to stimulate DNRA more than denitrification. With the data currently available, it is not easy to assess the role of bioturbation in the studied sediments in relation to nitrification, denitrification, and DNRA rates. With the isotope techniques used by the authors to measure potential rates, this aspect could have been easily revealed. Even more importantly, the relative contributions of nitrate reduction processes supported by nitrate from the water column versus that from nitrification in the water and sediments are unclear. With IPT, at least in sediments, processes such as Dn and Dw, rather than DNRAn and DNRAW, can be readily measured.

Regarding the above considerations, I believe it is not marginal for the authors to address the well-known effects of anoxia or hypoxia on the nitrogen cycle. In the specific case of this lake, if denitrification is mainly supported by high nitrate concentrations in the water column (as it seems), a progressive increase in stratification could lead to a decline in bioturbator biomass and reduced oxygen penetration into sediments. In this case, nitrate diffusion fluxes into the denitrification zone could become much faster, potentially causing denitrification rates to increase significantly. If, on the other hand, sediment nitrification—perhaps facilitated by bioturbators—mainly supports denitrification (though this is not evident or clear from the text), increased hypoxia or anoxia could completely inhibit this process and favor ammonium recycling, regardless of the Den:DNRA ratio. These aspects, in my opinion, are important, as they could lead to very different conclusions.

From how the methods section is written, I gather that if potential rates were measured by adding oxygen to the exetainers, the authors likely measured a mix of nitrification (which produces only $^{14}NO_3^-$), denitrification, and DNRA—thus altering the $^{14/15}NO_3^-$ ratios during incubation. This complicates calculations and the interpretation of the results. Typically, potential rates

are calculated with pre-incubations long enough to deplete oxygen and $^{14}NO_3^-$, followed by the addition of $^{15}NO_3^-$. I ask the authors for clarification on this point.

I would suggest clearly stating how microbial nitrogen transformation rates (including the seasonal ones mentioned) were measured and used, and to discuss any limitations of these measurements. I also ask the authors to clarify what primarily supports denitrification and DNRA (nitrate from the water column or that produced via nitrification?).

******************************************************

Version 1:

Reviewer comments:

Reviewer #3

(Remarks to the Author)
Dear authors
thanks for addressing in the revised version of the manuscript my comments to the original version. I am in general happy with the way you improved the manuscript. I think there is room for future methodological improvements to turn your potential rates into in situ ones. I know that bioturbation makes the application of the IPT problematic, but there are a lot of good papers addressing benthic N cycling in bioturbated sediments, performed with combinations of net fluxes and r-IPT. I am surprised that with such high density of bioturbators potential rates of sediment nitrification are so low. $NH_4^+$ and $O_2$ should not be limiting in such conditions and a large stimulation of nitrification is generally found.

Reviewer #4

(Remarks to the Author)
The Aquatic EcoDynamics (AED) model is a decent tool capable of simulating phytoplankton growth under varying environmental conditions, including temperature, light, and nutrient availability. The model appropriately represents the transformation of fixed carbon into POC through direct phytoplankton mortality and indirect pathways via zooplankton and bivalve grazing.
However, Table S7 appears to duplicate the content of Table S6, which makes it difficult to evaluate the results without the specific parameters used to calibrate phytoplankton growth.
In general, modeled POC fluxes in aquatic systems are sometimes underestimated, typically due to unaccounted lateral transport or other physical processes. A moderate bias is therefore understandable and can be justified. In this study, however, the modeled POC fluxes are underestimated by more than an order of magnitude, particularly under mixed-regime conditions. This large discrepancy leads to an artificially significant difference in modeled POC flux and Rpoc:no3 between the stratified and mixed regimes, a pattern that is not evident in the observational data (Fig. 2c, i). The same issue is also evident for modeled $O_2$ fluxes.
Given that the modeled and observed POC fluxes (and Rpoc:no3) differ by more than two orders of magnitude, a direct comparison between the two datasets is not meaningful.
Furthermore, the modeled POC fluxes show extremely high variability (Fig. 2c, d), which in turn dominates the variability in the modeled Rpoc:no3.
Because the observed POC flux are usually much higher and exhibit much smaller variation than the modeled values, it is possible that a relative steady lateral transport constitutes the dominate source of POC flux in the lake.
If lateral transport and other unrepresented processes indeed dominate POC flux dynamics in the lake, then the model's attribution of POC flux variation to seasonal hydrological changes, and the consequent effects on denitrification and DNRA, may not be valid. In that case, the argument linking seasonal variation to these biogeochemical processes, as well as the future projections based on it, lacks sufficient support.
To clarify those concerns, it would be useful if the authors could provide details explaining why the modeled POC fluxes are substantially lower and exhibit considerably higher variation than the observations, as well as the large temporal offset (2 months in POC flux). Nevertheless, the related argument concerning the relationship between denitrification and DNRA remains unsupported if the modeled results can not be significantly improved through parameter optimisation.

Decision Letter:

1st December 2025

*Please ensure you delete the link to your author homepage in this e-mail if you wish to forward it to your co-authors.

Dear Cameron,

Thank you for your patience while your manuscript "Phenology of lake denitrification and its sensitivity to warming" was under peer-review again at Nature Microbiology. It has now been seen by 2 referees, whose expertise and comments you will find at

the end of this email. We returned this to Reviewer #3 from the previous round, and they are satisfied with the revisions which is great. Unfortunately, neither Reviewer #1 or #2 were available to review the work again, so we recruited a new reviewer, Reviewer #4. Although this reviewer also finds your work of some potential interest, they have raised a number of concerns with the model parameters that will need to be addressed before we can consider publication of the work in Nature Microbiology.

In particular, Reviewer #4 points out that your modeled POC fluxes are underestimated by more than an order of magnitude, and they are concerned that this could lead to an artificially significant difference in modeled POC fluxes between the two lake conditions, and that this does not correlate with the actual observed data from the lake. Should further experimental data allow you to address these issues with model parameterization (and with no major impact on the main conclusions of the work), we would be happy to look at a revised manuscript.

We are committed to providing a fair and constructive peer-review process. Please do not hesitate to contact us if there are specific requests from the reviewers that you believe are technically impossible or unlikely to yield a meaningful outcome.

We strongly support public availability of data. Please place the data used in your paper into a public data repository, if one exists, or alternatively, present the data as Source Data or Supplementary Information. If data can only be shared on request, please explain why in your Data Availability Statement, and also in the correspondence with your editor. For some data types, deposition in a public repository is mandatory - more information on our data deposition policies and available repositories can be found at https://www.nature.com/nature-research/editorial-policies/reporting-standards#availability-of-data.

Please include a data availability statement as a separate section after Methods but before references, under the heading "Data Availability". This section should inform readers about the availability of the data used to support the conclusions of your study. This information includes accession codes to public repositories (data banks for protein, DNA or RNA sequences, microarray, proteomics data etc…), references to source data published alongside the paper, unique identifiers such as URLs to data repository entries, or data set DOIs, and any other statement about data availability. At a minimum, you should include the following statement: "The data that support the findings of this study are available from the corresponding author upon request", mentioning any restrictions on availability. If DOIs are provided, we also strongly encourage including these in the Reference list (authors, title, publisher (repository name), identifier, year). For more guidance on how to write this section please see: http://www.nature.com/authors/policies/data/data-availability-statements-data-citations.pdf

If revising your manuscript:

* Include a "Response to referees" document detailing, point-by-point, how you addressed each referee comment. If no action was taken to address a point, you must provide a compelling argument. This response will be sent back to the referees along with the revised manuscript.

* If you have not done so already we suggest that you begin to revise your manuscript so that it conforms to our Article format instructions at http://www.nature.com/nmicrobiol/info/final-submission. Refer also to any guidelines provided in this letter.

* Include a revised version of any required reporting checklist. It will be available to referees (and, potentially, statisticians) to aid in their evaluation if the manuscript goes back for peer review. A revised checklist is essential for re-review of the paper.

When submitting the revised version of your manuscript, please pay close attention to our href="https://www.nature.com/nature-portfolio/editorial-policies/image-integrity">Digital Image Integrity Guidelines.</a> and to the following points below:

-- that unprocessed scans are clearly labelled and match the gels and western blots presented in figures.
-- that control panels for gels and western blots are appropriately described as loading on sample processing controls
-- all images in the paper are checked for duplication of panels and for splicing of gel lanes.

EXTENDED DATA FIGURES

When re-submitting your manuscript, please ensure that any supplementary figures and tables that are crucial to the manuscript's conclusions are converted into Extended Data figures and tables to increase visibility of these data. Extended Data figures and tables are online-only (present in the online PDF and full-text HTML versions of the paper), peer-reviewed display items that provide essential background to the article but are not included in the main article due to space constraints. A maximum of ten Extended Data display items (figures and tables) is permitted.

Finally, please ensure that you retain unprocessed data and metadata files after publication, ideally archiving data in perpetuity, as these may be requested during the peer review and production process or after publication if any issues arise.

Please use the link below to submit a revised paper:

Link Redacted

**Note:** This url links to your confidential homepage and associated information about manuscripts you may have submitted or be reviewing for us. If you wish to forward this e-mail to co-authors, please delete this link to your homepage first.

Nature Microbiology is committed to improving transparency in authorship. As part of our efforts in this direction, we are now

requesting that all authors identified as ‘corresponding author’ on published papers create and link their Open Researcher and Contributor Identifier (ORCID) with their account on the Manuscript Tracking System (MTS), prior to acceptance. This applies to primary research papers only. ORCID helps the scientific community achieve unambiguous attribution of all scholarly contributions. You can create and link your ORCID from the home page of the MTS by clicking on ‘Modify my Springer Nature account’. For more information please visit please visit <a href="http://www.springernature.com/orcid">www.springernature.com/orcid</a>.

If you wish to submit a suitably revised manuscript we would hope to receive it within 6 months. If you cannot send it within this time, please let us know. We will be happy to consider your revision, even if a similar study has been accepted for publication at Nature Microbiology or published elsewhere (up to a maximum of 6 months).

In the meantime we hope that you find our referees' comments helpful.

Yours sincerely,

*****************************************************

Reviewer Expertise:

Referee #3: freshwater biogeochem, isotopic experiments
Referee #4: climate modeling, lake biogeochemistry

Reviewer Comments:

Reviewer #3 (Remarks to the Author):

Dear authors
thanks for addressing in the revised version of the manuscript my comments to the original version. I am in general happy with the way you improved the manuscript. I think there is room for future methodological improvements to turn your potential rates into in situ ones. I know that bioturbation makes the application of the IPT problematic, but there are a lot of good papers addressing benthic N cycling in bioturbated sediments, performed with combinations of net fluxes and r-IPT. I am surprised that with such high density of bioturbators potential rates of sediment nitrification are so low. NH4+ and O2 should not be limiting in such conditions and a large stimulation of nitrification is generally found.

Reviewer #4 (Remarks to the Author):

The Aquatic EcoDynamics (AED) model is a decent tool capable of simulating phytoplankton growth under varying environmental conditions, including temperature, light, and nutrient availability. The model appropriately represents the transformation of fixed carbon into POC through direct phytoplankton mortality and indirect pathways via zooplankton and bivalve grazing.
However, Table S7 appears to duplicate the content of Table S6, which makes it difficult to evaluate the results without the specific parameters used to calibrate phytoplankton growth.
In general, modeled POC fluxes in aquatic systems are sometimes underestimated, typically due to unaccounted lateral transport or other physical processes. A moderate bias is therefore understandable and can be justified. In this study, however, the modeled POC fluxes are underestimated by more than an order of magnitude, particularly under mixed-regime conditions. This large discrepancy leads to an artificially significant difference in modeled POC flux and Rpoc:no3 between the stratified and mixed regimes, a pattern that is not evident in the observational data (Fig. 2c, i). The same issue is also evident for modeled $O_2$ fluxes.
Given that the modeled and observed POC fluxes (and Rpoc:no3) differ by more than two orders of magnitude, a direct comparison between the two datasets is not meaningful.
Furthermore, the modeled POC fluxes show extremely high variability (Fig. 2c, d), which in turn dominates the variability in the modeled Rpoc:no3.
Because the observed POC flux are usually much higher and exhibit much smaller variation than the modeled values, it is possible that a relative steady lateral transport constitutes the dominate source of POC flux in the lake.
If lateral transport and other unrepresented processes indeed dominate POC flux dynamics in the lake, then the model’s attribution of POC flux variation to seasonal hydrological changes, and the consequent effects on denitrification and DNRA, may not be valid. In that case, the argument linking seasonal variation to these biogeochemical processes, as well as the future projections based on it, lacks sufficient support.
To clarify those concerns, it would be useful if the authors could provide details explaining why the modeled POC fluxes are substantially lower and exhibit considerably higher variation than the observations, as well as the large temporal offset (2 months in POC flux). Nevertheless, the related argument concerning the relationship between denitrification and DNRA remains unsupported if the modeled results can not be significantly improved through parameter optimisation.

*****************************************************

Version 2:

Reviewer comments:

Reviewer #4

(Remarks to the Author)
I would like to thank the authors for addressing my previous comments regarding the mismatch between the observed and modeled POC fluxes (and other parameters) by introducing the refocusing model. The new model scheme greatly improves the results and supports the conclusion that refocused/lateral POC contributes substantially to the total flux to the sediment.

I am generally satisfied with the revised manuscript. However, it would be helpful if the authors could briefly discuss the artificially significant difference in the modeled oxygen flux between the stratified and mixed periods, and address how this might affect other model outcomes.

Decision Letter:

Our ref: NMICROBIOL-25051631B

23rd February 2026

Dear Cameron,

I hope you're doing well. Some good news...at long last! Thanks for the patience, and thank you for submitting your revised manuscript "Phenology of lake denitrification and its sensitivity to warming" (NMICROBIOL-25051631B). It has now been seen by the original referees and their comments are below. The reviewers find that the paper has improved in revision, and therefore we'll be happy in principle to publish it in Nature Microbiology, pending minor revisions to satisfy the referees' final requests and to comply with our editorial and formatting guidelines.

If the current version of your manuscript is in a PDF format, please email us a copy of the file in an editable format (Microsoft Word or LaTex)-- we can not proceed with PDFs at this stage.

We are now performing detailed checks on your paper and will send you a checklist detailing our editorial and formatting requirements in about a week. Please do not upload the final materials and make any revisions until you receive this additional information from us.

Thank you again for your interest in Nature Microbiology Please do not hesitate to contact me if you have any questions.

Sincerely,

Reviewer #4 (Remarks to the Author):

I would like to thank the authors for addressing my previous comments regarding the mismatch between the observed and modeled POC fluxes (and other parameters) by introducing the refocusing model. The new model scheme greatly improves the results and supports the conclusion that refocused/lateral POC contributes substantially to the total flux to the sediment.

I am generally satisfied with the revised manuscript. However, it would be helpful if the authors could briefly discuss the artificially significant difference in the modeled oxygen flux between the stratified and mixed periods, and address how this might affect other model outcomes.

Version 3:

Decision Letter:

8th April 2026

Dear Dr Callbeck,

I am pleased to accept your Article "Seasonality of lake microbial denitrification and its sensitivity to climate warming" for publication in Nature Microbiology. Thank you for having chosen to submit your work to us and many congratulations.

I saw you uploaded a cover option--could you provide some more details on what the picture depicts?

Over the next few weeks, your paper will be copyedited to ensure that it conforms to Nature Microbiology style. We look particularly carefully at the titles of all papers to ensure that they are relatively brief and understandable.

Once your paper is typeset, you will receive an email with a link to choose the appropriate publishing options for your paper and our Author Services team will be in touch regarding any additional information that may be required. Once your paper has been scheduled for online publication, the Nature press office will be in touch to confirm the details.

You may wish to make your media relations office aware of your accepted publication, in case they consider it appropriate to organize some internal or external publicity. Once your paper has been scheduled you will receive an email confirming the publication details. This is normally 3-4 working days in advance of publication. If you need additional notice of the date and time of publication, please let the production team know when you receive the proof of your article to ensure there is sufficient time to coordinate. Further information on our embargo policies can be found here:
https://www.nature.com/authors/policies/embargo.html

After the grant of rights is completed, you will receive a link to your electronic proof via email with a request to make any corrections within 48 hours. If, when you receive your proof, you cannot meet this deadline, please inform us at rjsproduction@springernature.com immediately. You will not receive your proofs until the publishing agreement has been received through our system

Due to the importance of these deadlines, we ask you please us know now whether you will be difficult to contact over the next month. If this is the case, we ask you provide us with the contact information (email, phone and fax) of someone who will be able to check the proofs on your behalf, and who will be available to address any last-minute problems.

Acceptance of your manuscript is conditional on all authors' agreement with our publication policies (see https://www.nature.com/nmicrobiol/editorial-policies). In particular your manuscript must not be published elsewhere.

**Authors may need to take specific actions to achieve compliance with funder and institutional open access mandates.** If your research is supported by a funder that requires immediate open access (e.g. according to <a href="https://www.springernature.com/gp/open-science/plan-s-compliance"> Plan S principles</a> or the <a href="https://www.springernature.com/gp/open-science/us-federal-agency-compliance"> NIH public access policy</a>) then you should select the gold OA route, and we will direct you to the compliant route where possible. Because authors warrant under our subscription licensing terms that they haven't committed to licensing any version of their article under a licence inconsistent with the terms of our agreement – including the applicable embargo period – publication under the subscription model isn't suitable for authors whose funders require no embargo.

If you have any questions about our publishing options, costs, Open Access requirements, or our legal forms, please contact ASJournals@springernature.com

An online order form for reprints of your paper is available at <a href="https://www.nature.com/reprints/author-reprints.html">https://www.nature.com/reprints/author-reprints.html</a>. All co-authors, authors' institutions and authors' funding agencies can order reprints using the form appropriate to their geographical region.

We welcome the submission of potential cover material (including a short caption of around 40 words) related to your manuscript; suggestions should be sent to Nature Microbiology as electronic files (the image should be 300 dpi at 210 x 297 mm in either TIFF or JPEG format). Please note that such pictures should be selected more for their aesthetic appeal than for their scientific content, and that colour images work better than black and white or grayscale images. Please do not try to design a cover with the Nature Microbiology logo etc., and please do not submit composites of images related to your work. I am sure you will understand that we cannot make any promise as to whether any of your suggestions might be selected for the cover of the journal.

You can now use a single sign-on for all your accounts, view the status of all your manuscript submissions and reviews, access usage statistics for your published articles and download a record of your refereeing activity for the Nature journals.

To assist our authors in disseminating their research to the broader community, our SharedIt initiative provides you with a unique shareable link that will allow anyone (with or without a subscription) to read the published article. Recipients of the link with a subscription will also be able to download and print the PDF.

As soon as your article is published, you will receive an automated email with your shareable link.

With kind regards,

P.S. Click on the following link if you would like to recommend Nature Microbiology to your librarian
http://www.nature.com/subscriptions/recommend.html#forms

** Visit the Springer Nature Editorial and Publishing website at <a href="http://editorial-jobs.springernature.com?utm_source=ejP_NMicro_email&utm_medium=ejP_NMicro_email&utm_campaign=ejp_NMicro">www.springernature.com/editorial-

and-publishing-jobs</a> for more information about our career opportunities. If you have any questions please click <a href="mailto:editorial.publishing.jobs@springernature.com">here</a>.**

**Open Access** This Peer Review File is licensed under a Creative Commons Attribution 4.0 International License, which permits use, sharing, adaptation, distribution and reproduction in any medium or format, as long as you give appropriate credit to the original author(s) and the source, provide a link to the Creative Commons license, and indicate if changes were made.
In cases where reviewers are anonymous, credit should be given to 'Anonymous Referee' and the source.
The images or other third party material in this Peer Review File are included in the article's Creative Commons license, unless indicated otherwise in a credit line to the material. If material is not included in the article's Creative Commons license and your intended use is not permitted by statutory regulation or exceeds the permitted use, you will need to obtain permission directly from the copyright holder.
To view a copy of this license, visit https://creativecommons.org/licenses/by/4.0/

We greatly appreciate the three reviewers for their support of this work, their attention to detail, and their constructive criticism. Below is a point-by-point response to the reviewers' comments, indicated in the red-type face. The grey highlighted line numbers refer to changes made in the revised manuscript (e.g lines XX-XX). A complete list of references is included at the end of the response to reviewers' comments.

Reviewer Comments:

Reviewer #1 (Remarks to the Author):

The article is well written, concise, and easy to read. The combination of methods (isotopes to measure process rates, molecular analysis, and modeling) is rare and powerful. In fact, the clear story and straightforward writing almost masks the interdisciplinary hard work that clearly went in to the study. However, while reading the paper I kept thinking that readers of Limnology and Oceanography would appreciate it much more than readers of Nature Microbiology. I believe it is much better suited for L&O.

We thank the reviewer for recognizing the interdisciplinary nature of this work. Our work not only advances our understanding of the nitrogen cycle and its interactions with other biogeochemical cycles, but it also offers forward-looking insights into how the key microbial process of denitrification, and the associated microbes, may respond to climate warming. We agree that readers of *Limnology & Oceanography* would value the work; however, we contend the paper is well-suited to *Nature Microbiology* precisely because of its microbe-centric, mechanistic contributions enabled by the "rare and powerful" integration of techniques the reviewer highlights.

The hypothesized relationship between Bacteroidales members and Methylotenera is interesting and supported by data that are difficult to obtain (metagenomics in sediment and CARD-FISH are both very technologically challenging). But to really confirm this interaction, a 13C- labeled substrate (presumably chitin) experiment would be needed. This kind of confirmation (along with the data presented here) would be appropriate for Nature Micro.

Thank you for this thoughtful suggestion. We agree that a $^{13}$C-labeled substrate SIP experiment (e.g., $^{13}$C-chitin/pectin) would provide a good test of the proposed Bacteroidales-Methylotenera interaction. However, a potential challenge lies in the slow degradation of chitin and the requirement for extracellular enzymatic activity, which could result in broad uptake of the released $^{13}$C-labeled monomers and subsequent cross-feeding within the microbial community. As such, it is unclear whether this approach (i.e., $^{13}$C-labeled chitin coupled with SIP experiments) would yield clearer insights into the primary degraders. However, even in the absence of performing $^{13}$C-labeled chitin/pectin experiments, the present study provides three independent lines of evidence to suggest a potential partnership between Bacteroidales and Methylotenera:

1. Mechanistic plausibility: genome-informed metabolic models indicate that Bacteroidales degradation of chitin/pectin can release methanol and $NH_4^+$, substrates predicted to fuel *Methylotenera* denitrification (Fig. 3c).
2. Spatial co-occurrence: CARD-FISH enumeration shows Bacteroidales and Methylophilales (the order containing *Methylotenera*) co-located in the chitin/pectin-rich upper sediments (Fig. 1c).
3. And most compelling is the seasonal coupling: across the annual cycle, *Methylotenera* abundance is positively associated with pectinolytic/chitinolytic Bacteroidales (Extended Data Fig. 5c; Pearson correlation, two-tailed $p < 0.01$; df = 12; $\alpha = 0.05$). Moreover, the proposed Bacteroidales-*Methylotenera* consortium reached its highest abundance in the late-mixed/early spring period, coinciding with the peak in chitin inventories and the fluxes of oxygen and nitrate into the sediments (Fig. 2d, f, h).

As Reviewer 2 noted, this constitutes "compelling evidence of a potential syntrophic interaction." While we agree that $^{13}$C-SIP incubations would further test the Bacteroidales-Methylotenera partnership, we consider the current evidence sufficient to justify describing it as a "potential" partnership, which is now clarified in the manuscript (e.g., see line 19). We also note that omics-based inference of interactions is common practice; here, it primarily provides a mechanistic context for our central contribution, which is to resolve denitrification phenology and warming sensitivity using isotopes, molecular approaches, and ecosystem modelling.

Also note that much of the actual "microbiology" was previously published in Baumann et al 2022

The reviewer is correct that *Baumann et al.* (2022) provided an important first step toward understanding the microbially driven nitrogen cycle across lakes of varying trophic states. While the molecular/microbial aspect is one-third of our manuscript (alongside ecosystem modelling and rate transformation processes), our study complements and extends that microbiology work in three ways:

1. Taxon focus: We quantify the relative abundances of two key taxa that were not the focus of *Baumann et al.* (2022).
2. Genome resolution: We conduct MAG binning to resolve population genomes and to understand the interaction of two putative partnerships, thereby providing a more refined, genome-resolved view than the cross-lake synthesis in *Baumann et al.* (2022).
3. Seasonal dynamics: We use read mapping to link community composition to seasonal trends, which were not a central focus of *Baumann et al.* (2022).

As Reviewer 2 noted, "This narrative adds on a considerable amount of work to existing data…".

Reviewer #2 (Remarks to the Author):

NMICROBIOL-25051631
Phenology of lake denitrification and its sensitivity to warming

Summary/Key Results: This study builds on existing research centered around the Baldeggersee lake in central Switzerland. Motivated by the prediction that climate change-induced warming will extend summer stratification and shorten the winter months during which mixing occurs, the authors wanted to understand impacts to nitrogen cycling (mostly denitrification/dissimilatory nitrate reduction to ammonium). Using a trans-disciplinary approach, the authors combined 15N-tracer assays with metagenome reconstruction and modeling to characterize seasonal dynamics and ratios between denitrification/DNRA and compared observed to modeled rates. They also centered much of this work around chitin as a potential carbon source. In general, denitrification was dominant during the mixed regime and tied strongly to POC and nitrate availability; conversely, DNRA increased in summer and late fall when denitrification decreased, likely driven by high carbon availability. Some of this denitrification, the authors speculated, could be the result of interactions between Bacteroidales and Methylonetera, who togther, can degrade chitin under oxygenated, nitrate-rich conditions. Overall, the authors stress that denitrification could decrease by 8-12% under future climate scenarios (RCP 2.6, 4.5, and 8.5) as a result of shorter mixing periods.

Overall Comments: This manuscript is well-written and combines a suite of techniques that makes for a compelling and innovative story. This narrative adds on a considerable amount of work to existing data, so please be sure to clarify what has been previously published versus what is newly presented in this manuscript (this is done well in some places already). A large portion of the story is also centered around chitin and I think the story, if space permits, would benefit from a bit more justification behind targeting that carbon source. There are some places in the methodology that could use more detail/justification – for instance, presenting nitrification potential in Fig. 1b as single cell rates/abundance versus actual measured nitrification (though perhaps I misinterpreted this) and being clear that all measurements are potential rates. Lastly, I am left wanting a bit more discussion about potential implications to water quality (e.g. if DNRA becomes more dominant over time). Overall, however, I really enjoyed this manuscript.

We appreciate the reviewer's positive feedback and are pleased that the manuscript was found to be both readable and a meaningful contribution to the field.

In response to the reviewer's suggestion, we have added further context to explain why chitin was chosen as a focus of our investigation on lines 172-180. Namely, chitin is an abundant aminopolysaccharide commonly found in sediments and has been identified in previous studies as a key substrate for microbial remineralization[1,2]. Its degradation can

release ammonium, thereby fueling nitrification and subsequent denitrification at the sediment-water interface. In our study, we observed that chitin (likely deposited during the summer phytoplankton bloom) was particularly prevalent during the mixed regime in Lake Baldegg (Fig. 2d), a period when the oxygen flux into the sediment was also at its peak. Oxygen availability is critical for chitin degradation, as chitin tends to be preserved under anoxic conditions[2–4]. Notably, the aerobic degradation of chitin monomers (e.g., N-acetylglucosamine; Eq. 1) requires 120% more molecular oxygen than the respiration of labile amino acids (Eq. 2), yet yields 207% more energy for microbial growth.

In addition, we have clarified the distinction between potential and predicted nitrification rates, as detailed in our response below, and have updated the caption of Fig. 1 accordingly (lines 705-706).

In response to the reviewer's comment regarding the use of the term "potential" rates, we now clearly state in the revised manuscript that all reported rates are considered potential rates. This clarification has been incorporated into the sections *"Nitrogen transformation rates in Baldeggersee"* and *"Influence of the mixed regime on nitrogen removal"*, as well as the Methods section under *"Quantifying sediment nitrate-reducing processes."* Moreover, based on related feedback from Reviewer 3, we have added new evidence showing that the measured potential rates are unlikely to reflect artificial stimulation and are consistent with in situ microbial activity. A summary of this supporting information is provided below:

1. Linearity of turnover rates (see lines 317-318 and Fig. S4): We highlight that the time courses used for rate calculations were strictly linear (Fig. S4), indicating that $^{15}N$ tracer turnover reflects in situ activity. In contrast, non-linear (e.g., exponential) trajectories, characterized by an initial lag phase followed by a sudden increase in $^{15}N$-labeled $N_2$ production, would suggest artificial stimulation or "bottle effects" within the microbial community.
2. Coupling of rates with active denitrifiers (see lines 97-100): We show that the measured potential rates from slurry incubations are consistent with in situ microbial activity. Specifically, depth-resolved denitrification rates ($^{29}N_2$ + $^{30}N_2$) were positively correlated with the abundance of *Methylotenera*, a known denitrifier, as quantified by CARD-FISH (which targets metabolically active cells) across depth profiles (Pearson correlation, one-tailed $p = 0.033$; $\alpha = 0.05$). This relationship suggests that our rate measurements capture real denitrification activity, rather than being artifacts of nitrate addition.
3. Whole-lake nitrogen budget comparison (see updated Fig. 1a and lines 111-112, 698-703): To further anchor our findings in natural system behavior, we now include a whole-lake nitrogen budget derived from independent in situ flux measurements (Müller et al., 2021). These fluxes are shown alongside our denitrification and DNRA rates in the revised Fig. 1a, providing additional context and ensuring that our interpretations are not solely based on slurry-derived data.

To the reviewer's last point, we also agree that an expansion of the impact of increased DNRA on water quality under a worst-case climate scenario was needed. On lines 271-274, we have added that "This anticipated increase in recycled ammonium export from lakes to downstream marine ecosystems could, in turn, promote larger coastal phytoplankton

blooms. The resulting coastal eutrophication would likely contribute to the further expansion of low-oxygen "dead zones"[5]."

Specific Comments:
L61: Purely semantics, but consider finding a synonym to "furthermore."

Thanks for pointing this out. In the revised manuscript, we have now replaced "Furthermore" with "Additionally".

L153: Can you further discuss the underestimation of POC flux to the sediments? (e.g. Fig. 2d)

We thank the reviewer for raising this discussion. We believe this underestimation stems from the fact that the model cannot resolve resuspension and lateral transport processes that sediment traps capture. This limitation can bias modeled POC fluxes low, while sediment traps may overestimate fluxes due to hydrodynamic effects and over-collection. Some mismatch in absolute values is therefore expected; nonetheless, the seasonal dynamics are well reproduced, and the model also captures the temporal patterns of other key variables. According to the reviewers' suggestion, we have amended this sentence on lines 146-149 of the revised manuscript to read as follows: "While the model tends to underestimate $R_{POC:NO_3}$ compared to observations (Fig. 2i), due to a general underestimation of modeled POC fluxes (Fig. 2c), as the model does not account for lateral inputs or sediment resuspension, it nevertheless captures the seasonal pattern of $R_{POC:NO_3}$ dynamics (Fig. 2j)."

L180: Consider providing a brief background or justification for why chitin was of interest (Text from L223 may benefit from being moved upwards in the narrative).

Indeed, the reviewer is correct in pointing out that further justification of why chitin was of interest was missing. In response, we have brought forward relevant context from later in the manuscript (originally on line 223) and included it earlier, now appearing on lines 171-180 in the revised version. The revised text reads: "Semi-labile compounds like chitin – an abundant aminopolysaccharide in sediments – are thought to play a key role in microbial remineralization, serving as important substrates for ammonium release that can fuel nitrification and subsequent denitrification at the water-sediment interface[1,2]. In our study, we observed that chitin (likely deposited during the summer phytoplankton bloom) was indeed prevalent during the mixed regime in Lake Baldegg (Fig. 2d), a period when the oxygen flux into the sediment was also at its peak. Oxygen availability is critical for microbial chitin degradation, as the compound tends to be preserved under anoxic conditions[2–4]. Notably, the aerobic breakdown of chitin monomers (N-acetylglucosamine; Eq. 1) requires 120% more molecular oxygen than the respiration of labile amino acids (Eq. 2), yet it generates 207% more energy for growth."

L201: This is certainly compelling evidence of a potential syntrophic interaction between

bacteroidales and Methylotenera, but I'd also be interested in seeing a discussion, if even brief, of some other reasons these microbial groups may co-occur. For instance, they both occur at oxic-anoxic interfaces?

We thank the reviewer for the helpful suggestion. In addition to the proposed syntrophic interaction, an alternative explanation for the observed co-occurrence of *Bacteroidales* and *Methylotenera* is that both groups may be independently favored at oxic-anoxic interfaces, where particle-bound polysaccharides accumulate and transient nitrate or oxygen conditions occur. In this scenario, methanol utilized by *Methylotenera* could be supplied independently of *Bacteroidales* (e.g., via methane oxidation), suggesting that their co-occurrence may result from parallel responses to shared environmental drivers rather than obligate coupling. Importantly, two lines of evidence argue for a potential partnership: (i) the tight seasonal (temporal) coupling of Bacteroidales and *Methylotenera* in our time series (Extended Data Fig. 5c), and (ii) the recurrence of this association across lakes spanning different trophic states. Nevertheless, we agree that offering alternative interpretations is important. The revised text on lines 204-208 now reads: "Although such co-occurrence could reflect habitat filtering at oxic-anoxic interfaces (i.e., parallel responses to shared drivers rather than obligate coupling), the two groups also exhibited a temporal coupling: over a seasonal cycle, *Methylotenera* is positively correlated with pectinolytic/chitinolytic Bacteroidales (Extended Data Fig. 5c; Pearson correlation, two-tailed $p < 0.01$; df = 12; $\alpha = 0.05$), consistent with a close metabolic partnership."

L209: Spelling of Methylotenera
Done.

L226: Where does this estimate come from?

We thank the reviewer for pointing this out; indeed, we are missing a reference to the previous sediment trap data that was collected by Müller et al., 2021. In the revised manuscript (lines 217-219), we have updated the sentence to read as, "With net organic matter export rates from the euphotic zone of Baldeggersee averaging 18.3 mmol POC $m^{-2}$ $d^{-1}$ in summer[6], the estimated chitin flux to the sediments is 1.1 mmol $m^{-2}$ $d^{-1}$."

L315: I'd be curious if any of these measurements were performed in triplicate? I appreciate that all data are visualized in the figures, but an indication of n/df will be helpful for context.

We thank the reviewer for this question. Rate incubations were not performed in technical triplicates. However, for each core we analyzed multiple discrete depth intervals from the upper sediments (n = 10 independent sections). These depth-specific rates were normally distributed between 0-5 cm (Shapiro–Wilk $p$ = 0.993) and exhibited moderate variability, with a coefficient of variation (CV) of 16.2% within a given core. This consistency supports treating the discrete vertical horizons as biological replicates for statistical purposes. For subsequent analyses (e.g., Fig. 2k, m), these depth-resolved measurements were grouped according to the prevailing mixing regime, either mixed or stratified, resulting in 18 rate

measurements for the mixed regime (*n* = 18) and 40 for the stratified regime (*n* = 40). We believe that this clarification is best placed in the Figure 2 caption (lines 724-729), where it provides immediate context for the displayed data, rather than in the Methods section. As noted by the reviewer, all individual data points are displayed in the corresponding figure (Figure 2).

L519: All deposited data, intermediate files, and MAGs were located online as described in the manuscript.

Thank you for confirming. We appreciate the reviewer's attention to data availability. As noted in the manuscript, all deposited rate measurement data and MAGs are available online as described. In addition, we have included more information regarding the model accessibility, which reads as follows (Lines 501-505): "The General Lake Model (GLM; version 3.1) coupled with the Aquatic EcoDynamics module (AED; version 2) was used in this study. GLM is a freely available, open-access modeling framework developed by the University of Western Australia and can be downloaded at https://aed.see.uwa.edu.au/research/models/glm/latest_release.html. Parameters used to calibrate the General Lake Model (GLM) used in this study are provided in Tables S5-S7."

Fig. 1: Is there a reason the measured potential nitrification rates from 15NH4+ were not presented here? The figure itself states "Nitrification (Predicted)," but please also make this correction in the figure caption – "Predicted sediment nitrification activity" (L787). If these are the data presented here, can this also be described in the supplemental methods? Apologies if missed.

For clarification, we only measured potential nitrification rates from $^{15}NH_4^+$ incubations in the water column (this was never done for the sediment). And the method is indeed described in the supplementary material under the section heading, "Quantifying water column ammonium turnover by nitrification-assimilation". In the sediment, nitrification activity was extrapolated based on the enumerated cell abundances of Thaumarchaeota (using CARD-FISH), combined with the single-cell rates from Klotz et al., (24), which is what we refer to as "Nitrification predicted". In the figure caption (lines 705-707), we have now added "Predicted sediment nitrification activity" according to the reviewers' suggestion. We thank the reviewer for highlighting this and hope the clarification is helpful.

Fig. 3b: Could benefit from slightly more detailed description about how the MAGs presented in Fig. 3b are visualized between the mixed and stratified. I think between the different conditions, presence/absence, I am losing the point of the figure just a bit.
Thank the reviewer for highlighting this confusion, and realize that Fig. 3b needed some clarification. Panel 3b compares the species-resolved MAG assemblages recovered from mixed (M) versus stratified (S) sediments and their encoded N/S-cycling pathways. The left column lists MAGs assembled in the mixed regime; the right column lists those from the stratified regime. Rows are ordered by a phylogenomic tree, and when the same species

occurred in both regimes (ANI ≥ 95%), the corresponding bins are shown on the same row (paired across M and S). Open red circles mark MAGs detected/passing QC in that regime; "b.d." indicates the genome was below detection or did not meet QC in the other regime. Presence/absence bars (yellow = present, purple = absent) summarize key pathway modules for the representative MAG of that species (shown once to avoid duplication). In total, we recovered 17 MAGs in the mixed regime and 12 in the stratified regime; 11 species were shared across regimes, while 6 were only detected in the mixed regime – illustrating a shift in community composition under the mixed regime. We have since updated the figure caption to include the information above, which can now be found on lines 754-763 in the revised manuscript. In addition, we realize that part of the confusion might have stemmed from the different numbering system we had between bins collected in the mixed and stratified regimes; we have now unified this number system in the revised Fig. 3b and in Table S2.

Reviewer #3 (Remarks to the Author):

Dear Authors,

I read your article with great interest. I believe the topic is important given the current trend of many lakes toward increasingly prolonged stratification periods, which lead to the separation of surface and deep water masses. In this context, it is crucial to ask what the biogeochemical implications of such physical processes might be. In this article, the combination of experimental and modeling studies indicates that the lake in question will lose denitrification capacity in favor of increased ammonium nitrogen recycling. This is due to several factors, the most important of which appears to be the seasonal denitrification rate, which peaks during periods of full mixing—periods that are expected to become shorter. This result seems realistic and likely.

In general, the article is modern and integrates strictly experimental aspects—based both on stable isotope and molecular analyses—with a well-established model that represents physical, chemical, and biological processes in the system. The results and discussion section is well-written and generally supported by data.

The less convincing parts, in my opinion, that require further effort from the authors, concern the introduction and the materials and methods sections. While the introduction is clear and well-written, it lacks a "technical" section that describes the working hypotheses based on a solid understanding of the nitrogen biogeochemical cycle. I do not see an initial reasoning that links an expected increase in stratification period (or a reduction in full mixing duration) with the coupled dynamics of oxygen, organic matter, and dissimilatory nitrate reductions. I believe the authors are capable of including such a section, which would help formulate working hypotheses that are currently missing.

We appreciate the reviewer's positive feedback, and we're especially grateful for the reviewer's appreciation of the multidisciplinary approach. We thank the reviewer for raising this point, and agree that the working hypothesis was rather vague in the original version of the manuscript. In the revised manuscript, we have now articulated a more explicit hypothesis on lines 44-49, outlining how prolonged stratification, coupled with a shortening of the seasonal mixing period, could shift the balance between denitrification and DNRA. One possibility is that extended stratification may expand the niche for phytoplankton growth, leading to increased organic matter flux to the sediments and more persistent anoxia, conditions known to favor DNRA over denitrification. This is consistent with studies showing that DNRA is promoted under high organic carbon to nitrate ratios[7] and more strongly reducing conditions[8]. In contrast, the seasonal mixing period re-oxygenates the water column and likely represents a critical window for fixed nitrogen removal via coupled nitrification–denitrification. If this window continues to shrink with climate-driven warming, the lake's overall denitrification capacity could decline.

The introduction is solid, and the citations are appropriate, but both for a reader unfamiliar with the nitrogen cycle and for an expert, there is a lack of minimal guidance on the biogeochemical drivers of denitrification and DNRA, and on the relative importance of allochthonous nitrate and nitrate produced within the lake (through nitrification in water and sediments) in regulating these two processes. These aspects are well known and would allow for hypothesis generation. Without them, it appears the authors simply performed measurements and then interpreted the results afterward.

We thank the reviewer for noting this omission. We agree it was not stated explicitly in the introduction, so we have moved this information forward. The revised introduction now highlights the $R_{POC:NO3}$ ratio as a key control on the balance between denitrification and DNRA. It reads (lines 32-35): "The pathways for recycling and/or removing fixed nitrogen co-occur in varying proportions in sediments[9–12], with evidence suggesting that DNRA becomes more important when organic carbon is high relative to nitrate[7], and under more reducing conditions[8]." We also make this mechanism explicit (see also our response above and on lines 44-49): prolonged stratification expands phytoplankton niches, increases organic-matter flux to sediments, and deepens anoxia – conditions that favor DNRA over denitrification. Conversely, the seasonal mixed regime re-oxygenates the water column and provides a key window for nitrification–denitrification; if this window shrinks with warming, the lake's overall denitrification capacity could decline.

In the materials and methods section, and wherever else in the article it is relevant, it seems important to stress that the authors measured potential rather than actual activities, using a method that is not entirely clear to me. For a study of this significance, I am surprised the authors did not incubate intact sediment cores using the isotope pairing technique (IPT). It appears they added labeled nitrogen in exetainers, for a series of potential rate measurements at different sediment horizons. Moreover, the exetainers

were incubated in a horizontal shaker, whereas the standard approach is to rotate them to ensure continuous sediment resuspension. It is important to understand why intact core incubations were not carried out, especially considering the study's objectives, for the reasons summarized below, which I would like the authors to address:

We appreciate the reviewer's comments on intact-core IPT versus slurry incubations. Intact cores have a strong track record, but in Lake Baldegg, the method is made more difficult by intense bio-irrigation (≈8,500 *Tubifex* worms $m^{-2}$ [13]). A visual inspection of the sediment reveals that these worms form intricate burrow networks extending to depths of up to 10 cm (e.g., Fig. 1c). These worms (3-5 cm in length) ventilate their burrows by drawing bottom water, rich in $O_2/NO_3^-$, downward toward their head embedded in anoxic sediment, thereby delivering oxidants to depth. Our own high-resolution profiles show episodic nitrate anomalies at depth and an absence of free sulfide within the *Tubifex*-burrowed layer, both consistent with oxidant transport by animals that span oxic-anoxic zones (e.g., Extended Data Fig. 1, see also the revised supplementary section "Sediment oxygen dynamics and bioirrigation"). In this bioirrigated setting, several key assumptions required for applying the IPT intact-core method, described by Robertson et al.[14], are difficult to satisfy:

1. Non-uniform $^{15}NO_3^-$ labeling. Burrow ventilation creates oxic/anoxic micro-niches and subsurface nitrification along burrow walls (e.g., Extended Data Fig. 1), which could yield patchy $^{14}NO_3^-$:$^{15}NO_3^-$ in the nitrate-reducing zone.
2. Unknown, dynamic oxidant penetration. Faunal pumping makes $O_2/NO_3^-$ penetration depth time-variable, so a reliable $^{15}NO_3^-$ pre-equilibration is difficult to define; time-series can confirm linear $N_2$ production but cannot resolve the underlying transport path length. Consistent with this, dense *Tubifex* irrigation delivers $O_2/NO_3^-$ below the nominal penetration horizon, seen as nitrate anomalies at depth and the absence of free sulfide in the burrowed layer (e.g., Extended Data Fig. 1), allowing denitrifiers like Methylotenera to persist beneath the shallow interface despite negligible sediment nitrification (Fig. 1c).
3. High between-core variance. Irrigation rates differ among cores, introducing core-to-core differences in labeling and bias/noise in IPT partitions (DW vs DN).

This is not to say intact cores are unusable, but in Baldegg they would require a dedicated, fauna-focused program (e.g., sieved/reconstructed cores, controlled worm densities, long pre-incubations, constraints on sediment reworking and organic-matter composition), which is beyond the scope here and introduces additional artefacts.

Given these constraints, we used slurry incubations (in exetainers like other studies[15,16]) to resolve potential N transformation activity across the upper sediments without labeling artefacts from heterogeneous burrow networks. Sediments were homogenized with $^{15}N$ tracer in sterile bottom water and placed on a horizontal shaker. Unlike the traditional end-over-end rotation, horizontal agitation allows particles to settle while gentle motion sustains solute exchange ($O_2/NO_3^-$) across grains, avoiding continuous high-shear resuspension that can disrupt microbial aggregates[17]. This choice and its rationale are now stated in the revised text (lines 304-307).

In response to the reviewer's comment regarding the use of the term "potential" rates, we now clearly state in the revised manuscript that all reported rates are considered potential rates. This clarification has been incorporated into the sections *"Nitrogen transformation rates in Baldeggersee"* and *"Influence of the mixed regime on nitrogen removal"*, as well as the Methods section under *"Quantifying sediment nitrate-reducing processes."* Moreover, we have added new evidence showing that the measured potential rates are unlikely to reflect artificial stimulation and are consistent with in situ microbial activity. A summary of this supporting information is provided in our response below.

Slurries allow for the measurement of potential activities, which often differ significantly from actual rates. Microbial communities are placed in optimal conditions to carry out various processes. In the specific case of denitrification, many sediment bacteria have multiple possible metabolic pathways, not all of which are expressed depending on the availability of an electron acceptor such as nitrate. I believe the potential denitrification rates reported by the authors might be considerably overestimated. The same applies to potential DNRA rates. Why weren't nitrification rates measured in the same way, to obtain a complete and comparable set of bacteria-mediated processes? If these measurements are potential, what is the rationale for using them in the authors' calculations? The nitrogen cycle cannot be built on volumetric potential rate measurements. Denitrification and DNRA should be limited by the nitrate penetration into sediments, especially if—as suggested by the figure—nitrification is negligible. If the main source of nitrate for both processes is the water column, then the Den:DNRA potential ratio in the nitrification zone is 10:1.

We thank the reviewer for raising this discussion and realize that we need to clarify some points regarding the $^{15}N$-$NO_3^-$ slurry incubation rate measurements. Addressing first the reviewers' criticism, on whether slurries can be trusted to represent in situ activity, and if rates are overestimated.

**Linearity of potential turnover rates.** In our time-series slurries, $^{29}N_2$ and $^{30}N_2$ production increased linearly with time, which is consistent with ongoing in situ activity rather than artificially stimulated rates (Fig. S4). In contrast, non-linear (e.g., exponential) trajectories, characterized by an initial lag phase followed by a sudden increase in $^{15}N$-labeled $N_2$ production, would suggest artificial stimulation or "bottle effects" within the microbial community. The linearity of turnover rates is now detailed on lines 317-318 and in the newly added Fig. S4.

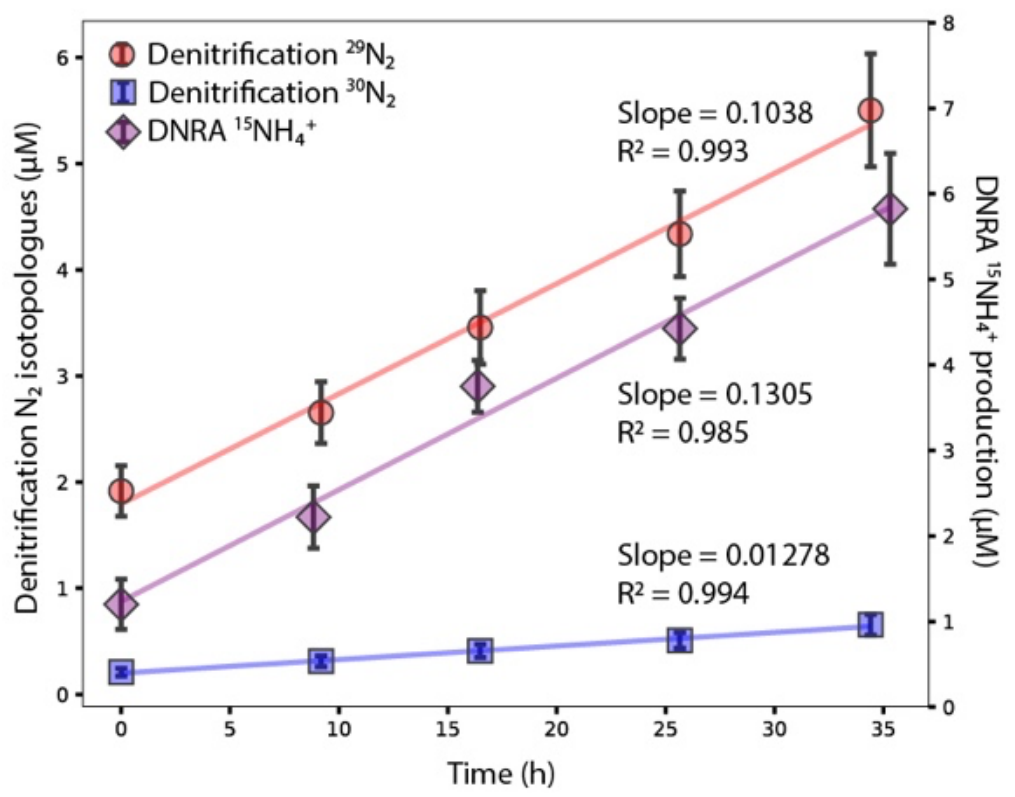


**Figure S4 (NEW figure): Linearity of potential denitrification and DNRA rates over time.** Seasonally aggregated samples are shown for denitrification (n = 58) and DNRA (n = 32). Linear regression lines are plotted for each respective isotopologue, with corresponding slope and $R^2$ values indicated.

**Coupling of potential rates with active denitrifiers.** Vertically, rates were high in the

upper few centimeters of the sediment and declined below ~5 cm (Fig. 1b); the very low activity at 10-15 cm shows that adding nitrate did not uniformly stimulate production. More convincingly, rate measurement activity is tightly coupled to the presence of active denitrifiers, like *Methylotenera* (Fig. 1c). Consistent with our directional hypothesis, depth-resolved potential denitrification rates were positively associated with the vertical distribution of *Methylotenera* abundances (as quantified by CARD-FISH, which detects active cells; Pearson, one-tailed p = 0.033; α = 0.05). Taken together, the linear time courses, vertical attenuation, and rate-denitrifier coupling indicate that the measured rates track in situ denitrifier activity rather than reflecting uniform stimulation by nitrate amendments. In the revised manuscript, we have now highlighted the coupling of rates with active denitrifiers on lines 97-100.

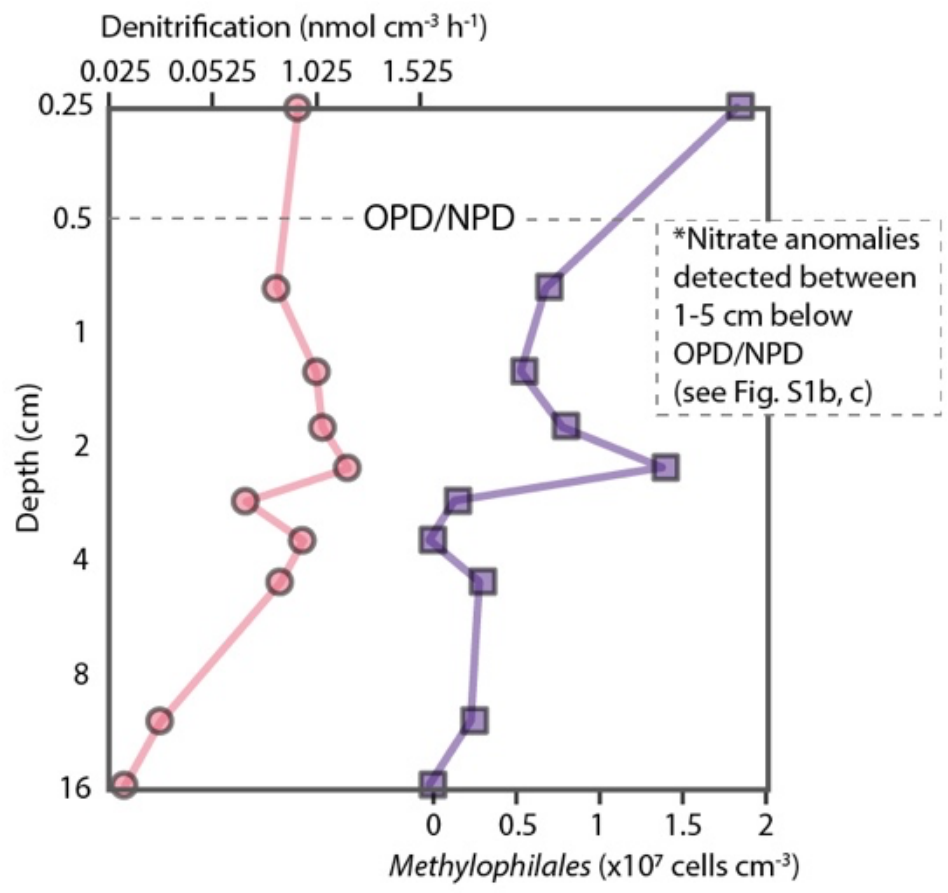


**Figure 1b, c (NEW analysis): Vertical distribution of potential denitrification rates and Methylophilales.** The order Methylophilales comprises the denitrifying genus *Methylotenera, e.g., Bin13M in Fig. 3c). Note:* Methylophilales abundance was determined using CARD-FISH, which targets ribosomal 16S rRNA molecules within intact cells. Because rRNA content correlates with metabolic activity, CARD-FISH preferentially detects active microbial populations, rather than dormant or inactive cells. Oxygen- and nitrate-penetration depths (OPD and NPD) are denoted according to microsensor/microrhizon concentration profiles shown in Extended Data Fig. 1.

**Whole-lake nitrogen budget comparison and addressing the concern that our potential denitrification rates may be overestimated.** Mean volumetric rates derived from seasonal $^{15}N$ slurry incubations were 0.50 and 0.77 nmol $cm^{-3}$ $h^{-1}$ for DNRA and denitrification, respectively – corresponding to a total nitrate reduction rate of 1.3 nmol $cm^{-3}$ $h^{-1}$. Depth integration over the upper 5 cm of sediment (where high-resolution profiles are available) yields 0.6 mmol $m^{-2}$ $d^{-1}$ for DNRA and 0.9 mmol $m^{-2}$ $d^{-1}$ for denitrification, or 1.5 mmol $m^{-2}$ $d^{-1}$ in total. This value agrees well with the measured downward nitrate flux of 1.9 ± 0.5 mmol $m^{-2}$ $d^{-1}$, indicating that slurry-derived rates are not inflated for Lake Baldegg. To further assess this, we revisited the whole-lake nitrogen budget reported by Müller et al. (2021) to derive an independent constraint on nitrate reduction processes in Baldeggersee (Fig. 1a, NEW). According to their data, gross nitrogen sedimentation is 3.3 ± 1.2 mmol N $m^{-2}$ $d^{-1}$, with 1.0 mmol N $m^{-2}$ $d^{-1}$ buried, leaving 2.3 mmol N $m^{-2}$ $d^{-1}$ remineralized to ammonium ($NH_4^+$). Given that the observed upward $NH_4^+$ flux is 3.1 ± 0.5 mmol N $m^{-2}$ $d^{-1}$, the difference (0.8 mmol N $m^{-2}$ $d^{-1}$) can reasonably be attributed to DNRA. The remaining portion of the nitrate flux, approximately 1.1 mmol N $m^{-2}$ $d^{-1}$, is thus likely consumed by denitrification. This independent budget-based estimate supports the reliability of our measured rates and suggests that they are conservative. While slurry incubations inherently reflect potential rates rather than exact in situ fluxes, our values align closely with whole-

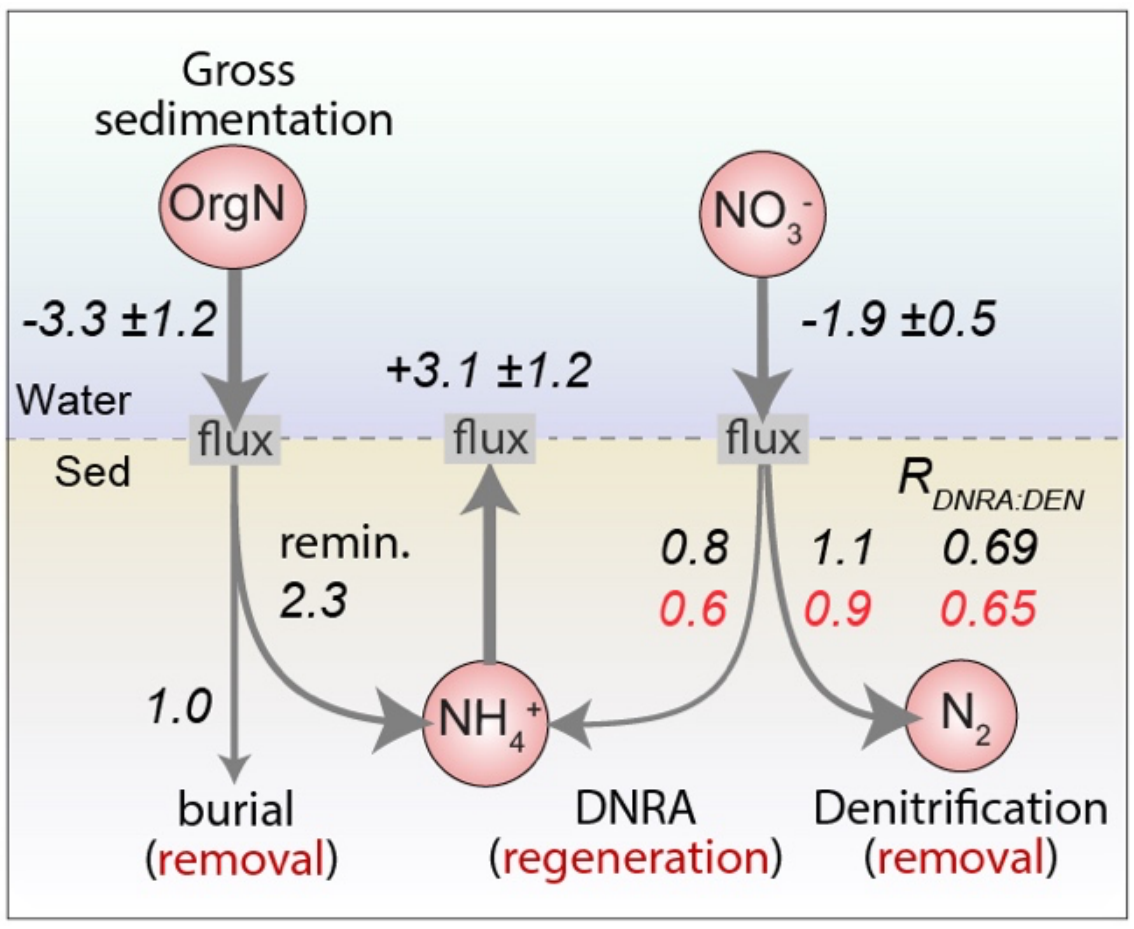


**Figure 1a (NEW figure): Potential rates vs. whole-lake nitrogen budget.** Shown is a graphical depiction of the benthic fluxes (from Müller et al., 2021) and the process rates related to denitrification and DNRA in mmol $m^{-2}$ $d^{-1}$. The DNRA:denitrification ratio ($R_{DNRA:Den}$) is shown as predicted (black) and measured (red). Predicted values were calculated from the benthic fluxes by Muller et al., (2021), whereas the measured values derive from $^{15}N$ tracer incubations.

system constraints. Most importantly, the partitioning between DNRA and denitrification – the central focus of this study – is well represented. Our measured rates (Den = 0.77; DNRA = 0.50 nmol $cm^{-3}$ $h^{-1}$) yield an $R_{DNRA:Den}$ ratio of approximately 0.65, closely matching the budget-derived ratio of 0.69. This implies a Den:DNRA ratio of ~1.4–1.6, not 10:1, as suggested by the reviewer.

To make this clear in the manuscript, we now include a whole-system nitrogen budget derived from in situ flux measurements (Müller et al., 2021) alongside our slurry-based rates, ensuring that interpretations are constrained by independent system-scale fluxes rather than slurry data alone (see revised Fig. 1a and lines 111-112, 698-703). With the exception of Fig. 1a, which is used to contextualize our $^{15}N$ slurry measurements within a broader areal nitrogen budget, we now report $^{15}N$ rate measurements in volumetric terms (nmol $cm^{-3}$ $h^{-1}$) rather than as areal rates (mmol $m^{-2}$ $d^{-1}$) throughout the text (e.g., lines 117-120 and in Fig. 2k, m, l, n). This change avoids the impression that we derived areal budgets and more accurately reflects the data as volumetric turnover rates, without extrapolation to sediment surface area. It is important to note that expressing rates volumetrically rather than as depth-integrated values does not affect the overall trends, which focus on seasonal fluctuations.

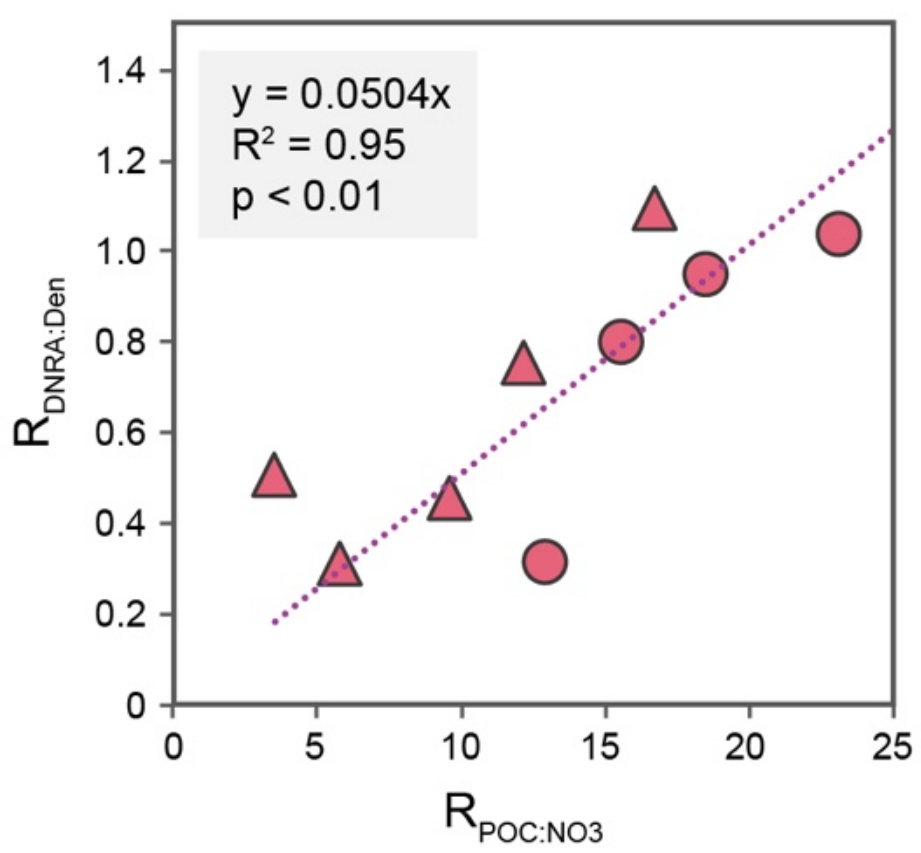


**Extended Data Fig. 2c: Relationship between $R_{DNRA:Den}$ and $R_{POC:NO3}$.** Correlation plot showing the observed relationship between $R_{DNRA:Den}$ and $R_{POC:NO3}$ for Lake Baldegg (triangles) and Lake Sarnen (circles). A linear regression and t-test were performed to assess the statistical significance.

**Lastly, the partitioning between the potential rates of DNRA and denitrification varies according to known environmental drivers.** Namely, a collation of data from Baldeggersee, and an oligotrophic lake from the same region reveals a robust correlative seasonal trend ($\omega^{obs}$; Eq. 9) between the ratios of $R_{POC:NO3}$ and $R_{DNRA:Den}$ (Extended Data Fig. 2c; t-test, two-tailed p = 0.01; df = 7; α = 0.05). This reinforces the role that organic matter and nitrate availability play in determining whether fixed nitrogen is denitrified to dinitrogen gas or regenerated back to ammonium (via DNRA), in line with previous work[18,19]. These results highlight that the rates measured using the $^{15}N$ slurry method covary according to meaningful environmental drivers, rather than reflecting artificial stimulation effects.

On the topic of nitrification, the reviewer asks, "Why weren't nitrification rates measured in the same way, to obtain a complete and comparable set of bacteria-mediated processes?" We did not perform sediment nitrification tracer assays because (i) prior surveys detected few nitrification genes and (ii) existing FISH data indicated predicted rates would range between 0-0.004 nmol $cm^{-3}$ $h^{-1}$, which would be at the limit of detection (0.002 nmol $cm^{-3}$ $h^{-1}$). Instead, we quantified nitrification-related activity in the water column, where bottom waters in contact with ammonium-rich sediments are most active.

**Addressing the source of nitrate supporting nitrate-reducing processes in sediments.** Given the presence of the denitrifier *Methylotenera* and active nitrate reduction occurring well below the nitracline, this raises the question of what supplies nitrate to sustain denitrifiers? Local sediment nitrification is negligible, with predicted rates more than two

orders of magnitude lower than measured nitrate reduction rates (Fig. 1b). Therefore, the primary source of nitrate fueling sediment DNRA and denitrification is the water column, where nitrate enters sediments via passive diffusion and is further transported to depth through bioirrigation by *Tubifex* worms. Tubifex worms (3-5 cm in length) are abundant in situ and ventilate their burrows by drawing bottom water, rich in $O_2/NO_3^-$, downward toward their head embedded in anoxic sediment, thereby delivering oxidants to depth. High-resolution profiles show episodic nitrate anomalies at depth and an absence of free sulfide within the *Tubifex*-burrowed layer, both consistent with oxidant transport by macrofauna that span oxic-anoxic zones (e.g., Extended Data Fig. 1). We suspect that although *Tubifex* likely supplies nitrate continuously to depth, rapid nitrate reduction (with mean rates of 0.9 nmol $cm^{-3}$ $h^{-1}$) keeps ambient concentrations low; only transient anomalies are detectable. In the revised manuscript, Fig. 1a and lines 100-105, now make explicit that water-column nitrate is the primary source fueling denitrification and DNRA.

Incubating intact cores using IPT would have allowed the authors to analyze the benthic nitrogen cycle much more accurately and with less speculation. Bioturbation processes have been extensively studied and sometimes reveal surprisingly different effects on nitrogen dynamics. For example, in Baltic Sea sediments, Marenzelleria tends to stimulate DNRA more than denitrification. With the data currently available, it is not easy to assess the role of bioturbation in the studied sediments in relation to nitrification, denitrification, and DNRA rates. With the isotope techniques used by the authors to measure potential rates, this aspect could have been easily revealed. Even more importantly, the relative contributions of nitrate reduction processes supported by nitrate from the water column versus that from nitrification in the water and sediments are unclear. With IPT, at least in sediments, processes such as Dn and Dw, rather than DNRAn and DNRAW, can be readily measured.

We appreciate the reviewer's interest in bioturbation and its influence on the nitrogen cycle. We agree that this is a compelling and understudied topic, particularly in the context of how Tubifex worms may affect rate process measurements. Baldeggersee hosts a high density of ~8,500 Tubifex $m^{-2}$ [13], making it a strongly bioirrigated sediment system. A visual inspection of the sediment reveals that these worms form intricate burrow networks extending to depths of up to 10 cm (e.g., Fig. 1c). While we acknowledge that intact core-based IPT approaches could theoretically be applied in such sediments, implementing them effectively in Baldegg would require a dedicated, fauna-focused experimental design, including measures such as sieved or reconstructed cores, controlled worm densities, extended pre-incubation times, and careful control of sediment reworking and organic matter composition. These additional requirements, along with the potential for introducing artefacts, were beyond the scope of the current study. Our primary goal was not to assess the role of bioturbation per se, but rather to provide context for the presence of macrofauna and their potential influence on the vertical distribution of rates and denitrifier populations (e.g., *Methylotenera*). Where relevant, we cite studies that directly examine macrofaunal effects on specific nitrogen transformation processes.

Given the highly bioirrigated nature of the sediments, and our goal of understanding the general partitioning between DNRA and denitrification across a seasonal cycle, we chose to use $^{15}N$ slurry incubations (in exetainers like other studies[15,16]). This helps to resolve potential N transformation activity across the upper sediments without labeling artefacts from heterogeneous burrow networks (discussed in our earlier response). As outlined above, we minimized "potential rate" concerns by (i) retaining only linear $^{29}N_2/^{30}N_2$ time courses, (ii) showing depth-dependent declines consistent with denitrifier abundance, and (iii) validating magnitudes against independent whole-lake N-flux constraints. Together, these checks indicate that the observed signals reflect in situ activity rather than slurry-induced stimulation. This was altogether aimed at understanding the seasonal partitioning between denitrification and DNRA, the key microbial players involved, and their sensitivity to warming under a worst-case climate scenario (RCP 8.5).

Regarding the source of nitrate supporting nitrate-reducing processes, we have further clarified this in our response above under the paragraph heading "Addressing the source of nitrate supporting nitrate-reducing processes in sediments."

Regarding the above considerations, I believe it is not marginal for the authors to address the well-known effects of anoxia or hypoxia on the nitrogen cycle. In the specific case of this lake, if denitrification is mainly supported by high nitrate concentrations in the water column (as it seems), a progressive increase in stratification could lead to a decline in bioturbator biomass and reduced oxygen penetration into sediments. In this case, nitrate diffusion fluxes into the denitrification zone could become much faster, potentially causing denitrification rates to increase significantly. If, on the other hand, sediment nitrification—perhaps facilitated by bioturbators—mainly supports denitrification (though this is not evident or clear from the text), increased hypoxia or anoxia could completely inhibit this process and favor ammonium recycling, regardless of the Den:DNRA ratio. These aspects, in my opinion, are important, as they could lead to very different conclusions.

We thank the reviewer for raising this important point regarding the role of low-oxygen conditions and their impact on the balance between denitrification and DNRA. The reviewer is correct in noting that the manuscript devotes limited attention to anoxia during the summer stratified period and its influence on these processes in Lake Baldegg.

This was a deliberate framing choice: the study focuses on the mixed regime, which is when the lake removes a disproportionately large fraction of its annual fixed nitrogen load (49-64% more fixed nitrogen is removed per day compared to the stratified regime). While we identified $R_{POC:NO_3^-}$ as a key driver of the $R_{DNRA:Den}$ ratio, we also included a section (see lines 160-182) discussing the role of high oxygen concentrations, specifically during re-ventilation in the mixed regime, and their stimulatory effect on denitrification.

Although not explicitly detailed in the main text, the reciprocal scenario to that mentioned above is that DNRA appears to be favored under the more organic matter-rich and

hypoxic/anoxic conditions of the summer stratified period (see Fig. 2I, f), which would align with the second scenario proposed by the reviewer, where denitrification may be suppressed. Regarding macrofauna, we unfortunately do not have robust seasonal biomass data. However, Tubifex worms, which dominate the benthos in Baldegg, are highly tolerant of low oxygen and remain active during stratification. As such, they likely continue to transport water column nitrate to DNRA bacteria in the sediments, even in the absence of sediment nitrification.

Due to the word constraints and the scope of this study, we chose not to explore in detail the potential enhancement of DNRA during the stratified summer period. Instead, we framed the manuscript around the re-ventilated mixed regime, which we argue represents the most biogeochemically relevant window for fixed nitrogen removal in this monomictic lake system.

From how the methods section is written, I gather that if potential rates were measured by adding oxygen to the exetainers, the authors likely measured a mix of nitrification (which produces only $14NO3^-$), denitrification, and DNRA—thus altering the $14/15NO3^-$ ratios during incubation. This complicates calculations and the interpretation of the results. Typically, potential rates are calculated with pre-incubations long enough to deplete oxygen and $14NO3^-$, followed by the addition of $15NO3^-$. I ask the authors for clarification on this point.

We appreciate the reviewer's question about whether nitrification in the incubation experiments could confound our denitrification and DNRA rate calculations. To clarify, the bottom water was pre-filtered to remove pelagic microbes, including nitrifiers, before it was used to fill off sediment containing exetainers. In addition, as shown in Fig. 1b, sediment nitrification is absent or below detection. Specifically, based on our enumerated Thaumarchaeota cell abundances and known single-cell activity rates for nitrifiers[20], we estimate nitrification rates between 0 and 0.004 nmol $cm^{-3}$ $h^{-1}$ (Fig. 1b). For context, these rates are two orders of magnitude lower than our measured denitrification rates. Additionally, nitrifiers have relatively slow doubling times (> 24 hours per doubling, e.g., *N. maritimus*[21]), making it highly unlikely that they could establish appreciable biomass during the ~36-hour incubations, even in the presence of oxygen. We acknowledge that this approach is well-suited to the eutrophic sediments of Lake Baldegg; in more oligotrophic systems with strong nitrification-denitrification coupling, a different design might be required. Nevertheless, in Baldeggersee, we are confident that the labeled $^{14/15}NO_3^-$ pool was not significantly altered by nitrification during our incubations. We have now updated the revised manuscript with this information above (please see lines 334-342).

Indeed, while it's typical to run these experiments in the absence of oxygen, we chose to add low micromolar levels of oxygen to mimic the potential oxygen injection by bioirrigation into deeper sediment layers. As we describe in the Supplementary Discussion, Lake Baldegg's sediments are densely populated by Tubifex worms, with an estimated 8,500 individuals per $m^2$ [13]. These worms create burrow networks extending 0-10 cm into the sediment (Extended Data Fig. 1d). Tubifex are about 4-5 cm long and feed head-down, with their tails in the oxic water and heads 3-5 cm deep in anoxic sediment. By moving their tails rhythmically, they actively transport oxygen (via hemoglobin) from the water into deeper, oxygen-poor layers.

This oxygen transport is supported by our sediment amendment experiments, where adding more Tubifex led to measurable oxygen accumulation between 1-6 cm depth (Extended Data Fig. 1a). In the same zone (2-5 cm), we observed nitrate and sulfate anomalies (Extended Data Fig. 1b, c), indicating active solute transport. Sulfide levels were also lowest between 1-6 cm (Extended Data Fig. 1c), likely due to oxidation by oxygen or nitrate, further confirming this as a bioirrigated zone. Together, these Baldeggersee profiles suggest that even in otherwise anoxic sediments, nitrate (and oxygen) are intermittently present, allowing for both aerobic and anaerobic nitrogen transformations, a pattern that has also been observed in other sediment systems[22].

I would suggest clearly stating how microbial nitrogen transformation rates (including the seasonal ones mentioned) were measured and used, and to discuss any limitations of these measurements. I also ask the authors to clarify what primarily supports denitrification and DNRA (nitrate from the water column or that produced via nitrification?).

We thank the reviewer for the helpful suggestion and agree that the experimental design and limitations could be more clearly presented. In the revised manuscript, we have clarified several key aspects (as mentioned above): the use of oxygen in the incubations and the rationale for horizontal shaking (see lines 304-307 and 334-335, and also the Supplementary Discussion), the potential for nitrification to confound rate calculations (lines 334-342), and additional justification for the rate measurements (lines 289-291). Lastly, we have further clarified how $^{15}NH_4^+$ production related to DNRA was determined on lines 312-315.

In response to the reviewer's comment on limitations, we now consistently refer to the rates as "potential activity", representing upper-bound estimates of microbial processes. This clarification has been incorporated throughout the manuscript, particularly in the sections titled *"Nitrogen transformation rates in Baldeggersee"*, *"Influence of the mixed regime on nitrogen removal"*, and the Methods section under *"Quantifying sediment nitrate-reducing processes."* To further support the validity of these potential rate measurements, we: (i) ensured strict linearity in tracer time courses (lines 317-318; Fig. S4), indicating that rates reflect in situ activity; (ii) demonstrated a depth-resolved correlation between denitrification rates and active denitrifiers (*Methylotenera*, quantified by CARD-FISH; $p = 0.033$; lines 97-100); and (iii) showed consistency with a whole-lake nitrogen budget based on independent in situ fluxes (Fig. 1a; lines 111-112, 698-703), confirming that our rates are within realistic system-level constraints.

Regarding the reviewer's second point, in the revised manuscript, Figure 1a (and lines 100-105) now makes explicit that water-column nitrate is the primary source fueling denitrification and DNRA.

**References cited in the response to reviewer comments:**


1. Kollner, K. E. *et al.* Bacterial chitin hydrolysis in two lakes with contrasting trophic statuses. *Appl Environ Microbiol* **78**, 695–704 (2012).
2. Gajendra, N. *et al.* Carbohydrate compositional trends throughout Holocene sediments of an alpine lake (Lake Cadagno). *Front Earth Sci (Lausanne)* **11**, 1–17 (2023).
3. Stankiewicz, B. A., Briggs, D. E. G., Evershed, R. P., Flannery, M. B. & Wuttke, M. Preservation of Chitin in 25-Million-Year-Old Fossils. *Science (1979)* **276**, 1541–1543 (1997).
4. Cody, G. D. *et al.* Molecular signature of chitin-protein complex in Paleozoic arthropods. *Geology* **39**, 255–258 (2011).
5. Diaz, R. J. & Rosenberg, R. Spreading dead zones and consequences for marine ecosystems. *Science (1979)* **321**, 926–929 (2008).
6. Müller, B., Thoma, R., Baumann, K. B. L., Callbeck, C. M. & Schubert, C. J. Nitrogen removal processes in lakes of different trophic states from on-site measurements and historic data. *Aquat Sci* **83**, (2021).
7. Kraft, B. *et al.* The environmental controls that govern the end product of bacterial nitrate respiration. *Science (1979)* **345**, 676–679 (2014).
8. Behrendt, A., de Beer, D. & Stief, P. Vertical activity distribution of dissimilatory nitrate reduction in coastal marine sediments. *Biogeosciences* **10**, 7509–7523 (2013).
9. Mengis, M., Gächter, R., Wehrli, B. & Bernasconi, S. Nitrogen elimination in two deep eutrophic lakes. *Limnol Oceanogr* **42**, 1530–1543 (1997).
10. Robertson, E. K., Roberts, K. L., Burdorf, L. D. W., Cook, P. & Thamdrup, B. Dissimilatory nitrate reduction to ammonium coupled to Fe(II) oxidation in sediments of a periodically hypoxic estuary. *Limnol Oceanogr* **61**, 365–381 (2016).
11. Song, G. D., Liu, S. M., Marchant, H., Kuypers, M. M. M. & Lavik, G. Anammox, denitrification and dissimilatory nitrate reduction to ammonium in the East China Sea sediment. *Biogeosciences* **10**, 6851–6864 (2013).
12. Palacin-Lizarbe, C. *et al.* The DNRA-Denitrification Dichotomy Differentiates Nitrogen Transformation Pathways in Mountain Lake Benthic Habitats. *Frontiers in Microbiology* vol. 10 1229 Preprint at https://www.frontiersin.org/article/10.3389/fmicb.2019.01229 (2019).
13. Fiskal, A. *et al.* Carbon sources of benthic fauna in temperate lakes across multiple trophic states. *Biogeosciences* **18**, 4369–4388–4369–4388 (2021).
14. Robertson, E. K. *et al.* Application of the isotope pairing technique in sediments: Use, challenges, and new directions. *Limnology and Oceanography: Methods* vol. 17 112–136 Preprint at https://doi.org/10.1002/lom3.10303 (2019).
15. Bourceau, O. M. *et al.* Simultaneous sulfate and nitrate reduction in coastal sediments. *ISME Communications* **3**, (2023).
16. Jensen, M. M., Thamdrup, B. & Dalsgaard, T. Effects of specific inhibitors on anammox and denitrification in marine sediments. *Appl Environ Microbiol* **73**, (2007).
17. Arrojo, B., Mosquera-Corral, A., Campos, J. L. & Méndez, R. Effects of mechanical stress on Anammox granules in a sequencing batch reactor (SBR). *J Biotechnol* **123**, (2006).
18. Kraft, B. *et al.* Nitrogen cycling. The environmental controls that govern the end product of bacterial nitrate respiration. *Science (1979)* **345**, 676–679 (2014).
19. Baumann, K. B. L. *et al.* Metagenomic and -transcriptomic analyses of microbial nitrogen transformation potential, and gene expression in Swiss lake sediments. *ISME Communications* **4**, ycae110 (2024).

20. Klotz, F. *et al.* Quantification of archaea-driven freshwater nitrification from single cell to ecosystem levels. *ISME Journal* **16**, 1647–1656 (2022).
21. Könneke, M. *et al.* Ammonia-oxidizing archaea use the most energy-efficient aerobic pathway for CO2 fixation. *Proc Natl Acad Sci U S A* **111**, (2014).
22. Stief, P. Stimulation of microbial nitrogen cycling in aquatic ecosystems by benthic macrofauna: mechanisms and environmental implications. *Biogeosciences* **10**, 7829–7846 (2013).

****************************************************

We greatly appreciate the reviewers for their support of this work, their attention to detail, and their constructive criticism. Below is a point-by-point response to the reviewers' comments, indicated in the red-type face. The grey-highlighted line numbers refer to changes made in the revised manuscript (e.g lines XX-XX). A complete list of references is included at the end of the response to reviewers' comments.

Reviewer Comments:

Reviewer #3 (Remarks to the Author):

Dear authors
thanks for addressing in the revised version of the manuscript my comments to the original version. I am in general happy with the way you improved the manuscript. I think there is room for future methodological improvements to turn your potential rates into in situ ones. I know that bioturbation makes the application of the IPT problematic, but there are a lot of good papers addressing benthic N cycling in bioturbated sediments, performed with combinations of net fluxes and r-IPT. I am surprised that with such high density of bioturbators potential rates of sediment nitrification are so low. NH4+ and O2 should not be limiting in such conditions and a large stimulation of nitrification is generally found.

We greatly appreciate the reviewer's constructive feedback, which prompted us to more clearly distinguish between potential rates (derived from slurry incubations) and in situ rates, and ultimately led to a more balanced discussion of sediment biogeochemical processes. The point regarding the advantages of working with intact sediment cores is well taken, and this approach will be considered in future surveys of these heavily bioirrigated sediments. With respect to nitrification, both the rate measurements presented here and a parallel study targeting nitrification-related genes (Baumann et al. 2022), indicate a general absence of nitrification activity in Lake Baldegg sediments. We propose that this absence reflects competitive exclusion, whereby nitrifying autotrophs are outcompeted for oxygen by heterotrophic microorganisms actively respiring the abundant organic matter present. Under these conditions, oxygen is rapidly consumed, creating an environment in which nitrifiers, given their relatively slow growth rates, are unlikely to become established, despite bioturbation-driven $O_2$ supply. For context, more oligotrophic systems, such as the neighboring Lake Sarnen, exhibit active nitrification turnover rates and harbor abundant nitrifier-associated genes, highlighting the strong dependence of sediment nitrification on organic matter reactivity in the sediments rather than O2 avaliability alone.

Reviewer #4 (Remarks to the Author):

The Aquatic EcoDynamics (AED) model is a decent tool capable of simulating phytoplankton growth under varying environmental conditions, including temperature, light, and nutrient availability. The model appropriately represents the transformation of fixed carbon into POC through direct phytoplankton mortality and indirect pathways via zooplankton and bivalve grazing.
We agree with the reviewer that the AED model has the capacity to simulate the water-column POC flux to the sediments associated with summer phytoplankton blooms. However, as highlighted both in the manuscript and by the reviewer, the model does not fully capture all components of the POC flux. In particular, certain POC inputs, most notably those associated with the mixed regime, are not represented in the current 1D model framework. This leaves room for improvement regarding model parameterization, which we address separately below.

However, Table S7 appears to duplicate the content of Table S6, which makes it difficult to evaluate the results without the specific parameters used to calibrate phytoplankton growth.

We thank the reviewer for highlighting this oversight, and we apologize for the error. We have now provided an updated Table S7, which now shows the default and calibrated phytoplankton parameters used in the model.

In general, modeled POC fluxes in aquatic systems are sometimes underestimated, typically due to unaccounted lateral transport or other physical processes. A moderate bias is therefore understandable and can be justified. In this study, however, the modeled POC fluxes are underestimated by more than an order of magnitude, particularly under mixed-regime conditions. This large discrepancy leads to an artificially significant difference in modeled POC flux and Rpoc:no3 between the stratified and mixed regimes, a pattern that is not evident in the observational data (Fig. 2c, i). The same issue is also evident for modeled $O_2$ fluxes.

We thank the reviewer for raising this important point regarding limitations of the model in simulating POC fluxes. The pronounced discrepancy between modeled POC fluxes under in the mixed and stratified regimes is most likely attributable to the absence of lateral POC refocusing in the base model. Here, we define POC refocusing as the lateral transport of particulate organic carbon (POC) from the lake margins toward the deepest point, a common feature of lakes with sloping bathymetry (Hilton et al. 1986; Evans 1994; Blais and Kalff 1995). Detailed analysis of our in situ sediment trap data (see below) clearly highlights the important role of lateral refocusing in redistributing POC within Lake Baldegg.

**Table 1: Percent improvement in reproducing observations when switching from the base to the "refocused" POC model.** Unless otherwise noted, values represent means. POC fluxes are reported in mmol m$^{-2}$ d$^{-1}$. Percent improvement is calculated as the relative reduction in absolute error compared to the base model: % Improvement = ((Base – Observed) - (Refocused - Observed)) / (Base – Observed) x 100. Variability around the mean is shown in the accompanying boxplots below.

| | Base model | Refocus model | Obs | Overall improvement |
|---|---|---|---|---|
| | Stratified regime | | | |
| POC | 10.7 | 15.0 | 20.8 | +43% |
| Rpoc:no3 | 5.9 | 8.2 | 9.5 | +64% |
| | Mixed regime | | | |
| POC | 0.5 | 12.9 | 17.7 | +72% |
| Rpoc:no3 | 0.2 | 5.0 | 3.6 | +59% |
| | Mixed vs Stratified | | | |
| POC | -95% | -14% | -15% | +99.95% |
| Rpoc:no3 | -97% | -39% | -62% | +99.82% |
| DNRA | -76% | -10% | -14% | +99.74% |
| Denitrification | +64% | +47% | +49% | +33.3% |

Importantly, we were able to link observed POC refocusing to wind-driven meteorological forcing, providing a clear physical mechanism for this process. Because the same meteorological data are used to drive the GLM-AED model, lateral POC refocusing could be incorporated into the base model in a physically consistent manner. The implementation of this process in the modified ("refocused") model substantially improved the agreement between modeled and observed POC fluxes, particularly during the mixed regime (Review Table 1). In the section below, we provide a detailed description of lateral POC transport, its coupling to wind-driven processes, and the resulting model performance in terms of POC fluxes, $R_{POC:NO3}$, estimates of denitrification and DNRA, and the responses of both the base and refocused models under future climate scenarios.

## Quantification of sediment refocusing and its implementation in the model

First, we highlight the magnitude of the discrepancy between modeled and observed POC fluxes in the base model across the two mixing regimes. During summer, the mean modeled POC flux (10.7 ± 19.2 mmol m$^{-2}$ d$^{-1}$) is approximately a factor of two lower than the observed flux (20.8 ± 6.8 mmol m$^{-2}$ d$^{-1}$). These summer values differ by approximately 0.5 standard deviations, corresponding to a small effect size in statistical terms (Cohen's $d \approx 0.2$–$0.5$). By contrast, the winter discrepancy is far more pronounced: the observed winter POC flux (17.7 ± 8.6 mmol m$^{-2}$ d$^{-1}$) exceeds the modeled value (0.5 ± 1.1 mmol m$^{-2}$ d$^{-1}$) by more than an order of magnitude, and the two distributions differ by roughly two standard deviations, indicating a statistically meaningful mismatch.

Observational evidence strongly supports substantial lateral POC refocusing, especially during the mixed period in Lake Baldegg. Specifically, the sediment trap deployed in the bottom waters (3 m above the sediment) at the deepest point of the lake generally recorded much higher POC fluxes than the trap positioned below the epilimnion at 15 m (Review Fig. 1a). This pattern is especially evident in late-summer and continues into the mixed period, when the lower trap report fluxes well in excess of those measured in the upper trap over the same time interval. Moreover, material collected during the mixed period was markedly depleted in

nitrogen relative to carbon, indicating a highly degraded organic matter signature (Review Fig. 1b). Together, these observations provide strong evidence for resuspension, lateral transport and refocusing of particulate material, likely degraded terrestrial and/or aquatic organic matter, from the lake margins toward the deepest point. To explicitly quantify this refocused POC flux, we calculated the difference between lower- and upper-trap POC fluxes (Review Fig. 1a, b). The seasonal trend finds that the POC refocusing flux is minimal in spring (Review Fig. 1b), but increases steadily through late summer and autumn, and remains high throughout the winter mixed period. Overall, the mean refocusing flux during the mixed period is approximately two-fold greater than during stratification (Review Fig. 1c).

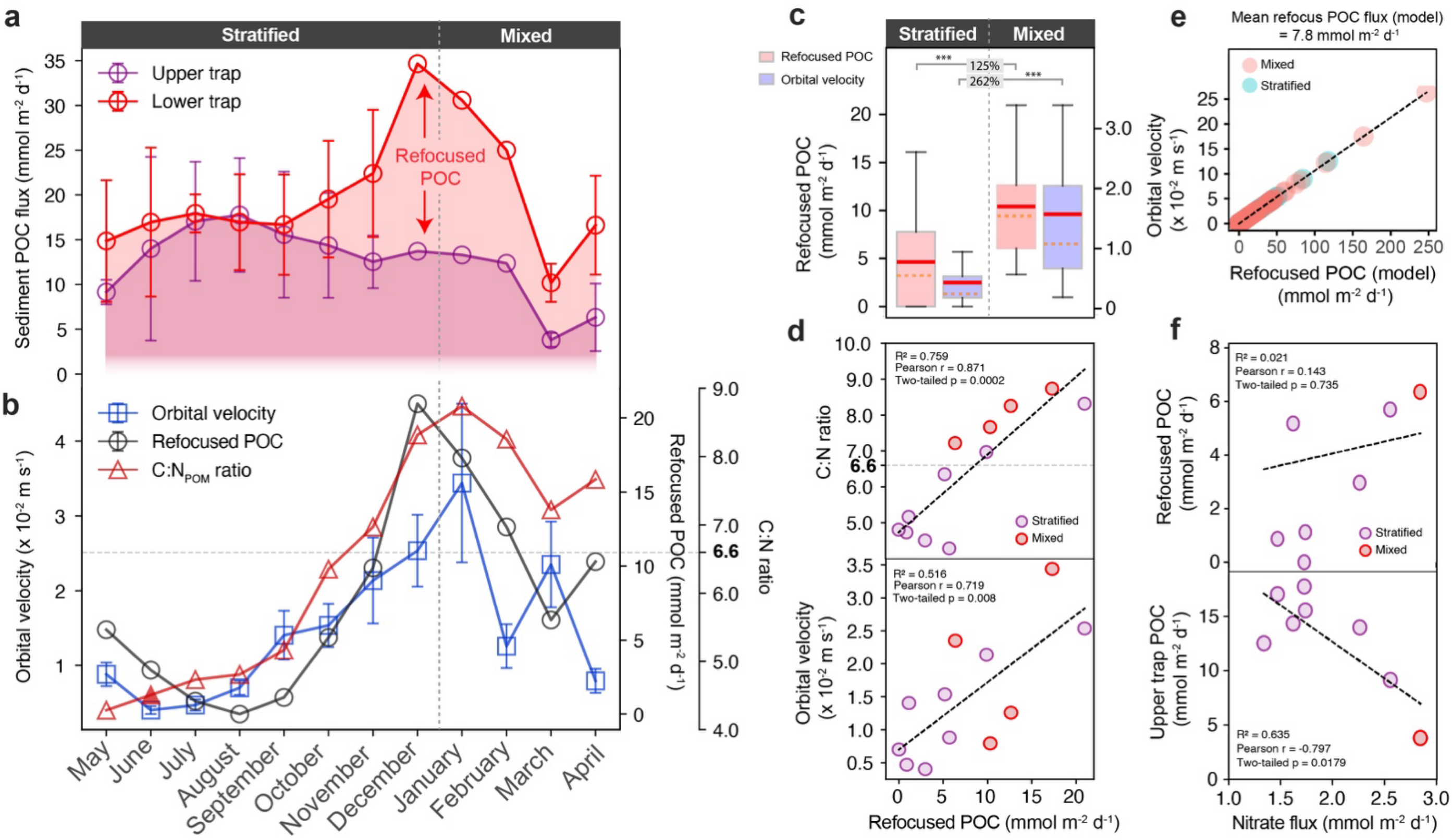


**Review Figure 1. Observations of sediment refocusing and its coupling to wind-driven meteorological data.** Panels (a, b) show seasonal variability in water-column POC flux derived from upper and lower sediment traps previously reported by Müller et al. 2021. The upper trap was positioned 15 m below the surface and reflects euphotic-zone-derived POC export, whereas the lower trap, located 3 m above the sediment, represents total POC flux (refocused/lateral plus vertical export). Refocused POC flux was calculated as the difference between lower and upper trap fluxes (panel b). Wind-driven near-bed orbital velocities derived from meteorological forcing, which are the same data used to force the GLM-AED model, are also shown in panel b. Seasonal changes in particulate organic matter C:N ratios are additionally shown, with the Redfield ratio (C:N = 6.6) indicated for reference. (c) Boxplot comparing refocused POC fluxes between stratified and mixed regimes. The mixed regime is defined as a temperature difference of ≤ 1 °C between surface and bottom waters (see Methods). Statistical differences between regimes were evaluated using a two-tailed Mann–Whitney test, with significance indicated as $p < 0.05$ (*), $p < 0.01$ (**), and $p < 0.01$ (***). (d, f) Correlation analyses between observed biogeochemical and physical parameters; Pearson correlation coefficients and *p*-values are reported. (e) Empirical calibration curve used in the model to relate refocused POC flux to wind-driven orbital velocities (based on meteorological data).

Importantly, the sediment POC refocusing flux was strongly correlated with wind-driven orbital velocities, a proxy for wave-generated near-bed shear stress, which is derived from the meteorological lake data (the same data used to force the GLM-AED model) (Review Fig. 1b, d; Pearson correlation, two-tailed $p < 0.01$; df = 12; $\alpha = 0.05$). This relationship indicates that POC refocusing is strongly coupled to wind forcing that, in turn, governs surface wave activity and thereby the near-bed orbital velocities that promote sediment resuspension and lateral transport. Because the 1D framework of the GLM-AED configuration does not include any representation of lateral transport, it lacks a built-in refocusing flux that, according to our observations, averages 7.8 mmol $m^{-2}$ $d^{-1}$ over the year and represents a substantial fraction of the POC flux not captured by the model.

Although such POC refocusing is not inherent to the base model, it can be incorporated indirectly through the physical coupling between wind-driven orbital velocities (already parameterized in GLM) and POC refocusing. We thus implemented this in the model using a calibration curve that applies a linear relationship

between orbital velocity and the refocused POC flux, whereby the delivery of this flux is scaled proportionally to the intensity of the modeled orbital velocities (Review Fig. 1e). As a result, the mean refocused POC flux assoicated with the model was 7.8 mmol m$^{-2}$ d$^{-1}$, matching the average value inferred from observations.

Incorporating this mechanism into the base model led to a substantially greater delivery of total POC to the sediments than in the original base model. During the mixed regime, the total modeled POC flux increased from 0.5 to 12.9 mmol m$^{-2}$ d$^{-1}$, bringing it much closer to the observed value of 17.7 mmol m$^{-2}$ d$^{-1}$ (Review Fig. 2a). Although the effect is strongest in the mixed period, owing to higher orbital velocities, improvements are also evident during the stratification period, where total modeled fluxes increased from 10.7 to 15.0 mmol m$^{-2}$ d$^{-1}$, approaching the observed summer flux of 20.8 mmol m$^{-2}$ d$^{-1}$. Overall, the refocused model shows clear improvements both in absolute flux magnitudes and in the contrast between mixed and stratified regimes, which now more closely mirrors the observed seasonal pattern, or lack thereof (Review Fig. 2a, b).

This approach is described in the revised Methods (lines 445–474). Review Figs. 1 and 2 are now included as Extended Data Figs. 7 and 8, and the main figure panels presenting modeled POC fluxes have been updated accordingly (Fig. 2c, d).

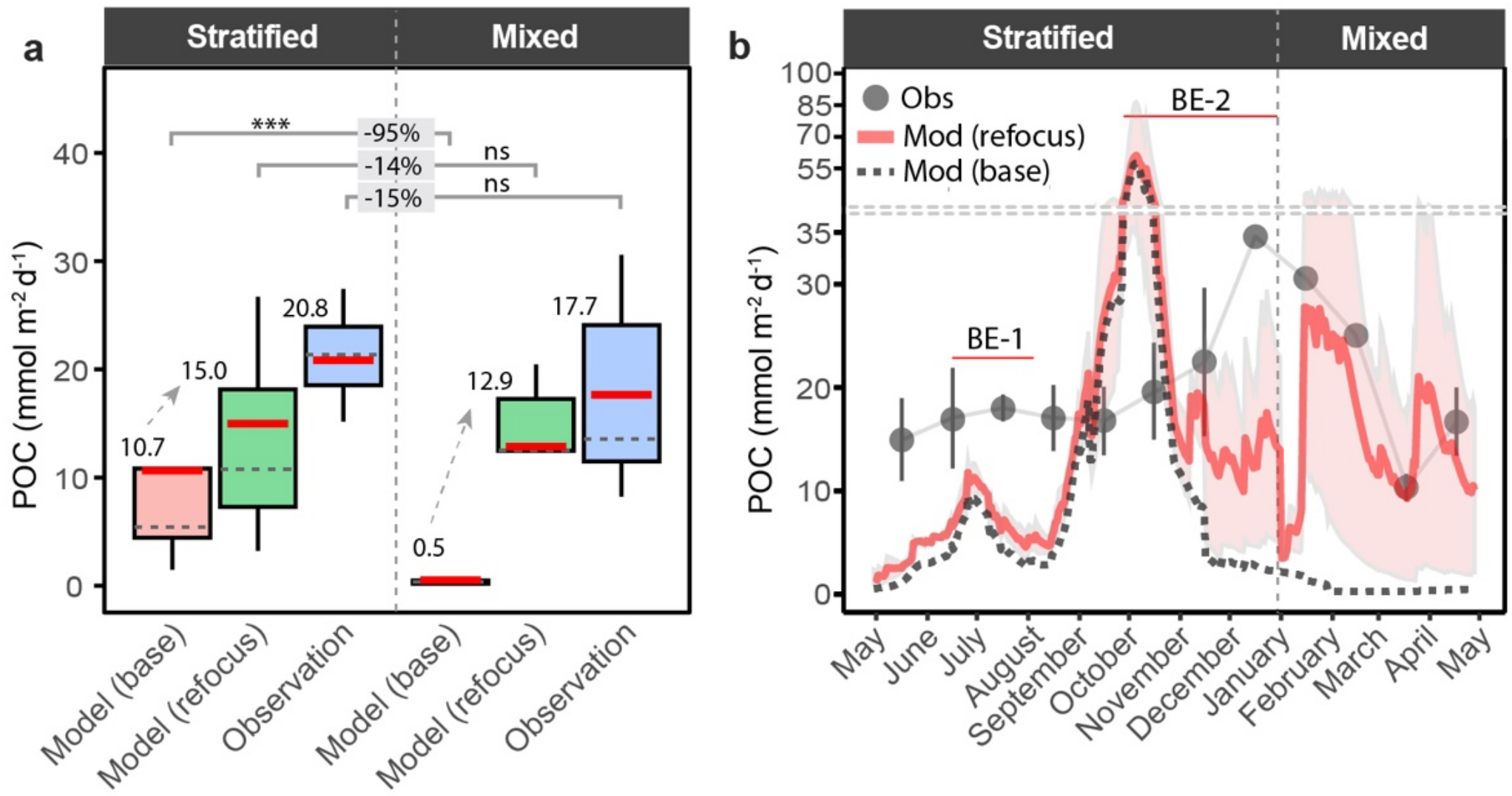


**Review Figure 2: Improvement in model–observation agreement for the POC flux following the transition from the base to the "refocused" model.** (a) Boxplot comparing the base model versus the refocused POC model and the observations for the respective stratified and mixed regimes. The mixed regime is defined as a temperature difference of ≤ 1 °C between surface and bottom waters (see Methods). Statistical differences between regimes were evaluated using a two-tailed Mann–Whitney test, with significance indicated as $p < 0.05$ (*), *p < 0.01 (**), and p < 0.01 (***)*. Solid red lines show means, and dotted lines indicate medians. Mixed-regime observations with limited sample size (n ≤ 10) include POC (n = 7); all other parameters have n > 10. (b) Seasonal variability of the POC flux. Two Bloom Events (BE-1 and BE-2) that were associated with high POC sedimentation are indicated.

The overall increase in modeled POC delivery resulting from the addition of refocused material could, in principle, be expected to alter background oxygen and nitrate fluxes. However, our observational data find that refocused POC does not exert a measurable control on the nitrate flux (Review Fig. 1f; Pearson correlation, two-tailed $p > 0.05$; df = 8; α = 0.05). This is likely because refocusing predominantly introduces more degraded, less biodegradable material – much of it of terrestrial origin – which has limited reactivity in the sediments. In contrast, a statistically significant relationship was observed between the upper-trap POC flux and the nitrate flux (Pearson correlation, two-tailed $p < 0.05$; df = 8; α = 0.05), indicating that the more labile fraction of settling POC is the primary driver of benthic nitrate dynamics. A similar pattern was found for oxygen fluxes. Together, these results support the conclusion that the incorporation of POC refocusing is unlikely to meaningfully alter the underlying nitrate and oxygen budgets in the model. Indeed, the model already reproduces observed regime contrasts well: oxygen fluxes differ by 31% between mixing regimes in observations and 35% in the model, while nitrate fluxes differ by 39% and 32%, respectively (Fig. 2e, g). This reasoning is now described in the revised Methods section (lines 478–485).

## Effect of POC refocusing on $R_{POC:NO3}$

The improved representation of lateral refocusing also has positive downstream effects on the simulated ratio between POC and nitrate ($R_{POC:NO_3}$). Specifically, this ratio increased from 5.9 to 8.2 when transitioning from the base to the refocused model, placing it within the range of the observed mean value of 9.49 for the stratified period (Review Fig. 3a). For the mixed regime, the modeled ratio rose from 0.2 to 5.1, compared with an observed value of 3.6, indicating that the model no longer strongly underestimates $R_{POC:NO_3}$ during this period. Seasonally, the "refocused" $R_{POC:NO_3}$ reproduces both bloom-related peaks and the low values associated with the mixed period, closely resembling the observed temporal pattern (Review Fig. 3b). This is briefly outlined in the revised Methods section (lines 474–477). Review Fig. 3 is included as Extended Data Fig. 8, and the main figure panels showing modeled $R_{POC:NO_3}$ have been updated accordingly (Fig. 2i, j).

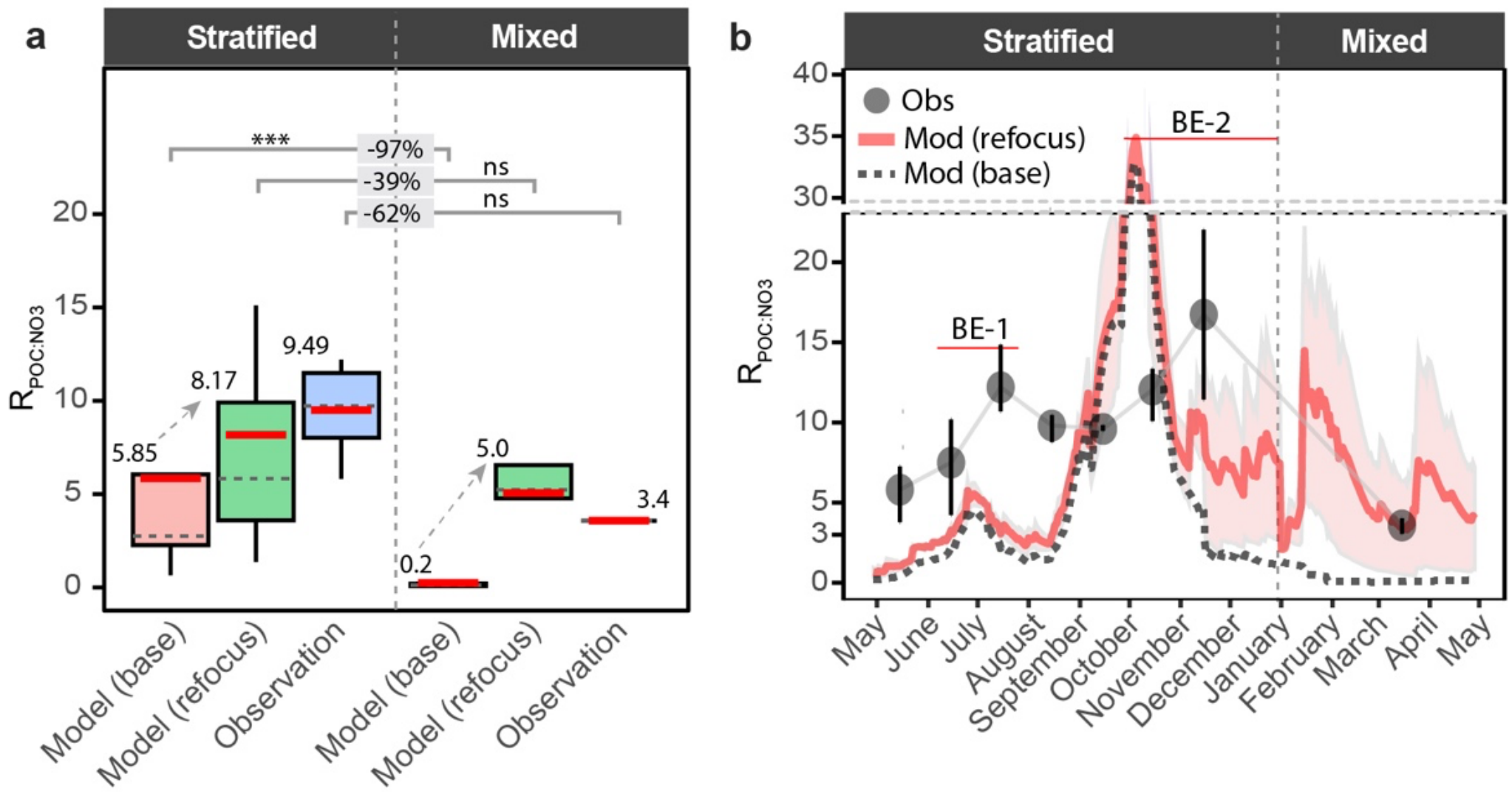


**Review Figure 3: Improvement in model–observation agreement for $R_{POC:NO_3}$ following the transition from the base to the "refocused" model.** (a) Boxplot comparing the base model versus the refocused POC model and the observations for the respective stratified and mixed regimes. The mixed regime is defined as a temperature difference of ≤ 1 °C between surface and bottom waters (see Methods). Statistical differences between regimes were evaluated using a two-tailed Mann–Whitney test, with significance indicated as $p < 0.05$ (*), $p < 0.01$ (**), and $p < 0.01$ (***). Solid red lines show means, and dotted lines indicate medians. Mixed-regime observations with limited sample size (n ≤ 10) include $R_{POC:NO_3}$ (n = 1); all other parameters have n > 10. No significant difference is denoted by (ns). (b) Seasonal variability of $R_{POC:NO_3}$.

## Effect of POC refocusing on DNRA and denitrification activity

With the $R_{POC:NO_3}$ ratio now in closer agreement with observations, this ultimately leads to a more accurate representation of the nitrate-reducing processes of denitrification and DNRA. In the "refocused" model, denitrification in the mixed regime declined slightly from 2.5 to 2.2 mmol $m^{-2}$ $d^{-1}$, while remaining nearly unchanged under the stratified regime (1.6 to 1.5 mmol $m^{-2}$ $d^{-1}$) relative to the base model (Review Fig. 4a, c). As a result, the modeled difference between mixed and stratified periods (47%) is now very close to the observed difference of 49%.

The improvement is even more pronounced for DNRA. In the refocused model, DNRA increased substantially in the mixed regime (from 0.02 to 0.4 mmol $m^{-2}$ $d^{-1}$) and showed a modest increase during stratification (from 0.3 to 0.4 mmol $m^{-2}$ $d^{-1}$) compared with the base model (Review Fig. 4b). Consequently, the modeled contrast between mixed and stratified regimes (10%) now closely matches the observed value (14%), representing a major improvement over the base model, which overestimated this difference (76%). From a seasonal perspective, the refocused model continues to reproduce the main patterns seen in the rate measurements, including the DNRA peaks associated with the BE-1 and BE-2 events and the elevated denitrification during the mixed period (Review Fig. 4c). Overall, activating POC refocusing reduces modeled denitrification during winter mixing and reallocates nitrate reduction toward DNRA, yielding a seasonal partitioning between DNRA and denitrification that more closely aligns with observations.

This is briefly outlined in the revised Methods section (lines 474–477). Review Fig. 4 is included as Extended Data Fig. 8, and the main figure panels showing modeled denitrification and DNRA have been updated accordingly (Fig. 2k, l, m, n).

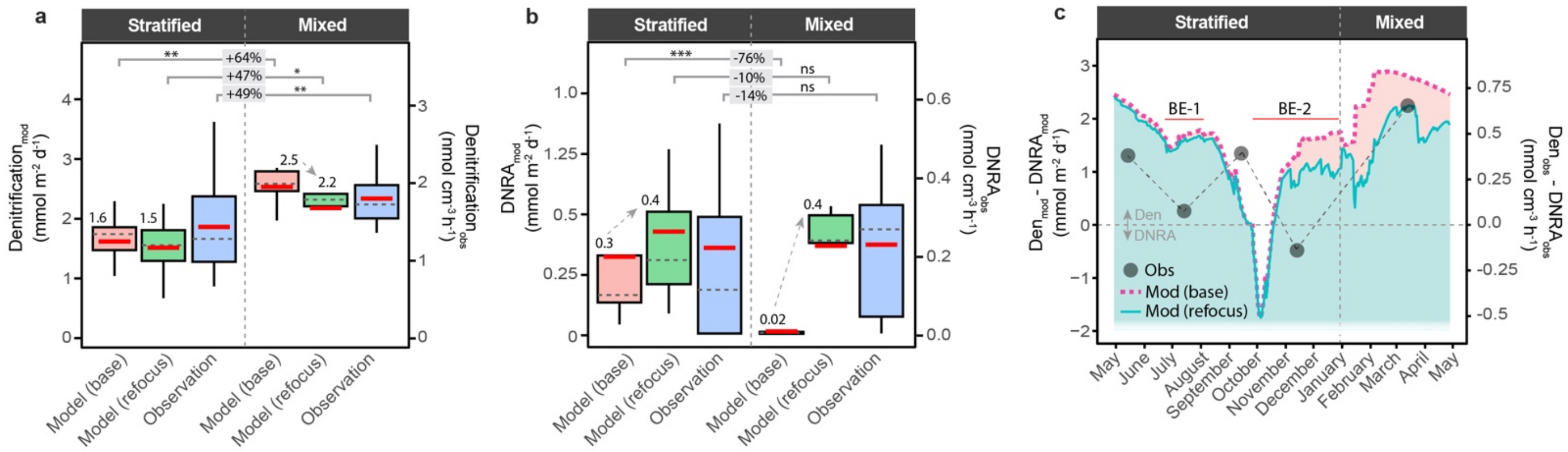


**Review Figure 4. Improvement in model–observation agreement for estimated sediment denitrification and DNRA following the transition from the base to the refocused model.** (a, b) Boxplots comparing the base model versus the refocused POC model and the observations for the respective stratified and mixed regimes. The mixed regime is defined as a temperature difference of ≤ 1 °C between surface and bottom waters (see Methods). Statistical differences between regimes were evaluated using a two-tailed Mann–Whitney test, with significance indicated as $p < 0.05$ (*), $p < 0.01$ (**), and $p < 0.01$ (***). No significant difference is denoted by (ns). Solid red lines show means, and dotted lines indicate medians. (c) Seasonal variability of sediment denitrification and DNRA activity.

## Effect of POC refocusing on future DNRA and denitrification activity

We next applied the same approach to the RCP scenarios by adding POC refocusing to the baseline model as a function of wind-driven orbital velocities. Under the worst-case scenario (RCP8.5 relative to RCP2.6 or to the historical baseline), the "refocused" model predicts a reduction in denitrification of approximately 8.3 to 13.3%, slightly greater than the 7.6 to 11.7% reduction estimated by the base model (a difference of ~1-2%; Review Fig. 5). This small difference arises because wind speeds, which drive POC refocusing, are expected to vary under future climate scenarios, leading to subtle changes in modeled POC redistribution and, consequently, in the underlying $R_{POC:NO3}$. Nevertheless, the overall projected change in denitrification under RCP8.5 remains small and largely consistent between the base and refocused model configurations.

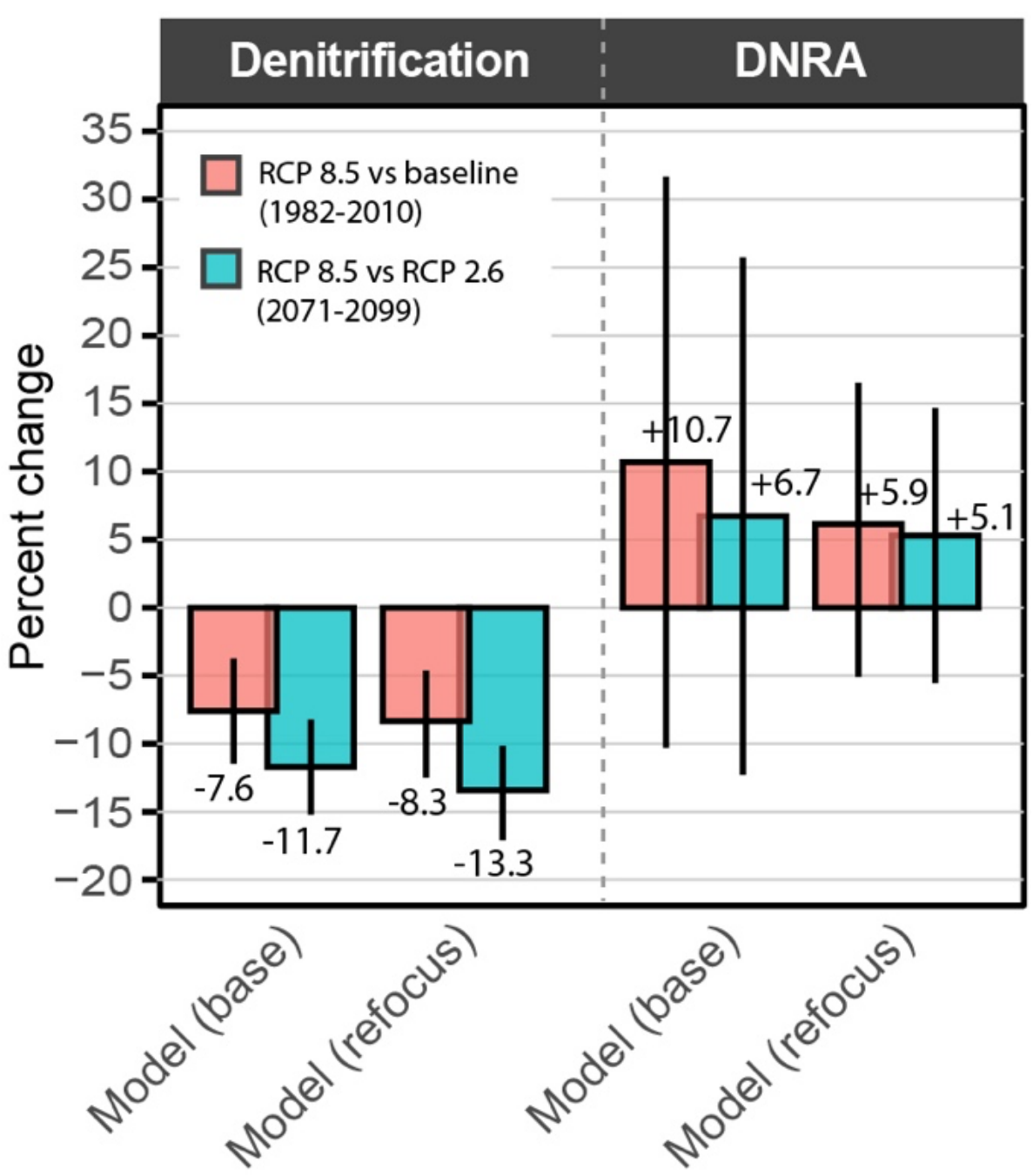


**Review Figure 5. Comparison of the base and refocused POC model configurations in projections of Lake Baldegg's future capacity to remove fixed nitrogen.** Error bars indicate the standard error (n = 29).

For DNRA, the refocused model predicts an increase of approximately 5.1–5.9% under RCP8.5 relative to RCP2.6 or to the historical baseline, compared with a 6.7–10.7% increase in the base model (a difference of 2–4%). This larger discrepancy between the base and refocused model projections, relative to that observed for denitrification (1–2%), reflects the substantially improved baseline representation of DNRA in the refocused model. Specifically, DNRA activity was much more strongly misrepresented in the original model (Review Table 1) than denitrification, such that incorporation of POC refocusing produces a more pronounced correction in projected DNRA responses (particularly for the mixed period).

Most importantly, the close agreement between the base and refocused model projections of denitrification **reinforces the conclusion that warming will substantially lower the lake's capacity to remove fixed nitrogen (by 8.3-13.3%),** and demonstrates that incorporating a more realistic representation of POC dynamics strengthens, rather than alters, the central conclusions of the study. The projected reduction in denitrification capacity is still attributed to the 27-day contraction of the mixed regime, which disproportionately contributes to denitrification. In addition, denitrification during the mixed period becomes further constrained by reduced nitrate availability (Extended Data Fig. 6c). The expansion of the stratified period enlarges the phytoplankton niche, enhancing dissolved inorganic

nitrogen assimilation in the water column and increasing organic matter burial, thereby diminishing nitrate resupply to sediments (now briefly clarified on lines 245-248 in the revised manuscript).

All values in the climate-scenario section (lines 22 and 227–274) have been updated accordingly in the revised manuscript. Review Fig. 5 is now presented as Extended Data Fig. 8e, and the main figure panels showing modeled denitrification and DNRA under future scenarios have been updated (Fig. 4b, c). Related future POC projections are shown in Extended Data Fig. 6a, b.

Given that the modeled and observed POC fluxes (and Rpoc:no3) differ by more than two orders of magnitude, a direct comparison between the two datasets is not meaningful.

As discussed above, we agree with the reviewer that the base 1D model did not adequately reproduce the POC flux observations, largely due to the absence of lateral POC refocusing (Review Fig. 1b–d). However, after implementing the refocused POC model, we observed a 43–72% improvement in model–observation agreement (Review Table 1). During the mixed regime, modeled POC flux increased from 0.5 to 12.9 mmol $m^{-2}$ $d^{-1}$ (base to refocused model), substantially reducing the discrepancy with the observed value of 17.7 mmol $m^{-2}$ $d^{-1}$ (Review Fig. 2b). Improvements were also evident during stratification, where the modeled POC flux increased from 10.7 to 15.0 mmol $m^{-2}$ $d^{-1}$ after refocusing, approaching the observed summer mean of 20.8 mmol $m^{-2}$ $d^{-1}$. Likewise, a similar improvement was observed for $R_{POC:NO3}$. Under stratified conditions, the modeled ratio increased from 5.9 to 8.2, placing it close to the observed mean of 9.49 (Review Fig. 2d). During the mixed regime, the ratio increased from 0.2 to 5.1, compared to an observed value of 3.6, indicating that the refocused model no longer strongly underestimates $R_{POC:NO3}$ during this period.

Furthermore, the modeled POC fluxes show extremely high variability (Fig. 2c, d), which in turn dominates the variability in the modeled Rpoc:no3.

As outlined above, this issue has now been addressed through the implementation of the refocused POC model. When data are grouped according to the mixed and stratified regimes, the modeled POC fluxes and $R_{POC:NO3}$ produced by the refocused model fall within a much more reasonable range of the observed data (Review Table 1; Review Figs. 2a, 3a).

Because the observed POC flux are usually much higher and exhibit much smaller variation than the modeled values, it is possible that a relative steady lateral transport may constitute the dominate source of POC flux in the lake.

We thank the reviewer for this important observation. Our re-analysis of the sediment-trap dataset confirms that lateral POC refocusing is indeed a major component of the carbon flux in Lake Baldegg (and of many lakes more genrally), accounting for ~40% of total annual POC delivery to the sediments. However, the refocused flux is not steady. Instead, it shows pronounced seasonality, with maximum values during the mixed regime (Review Fig. 1c). This timing coincides with periods of elevated wind-driven orbital velocities that enhance near-bed shear stress, sediment resuspension, and lateral transport (Review Fig. 1c, d). Thus, while lateral transport is large in magnitude, it fluctuates seasonally, rather than being a constant background flux.

By explicitly coupling refocused POC flux to modeled orbital velocities, we introduce a physically constrained mechanism that reproduces both the magnitude and the observed seasonal timing of lateral transport. As a result, absolute modeled POC fluxes during the mixed regime now closely match sediment-trap observations (Review Table 1), and the exaggerated stratified-mixed contrast seen in the base model is now remedied. The revised model, therefore, shows that lateral transport is a seasonally modulated process that can be mechanistically linked to hydrodynamic forcing, rather than an unvarying source term.

If lateral transport and other unrepresented processes indeed dominate POC flux dynamics in the lake,

then the model's attribution of POC flux variation to seasonal hydrological changes, and the consequent effects on denitrification and DNRA, may not be valid. In that case, the argument linking seasonal variation to these biogeochemical processes, as well as the future projections based on it, lacks sufficient support.

We agree with the reviewer that the original model configuration overrepresented seasonal variability in the POC flux because lateral POC refocusing was not represented. As a result, the base model simulated much higher POC delivery during stratification than during the mixed regime, which was inconsistent with sediment-trap observations. Implementing wind-driven POC refocusing resolves this issue: total modeled POC flux now includes both euphotic-zone-derived and laterally refocused components, producing seasonal contrasts that are more subdued and in close agreement with observations.

Importantly, inclusion of refocusing improves simulation of the $R_{POC:NO3}$ ratio. Once refocusing is incorporated, differences in $R_{POC:NO3}$ between mixing regimes arise primarily from changes in nitrate flux rather than from changes in POC supply (main Fig. 2g). Thus, seasonal contrasts in nitrogen cycling reflect hydrodynamically controlled nitrate availability, not variability in total POC delivery. This point is now highlighted in the revised main text on lines 143 to 148.

With this correction, the model now accurately reproduces observed regime-dependent behavior for POC flux, nitrate flux, the $R_{POC:NO3}$ ratio, and the resulting nitrogen transformation processes. Modeled process rates fall within observational ranges: denitrification contrasts between mixed and stratified regimes are 47% in the model versus 49% in observations (Review Table 1). Improvements are even more pronounced for DNRA: mixed-regime rates increase from 0.02 to 0.4 mmol $m^{-2}$ $d^{-1}$ and stratified rates from 0.3 to 0.4 mmol $m^{-2}$ $d^{-1}$, yielding a modeled regime contrast of 10% compared with the observed 14%. These results demonstrate that the revised model captures the correct seasonal controls on nitrogen transformation pathways.

As mentioned earlier, under RCP8.5 relative to RCP2.6, denitrification declines by 8.3–13.3% in the refocused model (vs 7.6–11.7% previously), and DNRA increases by 5.1–5.9% (vs 6.7–10.7%). Incorporating realistic lateral transport refines carbon flux representation but does not alter the fundamental seasonal controls or projected response to warming. The projected reduction in denitrification capacity is still driven by the 27-day contraction of the mixed regime, which disproportionately affects denitrification (see updated main Figure 4b). In addition, denitrification during the mixed period becomes further constrained by reduced nitrate availability (Extended Data Fig. 6c). The expansion of the stratified period enlarges the phytoplankton niche, enhancing dissolved inorganic nitrogen assimilation in the water column and increasing organic matter burial, thereby diminishing nitrate resupply to sediments. Inclusion of lateral transport, therefore, strengthens the mechanistic basis of the study and confirms that both the seasonal interpretation and the future projections remain robust.

To clarify those concerns, it would be useful if the authors could provide details explaining why the modeled POC fluxes are substantially lower and exhibit considerably higher variation than the observations, as well as the large temporal offset (2 months in POC flux). Nevertheless, the related argument concerning the relationship between denitrification and DNRA remains unsupported if the modeled results can not be significantly improved through parameter optimisation.

We thank the reviewer for highlighting the discrepancies between modeled and observed POC fluxes. As discussed above, we attribute the underestimation of POC flux, particularly during the mixed regime, to the absence of lateral POC refocusing in the original model configuration. We provide observational evidence supporting this interpretation and further investigate the underlying physical drivers of POC refocusing, which we empirically link to wind-driven orbital velocities (Review Fig. 1a, b, c, d).

Incorporation of this refocused POC flux substantially improves model performance, resulting in a **43–72%** enhancement in model–observation agreement (Review Table 1). Specifically, the updated model (1) yields a more subtle and observationally consistent contrast between mixed and stratified POC fluxes (Review Fig.

2a), (2) improves agreement between modeled and observed $R_{POC:NO3}$ (Review Fig. 3a), and (3) leads to a markedly improved representation of denitrification and (Review Fig. 4a), in particular, DNRA activity in the lake (Review Fig. 4b).

From a future-scenario perspective, projections from the refocused model **reinforce the conclusion that warming will substantially reduce the lake's capacity to remove fixed nitrogen by 8.3–13.3% (Review Fig. 5),** and demonstrate that incorporating a more realistic representation of POC dynamics strengthens—rather than alters—the central conclusions of the study.

**Revisions to the manuscript related to the implementation of the "refocused" model (detailed above)**

- The **Methods** section has been expanded (lines 445–485) to describe the implementation of the updated POC refocusing model.
- The **review figures** discussed above are now provided as **Extended Data Figs. 7 and 8**.
- The **main text** (lines 142–148) now directly compares outputs from the refocused model with observations, addressing the previous underestimation of POC flux.
- Results from the updated model are incorporated into **Figs. 2 and 4** and **Extended Data Fig. 6**.
- All values in the **climate-scenario section** (lines 22 and 227–274) have been revised accordingly. These changes produce only minor quantitative differences, generally showing slightly **higher denitrification losses**, and therefore further **strengthen** the manuscript's central conclusions.

**Response to reviewer - References**


Baumann, K. B. L. and others. 2022. Microbial Nitrogen Transformation Potential in Sediments of Two Contrasting Lakes Is Spatially Structured but Seasonally Stable. mSphere **0**: e01013-21. doi:10.1128/msphere.01013-21

Blais, J. M., and J. Kalff. 1995. The influence of lake morphometry on sediment focusing. Limnol. Oceanogr. **40**. doi:10.4319/lo.1995.40.3.0582

Evans, R. D. 1994. Empirical evidence of the importance of sediment resuspension in lakes. Hydrobiologia **284**. doi:10.1007/BF00005727

Hilton, J., J. P. Lishman, and P. V. Allen. 1986. The dominant processes of sediment distribution and focusing in a small, eutrophic, monomictic lake. Limnol. Oceanogr. **31**. doi:10.4319/lo.1986.31.1.0125

Müller, B., R. Thoma, K. B. L. Baumann, C. M. Callbeck, and C. J. Schubert. 2021. Nitrogen removal processes in lakes of different trophic states from on-site measurements and historic data. Aquat. Sci. **83**. doi:10.1007/s00027-021-00795-7

Reviewer #4 (Remarks to the Author):

I would like to thank the authors for addressing my previous comments regarding the mismatch between the observed and modeled POC fluxes (and other parameters) by introducing the refocusing model. The new model scheme greatly improves the results and supports the conclusion that refocused/lateral POC contributes substantially to the total flux to the sediment.

We agree that the refocused model provided a better representation of the in situ data from sediment traps; hence, we appreciate the reviewer for raising this discussion.

I am generally satisfied with the revised manuscript. However, it would be helpful if the authors could briefly discuss the artificially significant difference in the modeled oxygen flux between the stratified and mixed periods, and address how this might affect other model outcomes.

The model reproduces the average differences between the mixed and stratified regimes with comparable skill to other modeled parameters. In fact, regime contrasts in oxygen flux are well captured: observations indicate a 31% difference between mixing regimes, while the model simulates a 35% difference. Thus, in terms of magnitude, the model performs well.

We understand the reviewer's point regarding the outcome of the statistical comparison between regimes. Specifically, the model output yields a statistically significant difference between mixed and stratified periods, whereas the observational dataset does not show a robust statistical separation. This discrepancy is largely attributable to characteristics of the observational data.

First, oxygen fluxes during the stratified period exhibit substantial variability (ranging from 3.6 to 12.4 mmol $m^{-2}$ $d^{-1}$), which increases uncertainty in the statistical comparison. Second, and more importantly, the mixed regime is represented by a limited number of observations ($n = 5$, compared to $n = 25$ for the stratified period). This imbalance reduces statistical power and increases uncertainty in non-parametric tests such as the Mann–Whitney test. The limited sample size reflects the practical constraints of sampling during the mixed period, which is shorter in duration and often affected by adverse weather conditions that restrict field access.

From a biogeochemical perspective, however, the absolute differences between regimes (mentioned above) are small (only a difference of 4%), and the mean fluxes are comparable. Consequently, the integrated seasonal budgets are very similar. Therefore, although statistical significance differs between modeled and observed datasets due to sample size and variability, the magnitude of regime contrasts, and thus the implications for coupled downstream processes, remains consistent.
